# Selective stalling of human translation through small-molecule engagement of the ribosome nascent chain

**DOI:** 10.1371/journal.pbio.2001882

**Published:** 2017-03-21

**Authors:** Nathanael G. Lintner, Kim F. McClure, Donna Petersen, Allyn T. Londregan, David W. Piotrowski, Liuqing Wei, Jun Xiao, Michael Bolt, Paula M. Loria, Bruce Maguire, Kieran F. Geoghegan, Austin Huang, Tim Rolph, Spiros Liras, Jennifer A. Doudna, Robert G. Dullea, Jamie H. D. Cate

**Affiliations:** 1 Department of Molecular and Cell Biology, University of California, Berkeley, Berkeley, California, United States of America; 2 Pfizer Medicinal Chemistry, Cardiovascular, Metabolic and Endocrine Disease Research Unit, Pfizer Worldwide Research and Development, Cambridge, Massachusetts, United States of America; 3 Primary Pharmacology Group, Pharmacokinetics, Dynamics and Metabolism, Pfizer Worldwide Research and Development, Groton, Connecticut, United States of America; 4 Pfizer Medicinal Chemistry, Cardiovascular, Metabolic and Endocrine Disease Research Unit, Pfizer Worldwide Research and Development, Groton, Connecticut, United States of America; 5 Drug Safety Research & Development, Pfizer Worldwide Research & Development, Andover, Massachusetts, United States of America; 6 Pfizer Medicinal Chemistry, Structural Biology and Biophysics, Pfizer Worldwide Research and Development, Groton, Connecticut, United States of America; 7 Pfizer Medicinal Chemistry, Computational Sciences, Pfizer Worldwide Research and Development, Cambridge, Massachusetts, United States of America; 8 Cardiovascular, Metabolic and Endocrine Disease Research Unit, Pfizer Worldwide Research and Development, Cambridge, Massachusetts, United States of America; 9 QB3 Institute, University of California, Berkeley, Berkeley, California, United States of America; 10 Department of Chemistry, University of California, Berkeley, Berkeley, California, United States of America; 11 Physical Biosciences Division, Lawrence Berkeley National Laboratory, Berkeley, California, United States of America; 12 Howard Hughes Medical Institute (HHMI), University of California, Berkeley, Berkeley, California, United States of America; Stanford University, United States of America

## Abstract

Proprotein convertase subtilisin/kexin type 9 (PCSK9) plays a key role in regulating the levels of plasma low-density lipoprotein cholesterol (LDL-C). Here, we demonstrate that the compound PF-06446846 inhibits translation of PCSK9 by inducing the ribosome to stall around codon 34, mediated by the sequence of the nascent chain within the exit tunnel. We further show that PF-06446846 reduces plasma PCSK9 and total cholesterol levels in rats following oral dosing. Using ribosome profiling, we demonstrate that PF-06446846 is highly selective for the inhibition of PCSK9 translation. The mechanism of action employed by PF-06446846 reveals a previously unexpected tunability of the human ribosome that allows small molecules to specifically block translation of individual transcripts.

## Author summary

Many disease-mediating proteins have proven difficult to target with traditional small-molecule pharmaceuticals. In this paper, we report that a small molecule, PF-06446846, directly inhibits translation of one such protein, proprotein convertase subtilisin/kexin type 9 (PCSK9), by acting on the translating human ribosome. PF-06446846 causes the translating ribosome to stall soon after translating the PCSK9 signal sequence. We further show that PF-06446846 activity is dependent on the amino acid sequence of the nascent chain inside the ribosome exit tunnel. In a rat safety study, we observe decreases in plasma PCSK9, total cholesterol, and low-density lipoprotein (LDL) cholesterol. Using mass spectrometry in cell culture and ribosome profiling, we demonstrate that despite acting on the ribosome, which synthesizes every protein in the cell, PF-06446846 displays a high level of selectivity for PCSK9. This unexpected potential for small molecules to selectively inhibit the human ribosome opens the possibility for future development of small molecules targeting disease-mediating proteins that were previously thought to be undruggable.

## Introduction

Reduction of plasma low-density lipoprotein cholesterol (LDL-C) through the use of agents such as statins represents the therapeutic standard of care for the prevention of cardiovascular disease (CVD) [1, 2], the leading cause of death in Western nations. Proprotein convertase subtilisin/kexin type 9 (PCSK9) regulates plasma LDL-C levels by preventing the recycling of the LDL-receptor (LDLR) to the plasma membrane of hepatocytes [3, 4]. Humans with natural PCSK9 loss-of-function mutations display dramatically reduced LDL-C levels and decreased risk of CVD, yet display no adverse health effects [5–8]. The robust LDL-C lowering observed with recently approved PCSK9 monoclonal antibodies (mAbs) when administered as a monotherapy or in combination with established LDL-C–lowering drugs validates the therapeutic potential of inhibiting PCSK9 function [9–11]. However, these therapeutic candidates require a parenteral route of administration rather than being orally bioavailable. Utilizing a phenotypic screen for the discovery of small molecules that inhibit the secretion of PCSK9 into conditioned media, we have recently identified a compound family that inhibits the translation of PCSK9 [12]. However, the mechanism of translation inhibition exerted by these compounds remains unknown. Herein we describe a more optimized small molecule, PF-06446846, that demonstrates in vivo activity. We show that PF-06446846 induces the 80S ribosome to stall while translating PCSK9. We further demonstrate using ribosome profiling that despite acting through protein translation, a core cellular process, PF-06446846 is exceptionally specific, affecting very few proteins. The PF-06446846 mechanism of action reveals a previously unexpected potential to therapeutically modulate the human ribosome with small molecules as a means to target previously “undruggable” proteins.

## Results

### PF-06446846 inhibits PCSK9 translation by causing the ribosome to stall during elongation

**Fig 1:**
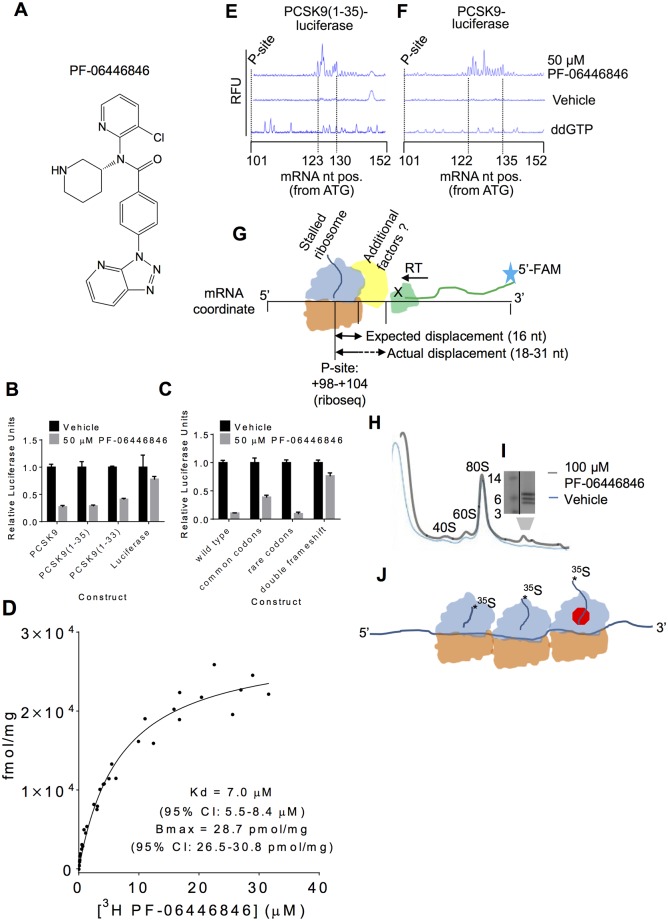
PF-06446846 targets the human ribosome, inducing stalling during proprotein convertase subtilisin/kexin type 9 (PCSK9) translation. (A) Structure of PF-06446846. (B) Luciferase activity of HeLa-based cell-free translation reactions programmed with mRNAs encoding PCSK9-luciferase, PCSK9(1–35)-luciferase, and PCSK9(1–33)-luciferase fusions and luciferase alone in the absence (black bars) or presence (grey bars) of 50 μM PF-06446846. All error bars represent one standard deviation of three replicates. (C) PF-06446846 sensitivity dependence on the amino acid sequence of PCSK9(1–33). PCSK9-luciferase fusions encode the native PCSK9 amino acid sequence with common codons or rare codons or a native, double-frameshifted mRNA sequence that results in a changed amino acid sequence (See S1E Fig for sequences). All error bars represent one standard deviation of three replicates. (D) ^3^H-PF-06446846 binding to purified human ribosomes, K_d_: 7.0 μM (95% CI: 5.5–8.4) B_max_: 28.7 pmol/mg (95% CI: 26.5–30.8). The symbols within the graph represent the individual measurements obtained from three independent experiments. B_max_ and K_d_ values were calculated using GraphPad PRISM, in which the complete (*n* = 3) dataset was fit to the one site-specific binding equation. (E–F) Electrophoreograms of toeprints of stalled ribosomes on the (E) PCSK9(1–35)-luciferase fusion construct and (F) full-length PCSK9-luciferase fusion. The nucleotide (nt) positions from the “A” of the ATG initiation codon of the first and last of the group of toeprinting peaks are indicated. The expected position of the P-site of the stalled ribosome from ribosome profiling is also indicated. (G) Schematic of ribosomal toeprinting assays. 5ʹ 6-FAM labeled primers are extended by reverse transcriptase, which terminates when blocked by a ribosome. In this case, we also hypothesize that additional factors may be bound to the stalled ribosome, obstructing the reverse transcriptase at more 3ʹ positions and over a broader range of positions then what is normally observed [13]. (H) Sucrose density gradient profiles of cell-free translation reactions programmed with an mRNA encoding an N-terminally extended PCSK9 in the presence of 100 μM PF-06446846 (grey) and vehicle (blue). (I) Tris-Tricine SDS-PAGE gels showing ^35^S-Met-labelled peptides that sediment in the polysome region of the gradient. (J) Model of the species isolated by density gradient centrifugation containing one stalled ribosome and two queued ribosomes. The individual quantitative observations that underlie Fig 1B–D are in S14 Table.

The previously identified hit compound was adequate for initial in vitro characterization, but in vivo assessment required improvements in pharmacokinetic properties [12]. The potency, physicochemical properties, and the off-target pharmacology associated with the hit compound were improved by structural changes to two regions of the molecule. These efforts led to the identification of compound PF-06446846 (Fig 1A), which has properties suitable for both in vitro and in vivo evaluation (S1 Fig and S1 Table). The synthesis and physiochemical characterization of PF-06446846 are described in the Materials and methods, S2–S8 Figs and S2–S7 Tables. PF-06446846 inhibited the secretion of PCSK9 by Huh7 cells with an IC_50_ of 0.3 μM (S1A Fig). However, metabolic labeling of Huh7 cells with ^35^S-Met/Cys showed that decreases in PCSK9 were not a consequence of global inhibition of protein synthesis (S1B and S1C Fig). Furthermore, proteomic analysis of the Huh7 cells utilizing stable isotope labeling with amino acids in cell culture (SILAC) indicated no general effect of PF-06446846 on the secreted and intracellular proteome (S9 Fig, S8–S10 Tables). Taken together, these results indicate that PF-06446846 exhibits a high degree of specificity for inhibiting the expression of PCSK9.

To identify the specific mechanism responsible for translation inhibition by PF-06446846, we tested mRNAs encoding PCSK9-luciferase fusions in HeLa cell–derived in vitro translation assays [12]. PF-06446846 inhibited translation of PCSK9-luciferase fusion constructs containing only the first 35 residues of PCSK9 and displayed comparable activity towards the first 33 residues (Fig 1B). In the HeLa cell-free translation assay, PF-06446846 inhibited PCSK9(1–35)-luciferase expression with an IC_50_ of 2 μM, while at the maximum concentration evaluated, 250 μM, the translation of luciferase without the PCSK9 N-terminal sequence was only inhibited by 20% (S1D Fig). Translation of the protein fusion constructs was driven by the encephalomyocarditis virus internal ribosome entry site (EMCV IRES), indicating that PF-06446846 is unlikely to target PCSK9 translation initiation directly. When all the codons of PCSK9(1–33) were mutated to either common or rare synonymous codons (S1E Fig), PF-06446846 still inhibited translation of PCSK9(1–33)-luciferase (Fig 1C), ruling out a role of the mRNA sequence. Conversely, PF-06446846 did not inhibit translation of a PCSK9(1–33) construct with two compensatory frameshifts that result in a near endogenous mRNA sequence but a nonendogenous amino acid sequence (Fig 1C and S1E Fig). These data indicate that PF-06446846 sensitivity is primarily dependent on the amino acid sequence of PCSK9. To further define the sequence requirements, we tested the activity of PF-06446846 against sets of N-terminal deletions, C-terminal deletions, and alanine scanning mutations of PCSK9(1–33). The most important regions in PCSK9(1–33) that confer sensitivity to PF-06446846 are Leu15–Leu20, residues 9–11 (which include two tryptophan amino acids) and residues 31–33 (S10A and S10B Fig). However, most mutations partially reduced the activity of PF-06446846, suggesting that multiple amino acid features of PCSK9(1–35) make contributions to its sensitivity to PF-06446846.

The sensitivity of the extreme N-terminal sequence of PCSK9 to PF-06446846 suggests that PCSK9(1–35) may act as a small molecule–induced arrest peptide in the ribosome exit tunnel, similar to arginine attenuator peptide, TnaC, ErmCL, and CatA86 [14]. [^3^H]PF-06446846 binds to purified human ribosomes (K_d_ = 7 μM) in filter-binding assays (Fig 1D). In ribosomal toeprinting assays [13] of cell-free translation reactions programmed with full-length PCSK9 and PCSK9(1–35)-luciferase fusions, 50 μM PF-06446846 induced reverse-transcriptase early termination products consistent with stalling on and after codon 35 (Fig 1E and 1F). In typical ribosomal toeprinting assays, 1–3 major peaks appear 14–16 nucleotides (nt) 3ʹ of the +2 position of the codon in the P-site [13]. In the case of PF-06446846–stalled ribosomes, the toeprinting peaks appeared in up to 14 nucleotide positions, depending on the construct used, and were 18–31 nucleotides 3ʹ of the +2 positions of the P-site codon, determined using ribosome profiling. The broader distribution of toeprint positions is consistent with the ribosome stalling at multiple codons, as indicated by ribosome profiling. The 3ʹ shift of the toeprint peaks relative to the ribosome profiling stall sites could be due to additional cellular factors recognizing and binding to the PF-06446846–stalled ribosomes (Fig 1G). In cell-free translation reactions programmed with mRNA encoding full-length PCSK9 fused to an N-terminal extension, PF-06446846 also resulted in the appearance of polysomes containing three small radiolabeled peptides, suggesting that these PCSK9 nascent chains associated with one stalled ribosome, followed by two queued ribosomes (Fig 1H–1J). Most arrest peptides function only in one domain of life—i.e., solely in bacteria or solely in eukaryotes [14]. In agreement with this phenomenon, PF-06446846 inhibited PCSK9(1–35)-luciferase translation in cell-free translation systems derived from rabbit reticulocytes, wheat germ, and yeast but not from *Escherichia coli* (S10C–S10F Fig).

### PF-06446846 reduces circulating PCSK9 and total plasma cholesterol levels in vivo

**Fig 2:**
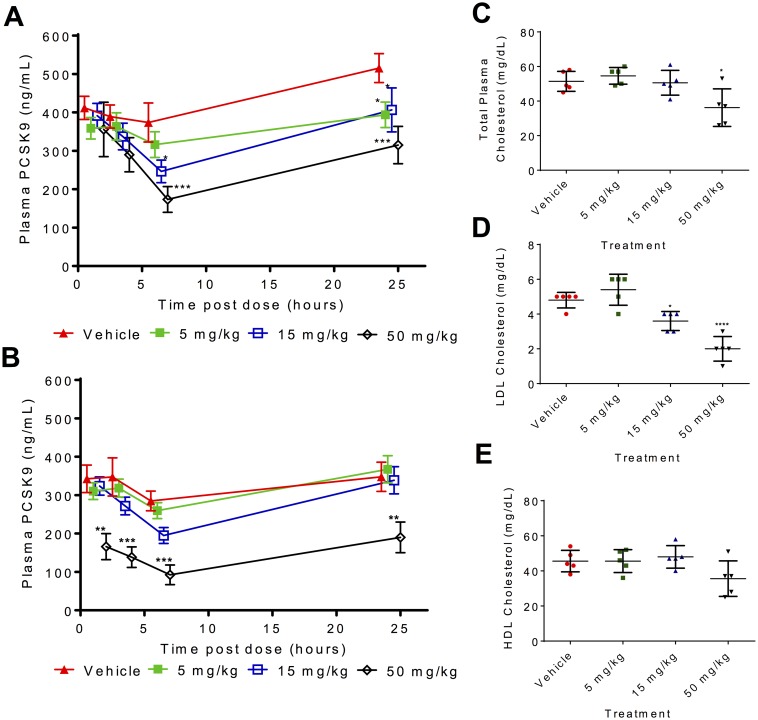
Oral administration of PF-06446846 reduces plasma proprotein convertase subtilisin/kexin type 9 (PCSK9) and total cholesterol levels in rats. (A–B) Plasma PCSK9 levels following (A) a single and (B) 12 daily oral doses of PF-06446848. Rats were administered the indicated dose of PF-06446846, and plasma concentrations of PCSK9 were measured by commercial ELISA at 1, 3, 6, and 24 h after dosing (A) or the 12th daily dose (B). Symbols represent mean concentration ± standard error and were jittered to provide a clearer graphical representation. Data were analyzed using a mixed model repeated measure (MMRM) with treatment, day, and hour as fixed factors; treatment by day and hour as an interaction term; and animal as a random factor. The significance level was set at a level of 5%. No adjustment for multiple comparisons was used. *p ≤ 0.05, **p ≤ 0.01, ***p ≤ 0.001. (C–E) Total plasma (C), low-density lipoprotein (LDL) (D), and high-density lipoprotein (HDL) (E) cholesterol levels in rats measured 24 h following 14 daily oral doses of PF-06446846. Symbols represent individual animal values. The middle horizontal bar represents the group mean ± standard deviation. Difference between group means relative to vehicle was performed by a one-way ANOVA followed by a Dunnett’s multiple comparisons test; * *p* ≤ 0.05, **** *p* ≤ 0.0001. The individual quantitative observations that underlie Fig 2 are in S14 Table.

To explore the safety of the compound and to gain insight into the in vivo activity of PF-06446846, male rats were orally administered PF-06446846 at doses of 5, 15, and 50 mg/kg daily for 14 d. Plasma PF-06446846 (S11 Table) and PCSK9 concentrations were measured at 1, 3, 6, and 24 h following the 1st and 12th dose, and total plasma cholesterol levels were assessed in fasted animals just prior to necropsy on day 15. Dose-dependent lowering of plasma PCSK9 was observed following single and repeated dosing of PF-06446846 (Fig 2A and 2B). In addition to the reduction in circulating levels of PCSK9, evidence of inhibition of PCSK9 downstream function was observed at the 50 mg/kg dose, with a statistically significant 30% decrease in total plasma cholesterol and 58% decrease in LDL cholesterol but no significant decrease in high-density lipoprotein (HDL). This encouraging effect on circulating PCSK9 concentration was achieved in the absence of modulating plasma liver function markers, with no treatment-related changes observed for alanine transaminase (ALT), aspartate transaminase (AST) or albumin (Fig 2C–2E and S11 Fig). Although reductions in cholesterol were observed, this endpoint is poorly assessed in rodents, and the clinical experience shows that reduced free PCSK9 levels represent the primary determinant of the improved lipid profile in humans [5–9, 11].

During the 2-wk dosing period, PF-06446846 was tolerated. There was a small decrease (11%–13% relative to vehicle) in food consumption at 50 mg/kg PF-06446846 that was not associated with any changes in body weight. Histological and clinical chemistry examination of samples collected at day 15 indicated no dose-limiting changes compared to vehicle at the 5 and 15 mg/kg dose level. Administration of PF-06446846 at 50 mg/kg/day demonstrated a minimal decrease in bone marrow cellularity (primarily involving erythroid parameters) that correlated with a decrease in red cell mass (9%). Furthermore, mild reductions in white blood cells (52%), lymphocytes (54%), T cell (approximately 54% in the total, helper, and cytotoxic T cells) and B cell populations (58.4%), as well as minimal necrosis of the crypt cells of the ileum (in one of five animals), were observed at 50 mg/kg/day. Importantly, no histopathological findings were observed at any dose of PF-06446846 in the liver, the organ responsible for the majority of PCSK9 production as well as maintaining whole-body cholesterol homeostasis [15, 16].

### Translation-focused, genome-wide identification of PF-06446846 sensitive genes

While the absence of adverse histopathological changes is consistent with the high degree of selectivity observed in SILAC experiments (S9 Fig), we used ribosome profiling [17–19] to provide a higher-resolution understanding of the selectivity of PF-0644846 at the level of mRNA translation. Huh7 cells were treated with 1.5 and 0.3 μM PF-06446846 (5x and 1x the IC_50_ in Huh7 cells) (Fig 1A) or a vehicle control for 10 and 60 min in three biological replicates, and ribo-seq libraries were prepared. To measure mRNA abundance and translational efficiencies, we subsequently conducted a second ribosome profiling study in which Huh7 cells were treated with 1.5 μM PF-06446846 or vehicle for 1 h, and both ribo-seq and mRNA-seq libraries were prepared from the same sample.

**Fig 3:**
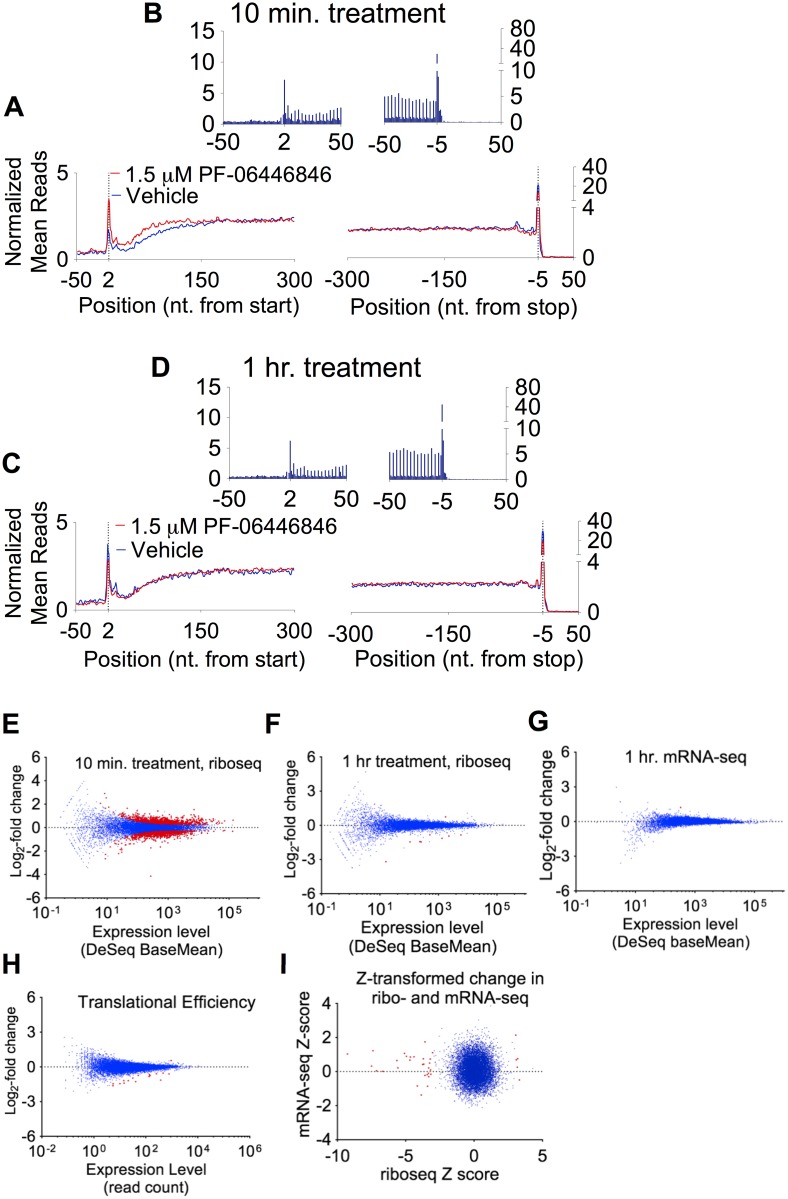
PF-06446846 does not cause widespread ribosomal stalling. (A–D) Metagene plots showing the normalized mean read counts at the nucleotide (nt) positions relative to the start and stop sites for cells treated with 1.5 μM PF-06446846 (red trace) and vehicle (blue trace) for (A–B) 10-min treatment and (C–D) 1-h treatment. In panels A–D, reads positions are displayed according to the inferred ribosomal P-site [19]. In panels A and C, the values are averaged over three nucleotides for clarity. Panels B and D represent zoomed views of the treatment datasets only to show three-nucleotide periodicity and preferential mapping to coding DNA sequence (CDS) regions, the hallmark features of ribosome profiling data. The normalized mean reads (NMR) were calculated as in [20]. The normalized read count at a given position on a particular mRNA is the number of reads aligning to that position divided by the average read density along the CDS. These values are then averaged across all transcripts of sufficient length. (E–G) Log_2_-fold change in the number of reads mapping to a given gene plotted against overall expression level of that gene as calculated by DeSeq. (E) 10-min treatments, (F) 1-h treatments, (G) mRNA-seq datasets for the translational efficiency (TE) data. Genes with a significant (false discovery rate [FDR] < 10%) change in expression are highlighted in red. (H–I) Changes in expression level are primarily due to changes in translation, not transcript levels. (H) Changes in TE plotted against expression level (read count). Red points indicate outliers (expression-level Z-score > 3.0) [22]. All changes in TE with Z-score > 3 are decreases. (I) Z-score–transformed change in ribo-seq read counts upon PF-06446846 treatment, plotted against the Z-score–transformed change in mRNA-seq read counts. The outliers (red) are spread solely along the *x*-axis, consistent with PF-06446846 expression level changes occurring primarily at the level of translation. For a further description of the Z-score transformation, see the Materials and methods and [22].

Metagene analysis [20, 21] of the distribution of ribosomal footprints relative to the start and stop codons displayed the hallmarks of ribosome footprints [18, 19], including 3-nt periodicity through the coding DNA sequence (CDS) regions and a minimal number of reads mapping to 3ʹ-UTR regions (Fig 3A–3D and S12 Fig). We also observed high correlation between replicates in reads aligning to individual genes (S13 Fig). We consistently observed a compound-independent depletion of reads aligning to the first forty codons of the CDS regions, a large buildup of reads on the stop codon, and a small queued ribosome peak upstream from the stop codon [22] (Fig 3A–3D and S12 Fig). This buildup may be due to omission of the cycloheximide pretreatment step [22–24], which we omitted to avoid an artefactual buildup of reads near the start codon [19]. PF-06446846 treatment induced no change in the average distribution of reads along the CDS after 1 h of treatment (Fig 3C) and only a small PF-06446846–induced accumulation of reads mapping to the first 50 codons after a 10 min treatment (Fig 3A), indicating that PF-06446846 does not cause pausing or stalling on most transcripts. The PF-06446846–induced increase in early read counts at the 10 min timepoint, but not at the 60 min timepoint, may indicate the induction of cellular stress upon the initial exposure to PF-06446846 followed by adaptation within the 1 h timeframe.

Differential expression analysis using the ribosome footprint data revealed that 1,956 genes are differentially expressed after 10 min of treatment at a false discovery rate (FDR) of 10% (DeSeq) but only 9 after 1 h (Fig 3E and 3F). Only a single gene, EFNA3, displayed a difference in transcript levels (FDR < 10%). mRNA-seq and translational efficiency (TE) analysis reveals that almost all of the change in expression levels occurs at the translation step (Fig 3G–3I). The two features present in the data at 10 min but not at 1 h—the relative global depletion of reads between nt positions +2 and +100 of the CDS and the large number of genes displaying a brief change in translation levels—could be indicative of cellular stress after 10 min of treatment. Thus, we focused the following analyses generally on the 1-h treatment time.

**Fig 4:**
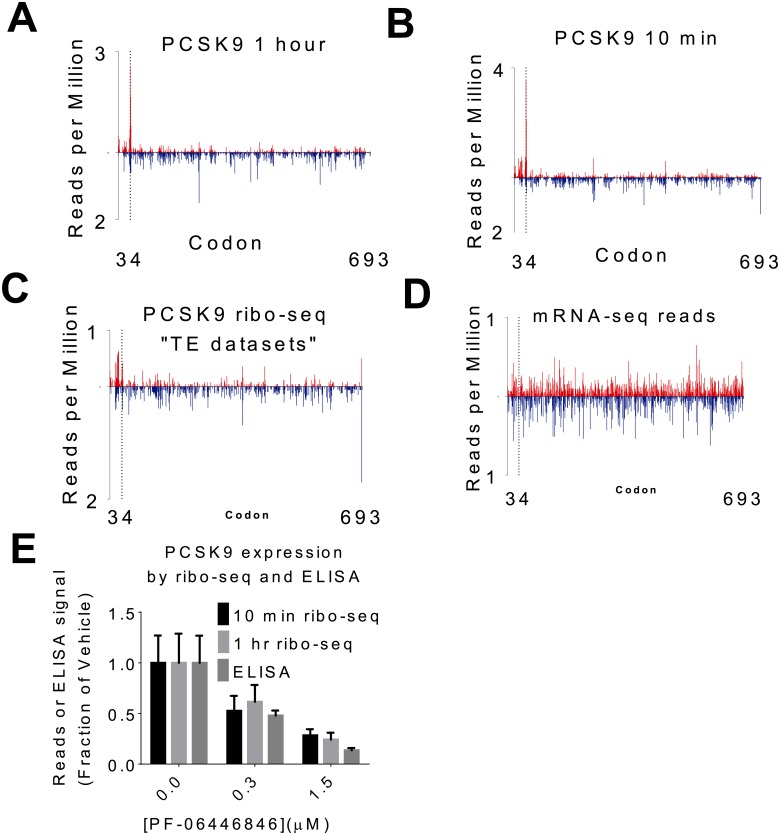
The PF-06446846–induced stall site is revealed by ribosome profiling. (A–D) Ribosome footprint density plots displaying the number of reads aligning to a given codon per million total reads for the proprotein convertase subtilisin/kexin type 9 (PCSK9) coding region from Huh7 cells treated for (A) 1 h and (B) 10 min. (C) Ribo-seq datasets from the second study and (D) mRNA-seq datasets from the second study. The upward red bars indicate readmaps from cells treated with 1.5 μM PF-06446846 and the blue downward bars represent vehicle. In panels A–D, read positions are mapped according to inferred location of the ribosome P-site [19]. (E) The footprint density downstream from the stall in the 10-min treatment (black) and 1-h treatment (light grey) compared with PCSK9 expression as measured by ELISA (middle grey). Error bars represent one standard deviation of three replicates. The individual quantitative observations that underlie Fig 4E are in S14 Table.

Examination of ribosomal footprints aligning to the PCSK9 transcript (Fig 4A–4C) revealed a PF-06446846–dependent buildup of footprints centered on codon 34, consistent with ribosomal stalling at this position. The number and distribution of fragments from mRNA-seq was unaffected by PF-06446846 (Fig 4D). PF-06446846 treatment also resulted in a decrease in ribosome footprints mapping 3ʹ to the stall site on the PCSK9 transcript. Notably, the magnitude of the decrease in footprints 3ʹ to the stall site is similar to the level of inhibition of PCSK9 expression as measured by ELISA (Fig 4E), indicating that for PF-06446846–sensitive proteins, the decrease in reads mapping 3ʹ to the stall site could function as a surrogate measurement of the extent of translation inhibition for a given protein. Thus, PF-06446846 targets should be detectable through differential expression analysis using only the reads that align 3ʹ to the stall site. In our second ribosome profiling study, we found that the magnitude of the buildup of reads at the stall site was smaller for both PCSK9 (Fig 4C) and other targets, while the number of reads mapping 3ʹ to the stall site remained consistent. The variability in magnitude of the stall peak could be an artefact of the size-selection step during library preparation; subsequent to our initial set of experiments, it was reported [25, 26] that ribosome profiling of stalled ribosomes can yield a broader range of protected footprint sizes than the conventional 26–34 nt range [19].

**Fig 5:**
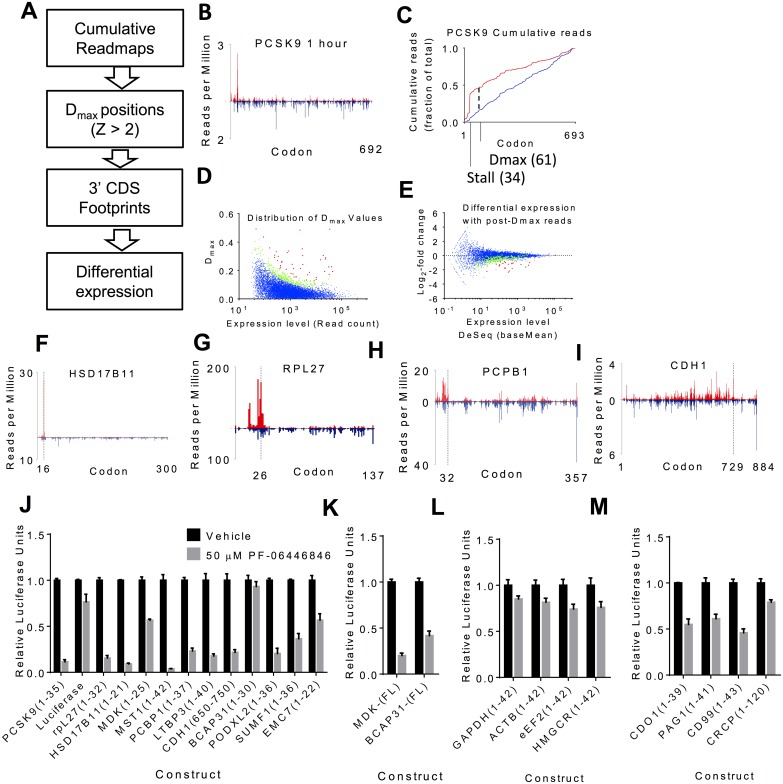
Identification and validation of PF-06446846–sensitive nascent chains. (A) Outline of the approach to identify PF-06446846–targeted mRNAs. (B) Example readplot and (C) example cumulative fractional read (CFR) plot for proprotein convertase subtilisin/kexin type 9 (PCSK9). A CFR plot depicts at each codon the percentage of reads aligning at or 5ʹ to that codon. In all plots, data from 1.5 μM PF-06446846 treatments are shown in red and vehicle treatments are shown in blue. The major stall and the position of D_max_ is marked. (D) Scatterplot showing the distribution of D_max_ values as a function of read counts; red indicates D_max_ Z-score > 3 (see Materials and methods for Z-score calculations) and green indicates 2 < Z-score < 3. (E) Scatterplot of fold change versus expression level when reads mapping 3ʹ to D_max_ position (for Z-score > 2) or codon 50 (for Z-score < 2) are used. Genes for which D_max_ Z-score > 2 and DeSeq fasle discovery rate (FDR) < 10% are highlighted in red, in green for Dmax Z-score > 2 but FDR > 10%, and in purple for D_max_ Z-score < 2 with FDR < 10%. (F–I) Example readplots for PF-06446846–sensitive proteins (F) HSD17B11, (G) RPL27, (H) PCBP1, and (I) cadherin-1 (CDH1). Bars representing the treatment dataset are red and go upwards and bars representing the vehicle datasets are blue and go downwards. All graphs are derived from the 1-h treatment time in the first study. (J) Cell-free translation assays showing inhibition of translation by 50 μM PF-06446846 when the stall sites identified by ribosome profiling are fused to the N-terminus of luciferase. (K) Inhibition of in vitro translation of full-length Midikine- and BCAP31-luciferase fusions in the cell-free translation system. (L) In vitro translation of control constructs not predicted to be inhibited by PF-06446846 from cell-based experiments. (M) In vitro translation of constructs with PF-06446846–induced stalls identified only at the 10-min treatment time. The individual quantitative observations that underlie Fig 5J–M are in S14 Table.

To identify and quantify the sensitivity of individual proteins to PF-06446846, we adopted a computational approach to identify transcripts that could potentially have PF-06446846–induced stalls, estimate the position of the stalls, and use differential expression analysis using only reads aligning 3ʹ to the stall site (Fig 5A). To estimate a 3ʹ bound for the positions of potential PF-06446846–induced pauses, we adapted the approach previously reported [21]. For each gene, we plotted the percentage of the total reads aligning at or 5ʹ to each codon to generate cumulative fractional read (CFR) plots (Fig 5B and 5C). If PF-06446846 induced a stall, the CFR plot should increase rapidly 5ʹ to the stall and level off 3ʹ to the stall. We define the maximum divergence between the PF-06446846 and vehicle CFR plots as the D_max_ and the codon at which D_max_ occurs as the D_max_ position, which is analogous to the Kolmogorov–Smirnov position previously described [21]. For genes with one or more PF-06446846–induced stalls, the D_max_ position occurs 3ʹ to the last stall site. For genes with a high D_max_ (Z-score > 2), we used reads mapping 3ʹ to the position of D_max_ for differential expression analysis (Fig 5D, see Methods for details). Using this approach, we identified 22 PF-06446846–sensitive genes at the 60-min timepoint (Fig 5E–5I, S14 Fig and S13 Table) and 44 genes at the 10-min timepoint (Dmax Z-score > 2, DeSeq FDR > 10%). With the exception of cadherin-1 (CDH1) and interferon gamma–inducible protein 30 IFI30, all PF-06446846–sensitive proteins identified at the 1-h timepoint were also identified at the 10-min timepoint. To test the robustness of our approach, we also analyzed the data from the second ribosome profiling experiment with the same pipeline. Despite most stall peaks being smaller in the second study, we identified all of the same PF-06446846–sensitive sequences as for the data from study 1, demonstrating the advantage of using information from the entire transcript instead of a single codon.

SILAC-based proteomic analyses of lysates of Huh7 cells treated for 4 h or 16 h with 0.25 μM or 1.25 μM PF-06446846 (the same cells as those from which the secretome data were obtained) failed to detect most of the hits identified by ribosome profiling, including PCSK9, when data were analyzed using the same criteria as for the secretome (S9E–S9H Fig). With a reduction in stringency (proteins with 3 unique peptides accepted), a 2- to 3-fold reduction in CDH1 production was detectable after 16 h of treatment with PF-06446846 (S9G and S9H Fig). These results suggest that depletion of the levels of transcript-stalled proteins are likely to occur at a rate governed by protein turnover.

In addition to stalling on the main open reading frame (ORF), it is possible that stalling on upstream ORFs could lead to a change in translation of the main ORF. We thus analyzed all upstream ORFs for a change in read density and found none that displayed an increase in reads with PF-06446846 treatment that was consistent across replicates. SORCS2, FAM13B, and LRP8, the three genes that are downregulated at the 1-h timepoint but do not have a D_max_ Z-score > 2, do not have translated upstream open reading frames (uORFs).

To validate our approach, we tested the translational inhibitory activity of PF-06446846 for a set of the targets identified in the 1-h datasets in HeLa-derived, cell-free translation reactions. In all but two cases, translation of constructs consisting of the predicted stall site fused to luciferase was inhibited by PF-06446846 (Fig 5J). For the other two proteins, Midikine and BCAP31, the translation of luciferase fusions to the full-length proteins was inhibited by PF-06446846 (Fig 5K). Four control sequences predicted not to be PF-06446846–sensitive were inhibited at comparable levels to luciferase alone (Fig 5L).

We next tested the translation inhibition of PF-06446846 towards four example “stall sequences” identified only in the 10-min dataset. PF-06446846 inhibited translation of all of these transcripts only slightly more than luciferase alone (Fig 5M). These results indicate that the most sensitive PF-06446846 targets are those identified using the 1-h treatment time. The additional effects seen in the 10-min treatment could be due to a partial adaptation of the cells on the 1-h timescale, or the cells could be sensitized during the first 10 min of compound treatment because of the sudden media changes required for the experiment. In either case, these data indicate that 1-h treatment times are most appropriate for evaluation of this and similar compounds.

**Fig 6:**
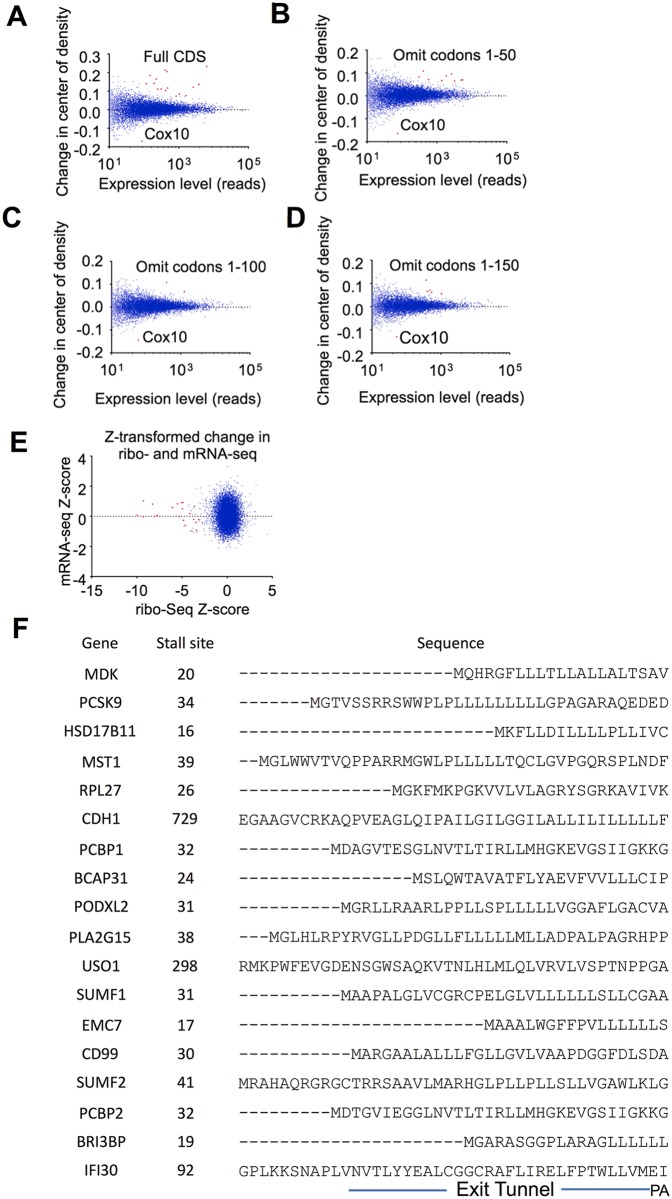
Features of PF-06446846–sensitive transcripts. (A) Changes in the mean read position or center of density [22] plotted against expression levels. Genes with a local Z-score greater than 3 are highlighted in red. (B–D) Center-of-density analysis with first (B) 50, (C) 100, and (D) 150 codons omitted, showing that stalling preferentially occurs before codon 50. (E) Expression changes for PF-06446846–sensitive transcripts occur at the step of translation. Plot of Z-score–transformed [22] read counts for mRNA-seq and ribo-seq. PF-06446846–sensitive nascent chains are highlighted in red. (F) Alignment of PF-06446846–sensitive sequences. The sequences are aligned according to the stall position, and the residues predicted to reside in the ribosome exit tunnel, P-site, and A-site are indicated.

As a complimentary approach, we also calculated the change in mean read position or center of density [22] between the 1.5 μM PF-06446846 and vehicle data and used a Z-score transformation [27] to identify outliers. 17 transcripts with a significant change in the center of read density (Z > 3) were identified in the 1-h datasets (Fig 6A and S14T Fig). Of these, 13 were also identified using the D_max_ approach (S14T Fig). For the hits identified using only center-of-density analysis, VPS25 and TM2D3 had stalls but no decrease in downstream reads, indicating that the stall was unlikely to result in a decrease in protein production (S14P and S14Q Fig). MAPRE1 did not have a clear stall site (S14R Fig). COX10 (S14S Fig) had a series of PF-06446846–induced stalls near the stop codon, which would be difficult to detect using the D_max_ approach because of a lack of downstream reads to quantify. We also used center-of-density analysis to confirm the bias for stalling near the 5ʹ ends of the CDS by repeating the center-of-density analyses omitting the first 50, 100, and 150 codons, respectively (Fig 6B–6D). In all cases, the cluster of outliers disappeared, indicating that while there are exceptions, PF-06446846–induced stalls most often occur in the first 50 codons.

To confirm that the decrease in reads downstream from PF-06446846–induced stalls occur as a result of changes in translation as opposed to mRNA levels, we plotted the change Z-score transformed [22] (see Materials and methods) in mRNA-seq reads versus the changes in ribo-seq reads (Fig 6E). For all identified PF-06446846–sensitive sequences, the changes in ribosome footprints were due solely to changes at the level of translation (Fig 6E). We additionally tested how transcript levels of PF-06446846 targets change with longer-scale treatments and under different growth conditions by treating Huh7 cells grown in either standard or lipoprotein-depleted fetal bovine serum (FBS) for 4 and 24-h with 1.5 μM PF-06446846, then measuring the transcript levels by quantitative reverse transcription PCR (RT-qPCR). With four biological replicates, no consistent changes in target transcript levels were found (S15A and S15B Fig), consistent with PF-06446846–induced changes in protein expression occurring at the translation step.

## Discussion

To our knowledge, PF-06446846 is the first example of an orally-active small molecule that inhibits PCSK9 function through a mechanism that directly reduces its synthesis. Although the narrow margin between PCSK9-lowering and hematopoietic effects likely prevents clinical development of PF-06446846, it demonstrates that it may be possible to modulate PCSK9 function with a small molecule. This has been a challenge for small molecules by conventional means, as it requires the disruption of the small, flat interaction between PCSK9 and the LDLR that represents the primary contact patch at neutral pH. The importance of PCSK9 in regulating LDL-C clearance by LDLR is underscored by the greater degree of LDL-C lowering possible with mAbs relative to statins. However, by their nature, anti-PCSK9 mAbs are unable to modulate PCSK9 and LDL-C levels in an intermediate fashion. Inhibition of PCSK9 synthesis by a small molecule could provide additional options for patients and physicians by offering a range of inhibition to balance safety and efficacy.

This work also represents the first demonstration of the potential for selective inhibitors of eukaryotic mRNA translation as a therapeutic approach. The reduction of PCSK9 demonstrated by PF-06446846 in vivo with no sign of toxicity in the liver is consistent with the high selectivity observed by ribosome profiling. It is unclear whether a lack of complete selectivity evident in the very small number of transcripts whose translation was perturbed by PF-06446846 is responsible for the adverse effects seen outside the liver at the highest dose.

PF-06446846 induces the ribosome to stall on the PCSK9 transcript a few codons beyond to the end of the signal peptide. Ribosome profiling reveals that despite acting on the human ribosome, PF-06446846 is exceptionally specific. Interestingly, the validated off-targets have few common features in their primary structures predicted to be present in the ribosome exit tunnel when stalling occurs (Fig 6F). Although a number of the PF-06446846–sensitive sequences include a leucine repeat or hydrophobic stretch N-terminal to their stall sites, the vast majority of hydrophobic stretches in all of the translated proteins are unaffected. These results indicate that, although PF-06446846 sensitivity is determined by amino acid sequence, stalling cannot be predicted by a simple primary structure motif. Additional requirements for stalling are likely to include the structure adopted by the nascent chain in the exit tunnel [28, 29] or the context in which the sensitive sequence occurs.

One common feature of the PF-06446846–sensitive proteins is that stalling usually occurs early in the protein coding region; 15 of the 18 stalls identified in the 1-h treatment occur before codon 50, with the most N-terminal stall site occurring at codon 16. The heightened sensitivity of N-terminal regions of proteins to PF-06446846 could be due to a unique feature early in the elongation phase of translation. Most stalls would likely occur before the nascent chain emerges from the ribosomal exit tunnel or when only a few residues are extruded. Our mutagenic analysis also indicates that the most critical residues for PF-06446846–induced stalling reside in the exit tunnel, pointing to the ribosomal exit tunnel as a critical element of PF-06446846 action. This is consistent with previous findings that the ribosomal exit tunnel acts as a functional environment, allowing certain peptides and small molecules to induce ribosome stalling [14, 30–35].

Most translation regulation mechanisms in eukaryotes identified to date act on the initiation step [36]. PF-06446846 presents a case of protein production rates controlled during elongation. Translation regulation during elongation presents an interesting mechanistic puzzle as, theoretically, every ribosome that initiates should eventually result in a full-length protein, regardless of how long it takes to traverse the CDS. Our mRNA-seq and RT-qPCR data rule out the possibility that the stalled ribosome induces no-go decay [37] or other mRNA quality control mechanisms that decrease overall mRNA levels. It is possible that PF-06446846–induced stalling near the N-terminus could impede initiation, thus lowering the overall initiation rate. Evidence for “queued” ribosomes (Fig 1H–1J) suggest impeding of initiation could occur for stalls near the N-terminus. However, an impact on translation initiation could not explain the effects of more C-terminal stalling on CDH1 levels (S9G and S9H Fig) and would predict that PF-06446846–sensitive transcripts would display a bias for higher TE, which they do not (S15C Fig). Alternatively, the stalled ribosomes may be removed from the transcript by a “rescue” mechanism and not complete translation. Future experiments will be required to determine the underlying mechanisms of protein reduction because of ribosome stalling by PF-06446846.

While PF-06446846 directly and selectively inhibits the translation of PCSK9 during the elongation phase, most drugs and drug candidates known to modulate human translation target translation initiation factor complexes or upstream signaling pathways such as mammalian target of Rapamycin (mTOR) [38, 39]. Furthermore, they generally modulate translation of a large number of mRNAs. One drug candidate, Ataluren, selectively induces readthrough of premature stop codons while not affecting termination at native stop codons [40]. The present example of PF-06446846 demonstrates that drug-like small molecules have the potential to directly modulate human translation elongation for therapeutic purposes. The variability in the protein primary structures affected by PF-06446846 suggest that each target may be affected by a slightly different mechanism and that analogues of PF-06446846 could be further optimized for improved selectivity for PCSK9 or even to target other proteins.

## Materials and methods

### Ethics statement

All procedures performed on animals in this study were in accordance with established guidelines and regulations and were reviewed and approved by the Pfizer Institutional Animal Care and Use Committee. Pfizer animal care facilities that supported this work are fully accredited by AAALAC International.

### Synthesis of PF-06446846

All chemicals, reagents, and solvents were purchased from commercial sources and used without further purification. All reactions were performed under an atmosphere of nitrogen unless otherwise noted. Nuclear magnetic resonance spectra (^1^H, ^13^C NMR) were recorded with 400 MHz–600 MHz Varian or Bruker spectrometers. Chemical shifts are expressed in parts per million downfield from tetramethylsilane. The peak shapes are denoted as follows: s, singlet; d, doublet; t, triplet; q, quartet; m, multiplet; br s, broad singlet. Because of differences in solvents used in sample preparation and, in some cases, amount of water present, not all exchangeable protons were observable. Mass spectrometry (MS) was performed via atmospheric pressure chemical ionization (APCI) or electron scatter (ESI) ionization sources. Silica gel chromatography was performed using a medium pressure Biotage or ISCO system using columns prepackaged by various commercial vendors including Biotage and ISCO. Whatman precoated silica gel plates (250 μm) were used for analytical TLC. Microanalyses were performed by Quantitative Technologies Inc. and were within 0.4% of the calculated values.

Liquid chromatography mass spectrometry (LCMS) was performed as follows:

#### Analytical UPLC-MS method 1

Column: Waters Acquity HSS T3, C18 2.1 x 50 mm, 1.7 μm; Column T = 60°C. Gradient: Initial conditions: A-95%:B-5%; hold at initial from 0.0–0.1 min; linear ramp to A-5%:B-95% over 0.1–1.0 min; hold at A-5%:B-95% from 1.0–1.1 min; return to initial conditions 1.1–1.5 min. Mobile phase A: 0.1% formic acid in water (v/v). Mobile phase B: 0.1% formic acid in acetonitrile (v/v). Flow rate: 1.25 mL/min

#### Analytical UPLC-MS method 2

Column: Waters Acquity HSS T3, C18 2.1 x 50 mm, 1.7 μm; Column T = 60°C. Gradient: Initial conditions: A-95%:B-5%; hold at initial from 0.0–0.1 min; linear ramp to A-5%:B-95% over 0.1–2.6 min; hold at A-5%:B-95% from 2.6–2.95 min; return to initial conditions 2.95–3.0 min. Mobile phase A: 0.1% formic acid in water (v/v). Mobile phase B: 0.1% formic acid in acetonitrile (v/v). Flow rate: 1.25 mL/min.

High-performance liquid chromatography purity assessment was performed as follows:

#### Analytical HPLC method 1

Column: Waters BEH C8 2.1 x 100 mm, 1.7 μm. Gradient: Initial conditions: A-95%:B-5%; linear ramp to A-0%:B-100% over 0.0–8.20 min; hold at A-0%:B-100% from 8.2–8.7 min; return to initial conditions 8.7–8.8 min; hold at A-95%:B-5% from 8.8–10.3 min. Mobile phase A: 0.2% of 70% perchloric acid in water (v/v). Mobile phase B: acetonitrile. Flow rate: 0.5 mL/min. Detection: UV-210 nm.

#### tert-butyl (3R)-3-[(3-chloropyridin-2-yl)amino]piperidine-1-carboxylate

A mixture of 2-bromo-3-chloropyridine (203.8 g, 1.06 moles), sodium *tert*-amylate (147 g, 1.27 moles), *tert*-butyl (3*R*)-3-aminopiperidine-1-carboxylate (249.5 g, 1.25 moles) in toluene (2 L) was heated to 80°C. To this solution was added chloro(di-2-norbornylphosphino)(2-dimethylaminoferrocen-1-yl) palladium (II) (6.1 g, 10.06 mmol) followed by heating to 105°C and holding for 3 h. The reaction mixture was cooled to room temperature, 1 L of water was added, then the biphasic mixture was filtered through Celite. After layer separation, the organic phase was washed with 1 L of water followed by treatment with 60 g of Darco G-60 at 50°C. The mixture was filtered through Celite and concentrated to a final total volume 450 mL, resulting in the precipitation of solids. To the slurry of solids was added 1 L of heptane. The solids were collected via filtration and then dried to afford the title compound as a dull orange solid (240.9 g, 73% yield). ^1^H NMR (CDCl_3_, 600 MHz) δ 8.03 (d, *J* = 4.7 Hz, 1H), 7.45 (d, *J* = 6.7 Hz, 1H), 6.57–6.51 (m, 1H), 5.08 (br s, 1H), 4.14 (br s, 1H), 3.85–3.30 (m, 4H), 2.00–1.90 (m, 1H), 1.80–1.55 (m, 4H), 1.43 (br s, 9H); ^13^C NMR (CDCl_3_, 100.5 MHz) δ 150.0, 153.2, 146.0, 135.9, 115.3, 112.9, 79.5, 48.6, 46.4, 43.7, 29.8, 28.3, 22.4; UPLC (UPLC-MS method 1): t_R_ = 0.72 min. MS (ES+) 312.0 (M+H)^+^; HRMS (*m/z*) [M+H]^+^ calcd for C_15_H_23_ClN_3_O_2_, 312.1473; found, 312.1467.

#### 4-(3H-[1,2,3]triazolo[4,5-b]pyridin-3-yl)benzoic acid

The following synthesis is based on that reported in [41]. Step 1: To a solution of 2-chloro-3-nitropyridine (95.0 g, 0.600 mol) and ethyl 4-aminobenzoate (99.0 g, 0.600 mol) in toluene (3 L) was added K_2_CO_3_ (166 g, 1.20 mol), BINAP (7.40 g, 11.8 mmol), Pd(OAc)_2_ (2.80 g, 12.5 mmol), and NaI (2.70 g, 18.0 mmol). The mixture was stirred at 110°C for 6 h. The reaction mixture was cooled to 30°C, filtered through Celite, and the filtrate was concentrated in vacuo. The residue was transferred to a separatory funnel with water (300 mL) and extracted with EtOAc (3 x 300 mL). The organic layers were dried over Na_2_SO_4_, filtered, and the filtrate was concentrated in vacuo to give a dark solid residue, which was washed with acetone/water (5/1, 100 mL) and filtered to afford a yellow solid (142 g, 82%).^1^H NMR (DMSO-d_6_) δ 8.60–8.57 (m, 2H), 7.96–7.94 (m, 2H), 7.89–7.87 (m, 2H), 7.12 (dd, 1H), 4.31 (q, 2H), 1.33 (t, 3H). Step 2: To a solution of ethyl 4-((3-nitropyridin-2-yl)amino)benzoate from step 1 (120 g, 0.417 mol), in ethanol (2.00 L), was added Raney-Ni (30 g). The reaction mixture was hydrogenated under a H_2_ atmosphere (50 psi) at 30°C for 20 h. The mixture was filtered through Celite. The filtrate was dried over Na_2_SO_4_, filtered, and the filtrate was concentrated in vacuo to afford a yellow solid. The yellow solid was washed with dichloromethane to give a yellow solid (75 g, 70%). ^1^H NMR (DMSO-d_6_) δ 10.05 (br, 1H), 7.94 (d, 2H), 7.54 (d, 1H), 7.46 (d, 2H), 7.40 (d, 1H), 7.09 (dd, 1H), 4.30 (q, 2H), 1.31 (t, 3H). Step 3: To a solution of ethyl 4-((3-aminopyridin-2-yl)amino)benzoate from step 2 (70.0 g, 0.272 mol) in a mixture of AcOH (70 mL) and water (70 mL) was added NaNO_2_ (23.8 g, 0.345 mol) at 0°C. The mixture was stirred at 30°C for 20 min. The mixture was diluted with dichloromethane (100 mL) and washed with water (3 x 50 mL). The organic phase was dried over Na_2_SO_4_, filtered, and the filtrate was concentrated in vacuo to afford a dark solid. The solid was washed with acetone (30 mL) to afford a white solid (63 g, 86%). ^1^H NMR (DMSO-d_6_) δ 8.91 (d, 1H), 8.91 (dd, 1H), 8.50 (d, 2H), 8.24 (d, 2H), 7.66 (dd, 1H), 4.37 (q, 2H), 1.36 (t, 3H). Step 4: To a solution of ethyl 4-(3*H*-[1,2,3]triazolo[4,5-b]pyridin-3-yl)benzoate from step 3 (60.0 g, 0.224 mol) in MeOH (700 mL) was added 2N NaOH (260 mL, 0.520 mmol). The mixture was stirred at 60°C for 2 h. The mixture was acidified with 1N HCl so that the pH of the solution was approximately pH 1. The reaction mixture was extracted with EtOAc (3 x 100 mL). The combined organic layers were dried over Na_2_SO_4_, filtered, and the filtrate was concentrated in vacuo to afford a white solid (52 g, 97%).^1^H NMR (DMSO-d_6,_ 600 MHz) *δ* 13.23 (br, 1H), 8.91 (d, 1H), 8.76 (d, 1H), 8.48 (d, 2H), 8.24 (d, 2H), 7.67 (dd, 1H); ^13^C NMR (DMSO-d_6_, 100.5 MHz) *δ* 166.5, 151.6, 144.7, 139.2, 137.4, 130.9, 130.1, 129.4, 121.1, 120.5; HRMS (*m/z*) [M+H]^+^ calcd for C_12_H_9_N_4_O_2_, 241.0720; found, 241.0719.

#### N-(3-chloropyridin-2-yl)-N-[(3R)-piperidin-3-yl]-4-(3H-[1,2,3]triazolo[4,5-b]pyridin-3-yl)benzamide (PF-06446846)

Step 1: Oxalyl chloride (8.1 mL, 91.9 mmol) was added to a mixture of 4-(3*H*-[1,2,3]triazolo[4,5-b]pyridin-3-yl)benzoic acid (20.1 g, 83.5 mmol) in dichloromethane (200 mL) and *N*,*N*-dimethylformamide (1 mL). The reaction mixture was stirred at room temperature for 2 h and then evaporated. The residue was diluted with toluene, evaporated to dryness, and dried under vacuum overnight. The crude acid chloride was combined with *tert*-butyl (3*R*)-3-[(3-chloropyridin-2-yl)amino]piperidine-1-carboxylate (26.7 g, 85.5 mmol) in THF (250 mL) and cooled to 0°C. Lithium bis(trimethylsilyl)amide (83 mL, 83 mmol, 1 M solution in THF) was added dropwise. The resulting mixture was warmed to room temperature and stirred overnight. The reaction mixture was diluted with water and extracted with EtOAc (2 × 80 mL). The combined organic layers were dried over MgSO_4_, filtered, and the filtrate was concentrated. The residue was purified by silica gel column chromatography eluting with a gradient of 0%–60% EtOAc/heptane to provide 35.2 g (79%) of the product as a solid. UPLC (UPLC-MS method 2): t_R_ = 0.75 min. MS (ES+): 534.3 (M+H)^+^. Step 2: A solution of *tert*-butyl (3*R*)-3-(4-(3*H*-[1,2,3]triazolo[4,5-b]pyridin-3-yl)-*N*-(3-chloropyridin-2-yl)benzamido)piperidine-1-carboxylate from Step 1 (33.0 g, 61.8 mmol) in dichloromethane (200 mL) and MeOH (40 mL) was treated with HCl (4 M solution in 1,4-dioxane) (309 mL, 1,240 mmol). After stirring at room temperature overnight, the reaction mixture was concentrated. The resulting residue was suspended in a mixture of EtOAc (300 mL)/MeOH (50 mL) and stirred at room temperature overnight. The solids were filtered, slurried in MeOAc overnight, filtered, and dried under vacuum to provide 28.5 g (98%) of crystalline *N*-(3-chloropyridin-2-yl)-*N*-[(3*R*)-piperidin-3-yl]-4-(3*H*-[1,2,3]triazolo[4,5-b]pyridin-3-yl)benzamide hydrochloride. An analytical sample could be recrystallized from MeOH/EtOAc, m.p.: 249–251°C; TLC (CH_2_Cl_2_:MeOH, 80:20, v/v): RF = 0.22; [α]^D^_25_ = +55.9° (c = 1.965, MeOH); ^1^H NMR (d_4_-MeOH_,_ 400 MHz, mixture of rotomers) d8.81 (dd, *J* = 1.6, 4.7 Hz, 1H), 8.62 (br d, *J* = 3.5 Hz, 1H), 8.56 (dd, *J* = 1.2, 8.2 Hz, 1H), 8.33–8.25 (m, 2H), 7.87–7.79 (m, 1H), 7.66–7.55 (m, 3H), 7.42 (dd, *J* = 7.8, 4.7 Hz, 1H), 5.19–5.06 and 4.84–4.70 (m, 1H), 3.89–3.73 (m, 1H), 3.70–3.57 (m, 1H), 3.39 (br d, *J* = 12.5 MHz, 1H), 3.00–2.88 (m, 1H), 2.42–2.37 and 2.20–1.85 (m, 3H), 1.57–1.44 (m, 1H); ^13^C NMR (d_4_-MeOH, 100.5 MHz) d22.9, 26.6, 44.7, 47.7, 52.4, 120.9, 122.0, 126.8, 130.0, 130.9, 132.2, 136.1, 138.9, 139.2, 141.1, 146.1, 149.0, 151.4, 152.4, 170.8; IR (neat): 1658 cm^-1^; HPLC purity (analytical HPLC method 1): 99.52%, t_R_ = 3.048 min; HRMS (*m/z*) [M+H]^+^ calcd for C_22_H_21_ClN_7_O, 434.1491; found, 434.1492; analysis (hydrochloride salt) (% calcd, % found for C_22_H_20_ClN_7_O^.^ HCl^.^ 0.5 MeOH^.^ 0.65 H_2_O): C (54.26, 54.07), H (4.92, 4.65), N (19.68, 19.71), Cl (14.24, 13.98); analysis (freebase) (% calcd, % found for C_22_H_20_ClN_7_O): C (60.90, 60.88), H (4.65, 4.51), N (22.60, 22.44), Cl (8.17, 8.24).

#### Single crystal X-ray analysis for *N-(3-chloropyridin-2-yl)-N-[(3R)-piperidin-3-yl]-4-(3H-[1*,*2*,*3]triazolo[4*,*5-b]pyridin-3-yl)benzamide* PF-06446846 (CCDC 1449348)

Data collection was performed on a Bruker APEX diffractometer at room temperature. Data collection consisted of three omega scans at a low angle and three at a high angle, each with 0.5 step. In addition, two phi scans were collected to improve the quality of the absorption correction. The structure was solved by direct methods using SHELX software suite in the space group P1. The structure was subsequently refined by the full-matrix least squares method. All nonhydrogen atoms were found and refined using anisotropic displacement parameters. The compound crystallizes with two independent molecules in the asymmetric unit in the chiral space group of P1, but exhibits pseudo-inversion symmetry, emulating P-1, that relates the two molecules to each other. Inversion symmetry is broken by the chirality of the molecules, with both molecules having the same stereochemistry.

All hydrogen atoms were placed in calculated positions and were allowed to ride on their carrier atoms. The final refinement included isotropic displacement parameters for all hydrogen atoms. Analysis of the absolute structure using likelihood methods [42] was performed using PLATON [43]. The results indicate that the absolute structure has been correctly assigned. The method calculates that the probability that the structure is correct is 100.0. The Hooft parameter is reported as 0.06 with an esd of 0.019.

The final R-index was 3.8%. A final difference Fourier map revealed no missing or misplaced electron density. Pertinent crystal, data collection, and refinement are summarized in S1 Table. Atomic coordinates, bond lengths, bond angles, torsion angles, and displacement parameters are listed in S2–S7 Tables.

### Cells and reagents

Huh7 cells were obtained from the Japanese Collection of the Research Bioresources Cell Bank and were maintained in RPMI (Life Technologies 11875–093) supplemented with 10% FBS (Sigma F4135), 1% penicillin/streptomycin (Life Technologies 15410–112), and 4 mM Glutamax (Life Technologies 35050–061) at 37°C under a 5% CO_2_ atmosphere. The cells were tested for mycoplasma using the Gen-Probe Mycoplasma Tissue Culture NI (MTC-IN) Rapid Detection method. HeLa cells were obtained from the American Type Culture Collection (ATCC) and grown in CD293 media (Life Technologies 11913–019) supplemented with 10% FBS (Sigma F4135), 1% penicillin/streptomycin (Life Technologies 15410–112), and 4 mM Glutamax (Life Technologies 35050–061) at 37°C under a 5% CO_2_ atmosphere. PF-06446846 was synthesized as described above. Isotopically labelled L-lysine and L-arginine were obtained from Cambridge Isotope laboratories.

### Inhibition of PCSK9 expression in Huh7 cells

3,200 Huh7 cells were seeded in each well of a 96-well plate and allowed to attach and grow overnight. The media was replaced with fresh media containing the indicated concentration of PF-06446846 and 0.5% DMSO vehicle, and the cells were incubated overnight. 50 μL conditioned media was recovered from each well, and PCSK9 levels were measured using the human PCSK9 ELISA assay (Quantikine DPC900) according to manufacturer’s instructions.

### Metabolic labelling

24-well cell culture plates (Bioexpress T-3026) were seeded with 18,000 Huh7 cells per well and returned to the incubator overnight. The media was aspirated and the cells were washed two times with 37°C PBS. 250 μL methionine, cysteine, and glutamine-free RPMI (Sigma R7513) supplemented with 10% dialyzed FBS (Life Technologies 26400036) 2 mM glutamine, 4 mM Glutamax, and 1% Penstrep was added to the cells. The cells were returned to the incubator for 30 min. PF-06446846, cycloheximide, or vehicle was added to the media to the indicated final concentration. The final concentration of DMSO was 0.5%. 27.5 μCi ^35^S-methionine-cysteine cell-labelling mix (PerkinElmer, Inc. NEG772002MC) was added to the cells, and they were returned to the incubator for 30 min. The media was aspirated and the cells were washed once with PBS then lysed by incubation in 50 μL RIPA lysis buffer (25 mM Tris-HCl pH 7.6, 150 mM NaCl, 1% v/v NP-40, 1% w/v sodium deoxycholate, and 1% w/v SDS). 10 μL Lysate was run on an SDS page gel. The gel was dried and exposed to a phosphoimaging screen for 1–4 d. ^35^S-Met/Cys incorporation was measured by quantifying the full lanes using Image-Quant TL.

### SILAC analysis

SILAC was used to facilitate proteomic analysis of the effects of PF-06446846 on the protein population secreted from Huh7 cells. Cells were grown in 10% dialyzed fetal bovine serum in RPMI medium initially lacking L-lysine and L-arginine but supplemented at 0.47 mM (L-arginine) and 0.46 mM (L-lysine) with one of the following combinations: L-lysine and L-arginine of standard isotopic composition (light label); L-arginine:HCl ([^13^C_6_], 99%) and L-lysine:2HCl ([^2^H_4_], 96–98%) (medium label); or L-arginine:HCl ([^13^C_6,_^15^N_4_], 99%) and L-lysine:2HCl ([^13^C_6_,^15^N_2_], 99%) (heavy label). Cells were passaged for five to six doublings with density maintained to keep them actively growing in log phase (30%–90% confluency), after which the incorporation efficiency for the isotopically substituted amino acids was measured as >95%. Cells were next cultured to full confluence to synchronize them in G0/G1 phase. Analysis with propidium bromide showed that 75% of the cells were in G0/G1 phase. The isotopically distinct populations were then replated in fresh medium supplemented with 0.5% dialyzed fetal bovine serum containing the appropriate isotopic forms of L-lysine and L-arginine and allowed to plate down for 2 h before being treated with a test compound or vehicle. The timed experiment was initiated by adding 30 mL of the appropriate medium with 0.5% fetal bovine serum to plated cells as a medium change. Light cells were treated with medium containing vehicle, and the medium and heavy cells were treated with medium containing 0.25 μM and 1.25 μM PF-06446846, respectively. Separate samples of each differentially labeled population were incubated for 4 or 16 h, at the end of which periods the medium was removed and treated with protease/phosphatase inhibitors before being stored at –80°C to await proteomic analysis.

To 28 mL of conditioned medium from each time/dose sample was added 0.28 mL of Halt Protease and Phosphatase Inhibitor Cocktail (100X) (Thermo Fisher Scientific). For each timepoint (1 h, 4 h, and 16 h), 25 mL of medium from each SILAC condition was combined, and the mixed samples were depleted of bovine serum albumin using anti-BSA agarose beads (Sigma). Depletion was performed on 15 mL portions of samples with 5 mL of beads according to the manufacturer's instructions. Flow-through fractions for each timepoint were combined, concentrated to 0.8 mL using 3 kDa MWCO spin columns, treated with 1/20 volume of 20% SDS to give a final SDS concentration of 1%, and then mixed with 0.05 mL of 1 M Tris-HCl, pH 8.5, and 0.2 mL of 4X SDS loading buffer. DTT was added to a concentration of 10 mM and samples were heated to 70°C for 10 min, after which they were allowed to cool to room temperature and treated with 25 mM iodoacetamide for 30 min. A two-thirds portion of each sample (0.7 mL) was loaded to a 1-well 4%–12% NuPAGE Novex Bis-Tris gel (Life Technologies) and run in NuPAGE MOPS SDS running buffer, and the remaining material from each timepoint was fractionated on another gel of the same kind run in NuPAGE MES SDS running buffer to resolve lower-mass proteins. Following staining with Coomassie Blue and destaining, each gel was cut into 15 and 12 horizontal slices, respectively, and each sample was subjected to in-gel digestion with trypsin by standard methods. Digest supernatant samples were collected, gel pieces were extracted with 0.5% formic acid in 50% acetonitrile, and extracts were combined with the corresponding original digests. The samples were desalted using StageTips [44], dried in a centrifugal concentrator, and stored at –20°C.

Peptide mixtures were reconstituted in 0.1% formic acid, and an aliquot of each sample was loaded onto a C18 PicoFrit column (75 μm × 10 cm) (New Objective) coupled to an LTQ Orbitrap Velos mass spectrometer (Thermo Fisher Scientific). Peptides were fractionated using a 2 h linear gradient of acetonitrile in 0.1% formic acid. The instrumental method consisted of a full MS scan followed by data-dependent CID scans of the 20 most intense precursor ions, with dynamic exclusion activated to maximize the number of ions subjected to fragmentation. Peptide identification and relative protein quantification were carried out by searching the mass spectra against a database in which the UniProtHuman and UniProtBovine databases were combined with a database of common contaminant sequences (keratins, trypsin, etc.). Searches were performed using the Mascot search engine [45] (Matrix Science) operating under Proteome Discoverer 1.4 (Thermo Fisher Scientific). The search parameters took into account static modification of S-carboxamidomethylation at Cys, and variable modifications of S-oxidation on Met and stable isotopic substitution (medium and heavy forms) of Lys and Arg. Peptide identifications were made at a 1% false discovery rate. Compound-induced changes in the protein levels were calculated from the peak intensity ratios of unlabeled and isotopically substituted peptides. The ratios of medium-labeled peptides to controls reported effects of 0.25 μM PF-06446846, and the ratios of heavy-labeled peptides to their controls reported effects of 1.25 μM compound. As an important precaution, only sequence-unique peptides were used for these calculations in order to minimize contamination of results for human proteins by peak signals from their bovine orthologs. Specifically, the option "Use Only Unique Peptides" was selected under the Protein Quantification tab in the Workflow Editor of Proteome Discoverer 1.4. In further settings, the options "Replace Missing Quan Values with Minimum Intensity" and "Use Single-Peak Quan Channels" were not selected, and the option to "Reject All Quan Values If Not All Quan Channels Are Present" was activated. The Maximum Allowed Fold Change was set to 15. Further constraints placed on the reporting of SILAC ratios included requiring a minimum of 4 unique peptides per protein, a maximum overall variability of 60% for SILAC ratios, a minimum of 10% sequence coverage, and a minimum of 10 peptide spectral matches per protein. Results for keratins and trypsin were deleted from the output. Protein hit lists (by UniProt accession) were also searched against the UniProt database to identify those designated as "Secreted," and those lacking this designation were designated for present purposes as "Not Secreted." Results exported from Proteome Discoverer were first managed collectively in Microsoft Excel, then exported for graphic viewing using TIBCO Spotfire software.

Proteomic analysis was also performed on the cellular proteomes of the same SILAC-labeled Huh7 cells as those used in the secretome analyses. Each cell pellet was lysed using 0.5 ml of 0.05 M Tris HCl, pH 8.0, 4% glycerol, 1% SDS, 1 mM EDTA, and Halt protease/phosphatase inhibitor cocktail (Thermo Fisher Scientific) at the recommended level. Cell lysates were centrifuged at 13,000 × *g* at 4°C for 10 min, and the protein concentration in each supernatant was measured using the BCA assay (Thermo Fisher Scientific). In separate analyses of cells treated for 4 h and 16 h, equivalent amounts of protein (200 μg) from each of the three SILAC conditions were mixed. Each mixture was concentrated to 70 μl and fractionated by SDS-polyacrylamide gel electrophoresis as described. Gel lanes were cut into 12 horizontal slices and the protein was digested in-gel by standard methods using trypsin. Digests were desalted using C18 StageTips, and peptide identification and ratiometric quantification was performed by liquid chromatography/mass spectrometry (LC-MS/MS) as described above.

### Saturation binding

Saturation binding was conducted to measure total and nonspecific binding of [^3^H] PF-06446846 to purified HeLa 80S ribosomes (purified as described previously [46]). [^3^H] PF-06446846 (21 Ci/mmol, 1 mCi/mL in 100% EtOH) was dried under nitrogen and resuspended in water. Varying concentrations of the tritiated ligand were added to a Costar 3365 96-well plate, alone (total binding), or along with a 200-fold concentration of unlabeled PF-06446846 (nonspecific binding). 0.6 μM of HeLa 80S ribosomes in assay buffer (20 mM HEPES pH 7.6, 100 mM KCl, 5 mM Mg(OAc)_2_, 10 mM NH_4_Cl) were added to the assay plate. The reaction was incubated at room temperature on a rotating platform shaker for 30 min before the reaction mixture was filtered onto a GF/B filter plate (pretreated with 0.3% polyethylenimine) using a PerkinElmer Unifilter 96 Harvester. The plate was allowed to dry overnight before adding 50 μL of Microscint and counting on a Trilux counter. Data were analyzed in GraphPad Prism using the one site-specific binding equation. Specific binding was calculated by subtraction of the nonspecific binding from the total binding at each concentration of [^3^H]-PF-06446846. The K_d_ and B_max_ values were calculated from three independent replicates consisting of 39 specific binding values and are reported along with their 95% confidence intervals. The radioligand [^3^H]-PF-06446846 was prepared from the 6-bromo PF-06446846 precursor, made by the same methods described for the synthesis of PF-06446846 but starting from 5-bromo-2-chloro-3-nitropyridine. Tritium incorporation was accomplished by tritiolysis of the corresponding 6-bromo derivative in methanol/NaHCO_3_ in the presence of 10% Pd/C to afford [^3^H]-PF-06446846.

### Generation of mRNA for cell-free translation

For HeLa-based, cell-free translation assays, PCSK9, PCSK9 truncations and mutations, and off-target stall sites identified by ribosome profiling were cloned as N-terminal fusions to firefly luciferase in plasmid pT7CFE1 (Life Technologies). Translation was driven by an EMCV IRES encoded in the mRNA. All mRNAs used the native translation start site except one PCSK9 encoding mRNA used in Fig 1G and 1H, in which the mRNA encoded an additional 12 residues (MATTHMGSEFAT) encoded as part of the EMCV IRES. The complete EMCV IRES increased the overall translation of this PCSK9 construct, allowing the stalled ribosome trimer peak to be visualized in sucrose gradient profiles.

For rabbit reticulocyte–derived and wheat germ–derived cell-free translation assays, the EMCV IRES was replaced by the rabbit β-globin 5′-untranslated region (5′ UTR). For yeast-derived cell-free translation, the EMCV IRES was replaced by a short synthetic 5′ UTR (5′-GGGAGAAATTAGAATTCAACAC-3′) that included the yeast Kozak sequence (5′- AACACAATGGG-3′; start codon underlined). The yeast Kozak sequence necessitated the mutation of PCSK9 Gly2 to serine. For *E*. *coli*, transcription templates were made using a two-step PCR amplification. PCSK9(1–33)-luciferase was amplified first with primers 5′AGGGGAATTGTATAAGGAGGAAAAAACatgggatcGgaattTgataaacttaagcttgcc3′ and 5′AAACCCCTCCGATTAGAGAGGGGTTATGCTAGttacacggcgatctttccgc3′, then with 5′GCGAATTAATACGACTCACTATAGGGGAATTGTATAAGGAGGAAAAAACa3′ and 5′AAACCCCTCCGATTAGAGAGGGGTTATGCTAGttacacggcgatctttccgc3′. Luciferase was amplified with 5′ACTATAGGGCTTAAGTATAAGGAGGAAAAAATATGGAAGACGCCAAAAACATAAAGAAAG3′ and 5′AAACCCCTCCGATTAGAGAGGGGTTATGCTAGttacacggcgatctttccgc3′ followed by 5′GCGAATTAATACGACTCACTATAGGGCTTAAGTATAAGGAGGAAAAAAT3′ and 5′AAACCCCTCCGATTAGAGAGGGGTTATGCTAGttacacggcgatctttccgc3′. For *E*. *coli* cell-free translation, transcription was carried out using the PCR product.

For in vitro transcription, vectors were linearized with PmeI (NEB Cat # 0560L) then purified with Zymo DNA clean and concentrator kit. Reactions were set up with 5 mM each NTP (Sigma) (pH adjusted to 7), 30 mM Tris-CL pH 8.1, 25 mM MgCl_2_, 0.01% Triton X-100, 10 mM DTT, 1U/mL pyrophosphatase (Sigma), 50 ng/μL DNA, and 0.1 mg/mL T7 RNA. Reactions were incubated for 1–4 h at 37°C. DNaseI (Promega) was added to a final concentration of 0.1 U/μL, and the reactions were incubated an additional 15 min at 37°C. RNA was purified using the Zymo Research RNA clean and concentrator kit. For yeast lysate, capped mRNA was synthesized using modified reaction conditions containing 2 mM ATP, UTP, and CTP; 0.2 mM GTP; 2 mM Ribo m^7^G Cap Analog (Promga P1712); 10 mM DTT; 1.2 U/μL RNAsin Plus (Promega); 50–100 ng/μL DNA template; and 0.1 mg/mL T7 RNA polymerase. For wheat germ translation, the mRNA was capped using the Vaccina capping kit (NEB) and tailed using Poly-A polymerase (NEB) using the manufacturer’s recommended conditions.

### HeLa-derived cell-free translation system

Translationally active HeLa lysates were prepared as described previously [47]. HeLa cell paste was thawed and resuspended in an equal volume of hypotonic lysis buffer (20 mM HEPES pH 7.5, 10 mM potassium acetate, 1.8 mM magnesium acetate, and 1 mM DTT), and cells were incubated on ice for 10 min then lysed in a Dounce homogenizer. One-fourteenth of the total volume of a solution consisting of 20 mM HEPES-pH 7.5, 997.5 mM potassium acetate, 1.15 mM potassium chloride, 1.8 mM magnesium acetate, and 1 mM DTT was added to bring the salt and buffer composition in the lysate to 20 mM HEPES-KOH pH 7.5, 76.5 mM potassium acetate, 76.5 mM potassium chloride, 1.8 mM magnesium acetate, and 1 mM DTT. The lysate was centrifuged twice at 1,200 x g for 5 min, and the supernatant was recovered each time. Supernatants were aliquoted, flash-frozen in liquid nitrogen, and stored at -80°C. Lysates were freeze-thawed only once, as a significant loss of translational activity was observed with each freeze–thaw cycle.

A 50 μL translation reaction was set up by combining 25 μL translationally active lysate, 4.5 μL mix 1, 5 μL mix 2, 1 μL RNasin Plus (Promega), 1 μg RNA template and 5 μL PF-06446846 solution in 5% DMSO or 5% DMSO control. Mix 1 consisted of 17.7 mM magnesium acetate, 1.24 M potassium acetate, 300 mM HEPES-KOH pH 7.5, and 50 mM DTT. Mix 2 consisted of 12.6 mM ATP, 1.2 mM GTP, 200 mM creatine phosphate (Sigma), 0.6 mg/mL creatine kinase (Sigma), 0.92 mg/mL bovine tRNA (Sigma), and 1.7X MEM essential amino acids, 3.4X MEM nonessential amino acids, and 0.7 mM glutamine (Life Technologies). Total reaction volumes were scaled based on the needs of downstream analyses, with 10 μL reactions typically being used for luciferase assays and 50μL reactions for toeprinting. Reactions were incubated at 30°C for 45 min for full-length, PCSK9-encoding mRNAs. Other validation mRNAs were found to be more translationally active in the HeLa cell-free systems and were incubated for only 15 min. For luciferase assays, 5–10 μL of the translation mixture was combined with 50 mL Steady-Glo luciferase substrate (Promega), and luminescence was measured using a Veritas microplate luminometer (Turner Biosystems).

### Yeast-derived cell-free translation systems

Translationally active yeast lysates were prepared as described previously [48]. The L-A virus-cured yeast strain, YWG3 [48], was grown in YPD at 30°C to an OD_600_ of 3–5, then chilled on ice. Cells were pelleted by centrifugation at 4,000 rpm 3,300 x g in a Beckman Coulter J6-M1 centrifuge for 10 min with a JS-4.2 rotor. Cells were washed by resuspending and pelleting four times in an ice-cold buffer containing 30 mM HEPES-KOH pH 7.4, 100 mM potassium acetate, 2 mM magnesium acetate, and 8.5% w/v mannitol. Pellets were stored at –80°C until needed. For preparation of translationally active lysates, pellets were resuspended in an equal volume of lysis buffer (30 mM HEPES-KOH pH 7.4, 100 mM potassium acetate, 2 mM magnesium acetate, 8.5% w/v mannitol, 2 mM DTT, and 0.5 mM phenylmethanesulfonyl fluoride). The cell-buffer slurry was combined with 0.5 mm diameter glass beads in a round-bottom tube, and yeast were lysed by manually shaking the tube in a vertical motion of 50 cm, 2 times per second for 3 1-min sessions with 1 min of chilling on ice in between. The lysate was recovered with a Pasteur pipette and centrifuged at 30,000 x g for 15 min, after which supernatants were retained. It was found that at this stage they could be aliquoted, flash-frozen, and stored at –80°C with minimal loss of activity. The cleared lysates were then buffer exchanged into 30 mM HEPES-KOH pH 7.4, 100 mM potassium acetate, 2 mM magnesium acetate, and 2 mM DTT using a 10-mL G-25 spin column (Zeba). Lysates were aliquoted, flash frozen in liquid nitrogen, and stored at –80°C.

For translation reactions, individual aliquots were thawed once. Calcium acetate was added to a final concentration of 1 mM, micrococcal nuclease (Sigma) was added to a final concentration of 10 μg/mL, and the lysates were incubated at room temperature for 10 min to degrade endogenous mRNA. EGTA was added to a final concentration of 9.4 mM to inactivate the micrococcal nuclease. For a typical translation reaction, 6.3 μL lysate was combined with 1 μL Mix 1 (220 mM HEPES-KOH pH 7.4, 1.2 M potassium acetate, 15 mM magnesium acetate, and 17 mM DTT), 1 μL Mix 2 (30 mM HEPES-KOH pH 7.4, 7.5 mM ATP, 1 mM GTP, 250 mM creatine phosphate, 3.3 mg/mL creatine phosphokinase, 2X MEM essential amino acids, 4.1X MEM nonessential amino acids, and 0.85 mM glutamate [Life Technologies]), 0.34 μL RNAsin Plus (Promega), 0.33 μL capped mRNA, 1 μL PF-06446846, or vehicle at 10 times the final concentration and water to 10 μL. Reactions were incubated 1 h at room temperature, and luciferase activity was measured using the Steady-Glo luciferase assays system (Promega).

### Rabbit reticulocyte lysate cell-free translation

Rabbit reticulocyte lysate cell-free translation systems were obtained from Promega and used according to the manufacturer’s instructions. 10 μL reactions were programmed with 0.2 μg noncapped mRNA and 50 μM PF-06446846 or vehicle. Reactions were incubated at 30°C for 45 min and luciferase activity was measured using the Steady-Glo luciferase assay system (Promega).

### Wheat germ cell-free translation

Wheat germ cell-free translation kits were obtained from Promega and used according to the manufacturer’s instructions, except that the mRNA was not denatured at 67°C prior to translation reactions. 10 μL reactions were programmed with 0.5 μg capped and tailed mRNA and reactions were incubated at room temperature for 60 min. Luciferase activity was measured using the Steady-Glo luciferase assay system (Promega).

### *E*. *coli* cell-free translation

Translationally active cell extracts were prepared from *E*. *coli* MRE-600 as described previously [49]. 5 μL reactions were programmed with 0.34 μg mRNA and 100 μM PF-06446846 or vehicle. Reactions were incubated at 30°C for 30 min, and luciferase activity was measured using the Steady-Glo reagent (Promega).

### Ribosomal toeprinting

For ribosomal toeprinting, a 50 μL HeLa-based, cell-free translation reaction was carried out in the presence of PF-06446846 or vehicle according to the above protocol. Translation reactions were transferred to ice and supplemented with 0.5 mM each dNTP, 50 mM Tris-Cl pH 8.3, 9.4 mM MgCl_2_, 10 mM DTT, 0.8 U/μL RNasin Plus (Promega), and 0.1 mg/mL cycloheximide. The mix was incubated at 55°C for 2 min. This step was found to be necessary and had previously been determined to be necessary for the successful detection of ribosomal toeprints from *Saccharomyces cerevisiae* and *Neurospora crassa* extracts [50]. The reactions were cooled to room temperature, and 5′-FAM-labelled primers (IDT) were added to a final concentration of 2 μM and AMV reverse transcriptase (Promega) was added to a final concentration of 0.5 U/μL. The primer used for full-length PCSK9 constructs was 5′-ATCTTGGTGAGGTATCCCCG-3′. For truncated constructs, a primer annealing to the luciferase gene with the sequence 5′-GATATGTGCATCTGTAAAAG-3′ was used. The total volume of each reverse transcription reaction was 100 μL. The reactions were incubated at 37°C for 45 min, then 70°C for 5 min. 20 μL 0.5 M EDTA and 20 μL 1 M NaOH were added and the reactions were incubated at 65°C for a further 15 min. 200 μL nuclease-free water was added to the reactions, and bulk proteins were removed using two extractions with basic phenol–chloroform. Reverse transcription products were purified and concentrated from the aqueous phase using the DNA clean and concentrator-5 Kit (Zymo Research). HiDi Formamide was added to a final concentration of at least 50%, and samples were subject to capillary electrophoresis on an Applied Biosystem 3730XL DNA Analyzer along with the Genescan 500 LIZ-labelled size standards (Life Technologies). DNA fragment lengths were determined using the Peak Scanner 2 software (Agilent Technologies).

To correct for small changes in fragment mobility because of the FAM tag and the specific sequence, ddGTP sequencing lanes were generated using the parent vectors, the FAM-labelled primers, and the Sequenase V2 DNA sequencing kit (USB) following manufacturer’s instructions, except that in the labeling reaction, 0.65 μM dATP was substituted for α-^32^P-dATP. The sequencing products were cleaned using the Zymo-5 DNA clean and concentrator kit (Zymo Research) and run alongside the toeprinting products. The observed differences between the apparent lengths based on the commercial size standards and the ddGTP termination products in the region of interest were between three and four nucleotides. These differences were taken into account in the reported toeprint positions.

### Sucrose density separation of stalled products

In vitro translation reactions (50 μL) were carried out with 100 μM PF-06446846 using the Pierce human (Hela cell) in vitro translation kit and programmed with 2 μg of mRNA encoding the full-length PCSK9 coding sequence plus 12 N-terminal, vector-derived residues. To radiolabel the products, reactions were supplemented with 10 μCi of ^35^S-methionine (PerkinElmer NEG709A). Reactions were incubated at 30°C for 45 min and then placed on ice. 40 μL of each was diluted with 60 μL of buffer (10 mM Tris-Cl pH 7.5, 100 mM potassium chloride, 5 mM magnesium chloride, and 1 mM DTT) and loaded onto a 10%–50% (w/v) sucrose density gradient made in the same buffer. Gradients were centrifuged at 50,000 rpm (237,000 x g) in a Beckman Coulter SW55Ti rotor for 1.25 h at 4°C and then fractionated on an ISCO Inc. gradient fractionator fitted with a UA-6 absorbance cell. Absorbance traces at 254 nm were recorded, and 24 fractions of approximately 200 μL each were recovered. Adjacent fractions from the polysome region of the sucrose density gradient were pooled to generate four pools of two fractions. 7.2 μL of 10 mg/mL oyster glycogen was added to each followed by 1 mL ethanol. After overnight incubation at –20°C, samples were pelleted and resuspended in 10 μL of water containing 0.25 units of benzonase (Novagen) and then prepared for electrophoresis on a Novex 10%–20% polyacrylamide tricine SDS gel according to the manufacturer’s instructions. A lane of Novex SeeBlue2 prestained protein markers was run on the same gel for molecular weight estimation. The gel was subsequently rinsed in water on a shaker twice for 10 min, then dried, exposed to a phosphor screen, and imaged on a GE Typhoon scanner.

### In vivo activity and safety evaluation of PF-06446848

Male Sprague-Dawley (Crl:CD [SD] rats, five per group; 6–8 wk old at initiation of dosing) were administered PF-06446846 (Lot No. PF-06446846-01-0003, 92.2%) at 5, 15, or 50 mg/kg/day in a vehicle of 200 mM citrate buffer in 0.5% methylcellulose (w/v) by oral gavage. A control group of five animals received 200 mM citrate buffer in 0.5% methylcellulose (w/v) by oral gavage. The dose volume for all animals was 5 mL/kg/day based on the most recent individual body weight. All animals were observed daily during the pretreatment period, predose, at approximately 1 and 4 h postdose, at the end of the day, and once on the day of necropsy. All animals were weighed pretreatment, prior to dosing on days 1, 3, 7, 10, 14, and at termination (fasted on day 15). Individual food consumption for all animals was recorded on days 7, 10, and 14. Other measurements and analyses from the day 15 terminal sample collection included hematology, coagulation, clinical chemistry, and immunophenotyping. Plasma PF-06446846 and PCSK9 concentrations were measured from all animals on days 1 and 12 at approximately 1, 3, 6, and 24 h postdose. At the end of the treatment period, animals were humanely killed and necropsied. After necropsy, gross organ examination was performed; the liver was weighed, and a selected set of tissues was collected, processed, and examined microscopically.

### Hematology and clinical chemistry measurements

Hematology endpoints including red blood cells, red cell distribution width, hemoglobin, reticulocytes, hematocrit, platelets, mean cell volume, mean platelet volume, mean cell hemoglobin, white blood cells, mean cell hemoglobin concentration, and white cell differential were determined using the Siemens Advia 120/2120 Hematology platform following manufacturer’s protocols. The clinical chemistry panel included measurements of alanine aminotransferase, gamma glutamyltransferase, albumin, globulin, glucose, alkaline phosphatase, high-density lipoprotein cholesterol, aspartate aminotransferase, total protein, low density lipoprotein cholesterol, blood urea nitrogen, phosphorous, calcium, potassium, chloride, sodium, total cholesterol, and creatine. The panel was performed using Siemens Advia 1800 Clinical Chemistry Platform following manufacturer’s protocols.

### Measurement of plasma PCSK9 concentration

A CircuLex Mouse/Rat PCSK9 ELISA Kit (Cat# CY-8078) was used to measure PCSK9 levels in the plasma. Briefly, plasma samples were diluted 1:200 in sample buffer and 100 mL of sample in duplicate was added to the supplied microplate (coated with anti-PCSK9 polyclonal antibody). A mouse PCSK9 standard sample was serially diluted 2-fold and also added in duplicate. The plate was incubated at room temperature for 1 h before washing with wash buffer. A detection reagent containing anti-PCSK9 polyclonal antibody conjugated to horseradish peroxidase was then added to the plate and incubated for 1 h at room temperature. After washing, a chromogenic substrate tetra-methylbenzidine (TMB) was added for 15 min before adding a stop reagent. Absorbance was measured using a spectrophotometric microplate reader at dual wavelengths of 450/540 nm, and data were converted to ng/mL using the mouse PCSK9 standard.

### Measurement of plasma PF-06446846 concentration

A LC-MS/MS assay was used to determine PF-06446846 concentrations in rat plasma. PF-06446846 plasma standards were prepared over the concentration range of 1 ng/mL to 2,500 ng/mL. Proteins were precipitated from 25-μL aliquots of matrix blanks, standards, and plasma study samples by the addition of 200 μL of 4 ng/mL of terfenadine in acetonitrile as an internal standard. Following mixing and centrifugation, the final sample extracts were prepared by diluting 120 μL of supernatant with 240 μL of water. Extracts were separated chromatographically using a 4-min linear gradient of acetonitrile and 0.1% formic acid in water on a Phenomenex (Torrance, CA), Kinetix C18 2.1 mm x 30 mm, 2.6 μm particle chromatography column. Analytes were ionized by electrospray in the positive ion mode and detected using a Sciex (Toronto) API-5500 triple quadrupole mass spectrometer in the multiple reaction monitoring (MRM) mode. Mass-to-charge transitions were *m/z* 434.2 to 167.1 for PF-06446848 and 472.0 to 436.0 for terfenadine. Calibration curves were constructed by the weighted linear regression (1/x^2^) of the chromatographic peak area ratios (analyte/internal standard) versus standard concentration. Unknown sample extracts were quantified against the standard curve based on the analyte/internal standard peak area ratios.

### Ribosome profiling

All python scripts used for processing ribosome profiling data are included in S1 Text. Huh7 cells were grown in 10-cm plates to a confluence of 50%–80%. The cells were always passaged into fresh media within 24 h of the experiment. The media was replaced with fresh, prewarmed media containing 1.5 μM or 0.3 μM PF-06446846 or vehicle (0.5% DMSO), and the plates were immediately returned to the incubator. Because the effects of small molecules such as harringtonine [19], lactimidomycin [51], and cycloheximide that act directly on the ribosome are observable in ribosome profiling experiments with treatment times of a few minutes, we chose treatment times of 10 and 60 min for these studies. Treatment time was measured from the time that the fresh PF-06446846–containing media was added to the cells to the time it was removed. The cells were harvested by aspiration of media and immediate addition of ice-cold PBS containing 100 μg/mL cycloheximide. The PBS plus cycloheximide was aspirated with a vacuum aspirator and 400 μL lysis buffer (20 mM Tris-Cl pH 7.4, 150 mM NaCl, 5 mM MgCl_2_, 1 mM dithiothreitol, 100 μg/mL cycloheximide, 1% Triton X-100, and 25 U/mL DNase I [Promega]) was added. Cells were lysed, ribosome footprints were generated and purified, and deep sequencing libraries were prepared as previously described [24]. For mRNA-seq libraries, total RNA was extracted from lysates using Trizol (Thermo Fisher Scientific Cat # 15596–026), and poly-A-tailed RNA was isolated using Trizol reagent according to manufacturer’s instructions. Poly-A mRNA was fragmented using (Thermo Fisher Scientific Cat # 61002) according to manufacturer’s instructions. The 26–34 nt fragment range was selected by UREA-PAGE, and libraries were prepared using the same protocol as for ribosome footprints. Barcoded libraries were multiplexed and sequenced using an Illumina HiSeq2000 (QB3 Vincent J. Coates Genomics Sequencing Laboratory, University of California, Berkeley).

### Ribosome profiling data processing

De-multiplexed reads were stripped of 3′ cloning adapters using the Fast-X toolkit (http://hannonlab.cshl.edu/fastx_toolkit/). Reads were aligned to the human ribosomal RNA sequences using Bowtie [52] and ribosomal reads were removed. A reference transcriptome was generated by downloading the CDS, 5′ UTR, and 3′ UTR regions of known protein-coding transcripts from Ensembl’s Biomart (www.ensembl.org/biomart) and combined into single transcript sequences using a custom python script. The database selected in Ensembl Biomart was “Ensembl genes 78” and the dataset selected was “Human genes (GRCh38.p7).” For analyses, we experimentally determined the major splice isoform for each expressed protein-coding gene by first aligning the pooled mRNA-seq reads to a reference transcriptome consisting of all transcripts marked as “KNOWN” and “protein-coding” in ENSEMBL using Kallisto v 0.42.5 [53] with options “—single -l 28 -s 1”. From the Kallisto output, a list of transcripts consisting of the most abundant splice isoform from each gene that had a transcripts-per-million (TPM) value of 1 or greater was extracted.

For the ribosome profiling analyses, nonribosomal reads were aligned to a reference transcriptome consisting of the most abundant splice isoform with a TPM of 1 or more for each gene using Bowtie [52] with an alignment seed length of 23 and output options “-a,” “—best,” and “—strata,” which instructs Bowtie to report all equal best alignments. In order to eliminate reads mapping to repetitive, low-complexity regions while also allowing the analysis of genes with close paralogs, we used only the best alignment for each individual read and considered reads with alignments of the best quality to five or fewer sites in the reference transcriptome. In parallel, using a reference transcriptome consisting of all transcripts that are at least 10% of the isoforms from a given gene revealed no additional PF-06446846 targets.

Custom python scripts were used to generate readmaps consisting of the number of footprints mapping to each nucleotide and codon of each CDS based on the inferred P-site position. P-site positions were assigned based on a 14-nt offset of the 5′ end of the alignment [18]. We found that for our data, a 14-nt offset placed the first major peak of ribosome footprint data at nucleotide position +2 relative to the start and –5 relative to the stop. To generate the ribosomal footprint density plots for Figs 4 and 5 and S13 Fig, the number of ribosomal footprints aligning to each position was divided by the total number of reads aligning to the protein-coding regions, then multiplied by 1,000,000 to yield reads per million. All read density plots represent average values for the biological replicates.

Metagene analysis of the global ribosome footprint distribution was carried out by calculating the normalized mean read density at each codon relative to the start and stop positions as described previously [20]. Briefly, the number of reads mapping to a given position on a CDS was divided by the average read density for that CDS. These values were then averaged across all transcripts of sufficient length. For these analyses, only the annotated transcript with the longest CDS for each gene was considered.

### Z-score transformations

Z-score transformations [22, 54] were used to identify outlier values for log_2_-fold changes in TE, changes in centers of density, and D_max_. In all cases, genes were grouped by expression level into bins of 300 genes, and a standard deviation for the value of interest was calculated for each gene. For TE changes, center-of-density changes, and D_max_ values, the Z-score for a given gene is the number of standard deviations the value is from the mean, based on mean and standard deviation of the value for other genes in the same bin. This procedure allows values for TE, center of density, and D_max_ to take into account the increased variability for genes with lower read count values. A full description of the procedure applied to ribosome profiling data used is described in [22].

### Identification of additional PF-06446846–sensitive proteins

For each transcript with sufficient read density, we plotted the cumulative percentage of total footprints aligning to each codon using the combined biological replicates from 1.5 μM PF-06446846–treated cells and vehicle-treated cells to generate cumulative fractional read (CFR) plots. CFR curves increase rapidly 5′ to a stall site and level off 3′ to a stall site. If a PF-06446846–induced stall is present, the codon at which the PF-06446846 and vehicle datasets are maximally divergent occurs 3′ to the last stall. We define the maximum divergence between the drug and vehicle CFR plots as the D_max_ and the codon at which D_max_ occurs as the D_max_ position. To select a minimum value of D_max_ for our analyses, we examined the distribution of D_max_ values obtained when a D_max_ is calculated for each gene using two vehicle datasets and the distribution obtained from a single 1.5 μM PF-06446846 dataset and a single vehicle dataset (S12 Fig). Because we use D_max_ only to choose a 5′ boundary for differential expression analysis, we chose a minimum D_max_ Z-score of 2, knowing that most genes that fall above this minimum will do so by chance (S12 Fig). For transcripts with a maximum D_max_ Z-score of 2 or higher, footprints mapping 3′ to the maximum D_max_ site were extracted for DeSeq analysis. Reads mapping 3′ to codon 50 were extracted for DeSeq analysis for all other genes. Decreases were considered significant if they resulted in a DeSeq false discovery rate < 0.1. Precise stall locations were determined by manually examining the read density plots. Results of the analyses above are included in S13 Table.

### Center-of-density analyses

Center-of-density analyses were done as previously described [22], except that the mean read position was used instead of the median. For each transcript, the average position of all the alignments was calculated. Changes in the average position between the PF-06446846 and the vehicle treatments were calculated and subjected to a Z-score transformation to identify outliers relative to the local expression level bin. Transcripts with a center-of-density Z-score of 3 or higher were manually examined for PF-06446846–induced stalls.

### Measurement of transcript levels by RT-qPCR

9,600 Huh7 cells were seeded into each well of a 96-well plate and returned to the incubator overnight. 1.5 μM PF-06446846 or vehicle (0.5% DMSO) was added to the cells, and the cells were returned to the incubator for 4 or 24 h. Lysates were harvested using Ambion’s cell-to-Ct kit (AM1729) following the manufacturer’s instructions. RT-qPCR was done using Life Technologies FastVirus kit (Cat # 4444432) on a Thermo Fisher Scientific Quantstudio 3 system. The Taqman probes (Thermo Fisher Scientific, FAM-labelled) used were PCSK9; HS00545399_m1, RPL27; Hs03044961_g1, HSD17B11; Hs00212226_m1, MST1; Hs00360684_m1, PCBP1/2; Hs01590472_mH, CDH1; Hs01023894_m1, Transferrin; and Hs01067777_m1. The reference probe was VIC-labelled and targeted PPIA Hs99999904_m1.

### Data availability

The mass spectrometric proteomics data have been deposited to the ProteomeXchange Consortium via the PRIDE [55] partner repository with the dataset identifiers PXD005822 (secretome analyses) and PXD005835 (analyses of cell lysates). The ribosome profiling and accompanying RNA-seq datasets have been deposited to the NCBI Gene Expression Omnibus with accession number GSE94454. The atomic coordinates of PF-06446846 have been deposited to the Cambridge Crystallographic Data Center (CCDC) with accession number 1149348. The individual quantitative observations that underlie Figs 1B–D, 2, 4E, 5J–M, S9A–D, S10B–F, S11, and S15A–B are in S14 Table.

## Supporting information

S1 Fig PF-06446846 specifically inhibits PCSK9 translation. (A) ELISA showing PCSK9 levels in conditioned media from Huh7 cells incubated overnight in the indicated concentrations of PF-06446846. Note that the concentrations of PF-06446846 increase in five-fold increments. (B) Representative SDS-PAGE of total lysates of Huh7 cells labelled with ^35^S Met/Cys in the presence of varying concentrations of PF-06446846. Cycloheximide (CHX) was used as a control. (C) Quantitation ^35^S incorporation by densitometry, error bars represent one standard deviation of three replicates. (D) Translation inhibition curves for PCSK9(1–35)-luciferase (grey circles) and luciferase alone (black squares) in HeLa-derived cell-free translation reactions. (E) Nucleotide and amino acid sequences of constructs shown in Fig 1C.
(TIFF)

S2 Fig ^1^H NMR (CDCl_3_, 400 MHz) spectrum of *tert-butyl (3R)-3-[(3-chloropyridin-2-yl)amino]piperidine-1-carboxylate*. (TIFF)

S3 Fig ^13^C NMR (CDCl_3_, 100.5 MHz) spectrum of *tert-butyl (3R)-3-[(3-chloropyridin-2-yl)amino]piperidine-1-carboxylate*. (TIFF)

S4 Fig ^1^H NMR (d_6_-DMSO, 600 MHz) spectrum of *4-(3H-[1*,*2*,*3]triazolo[4*,*5-b]pyridin-3-yl)benzoic acid*. (TIFF)

S5 Fig ^13^C NMR (d_6_-DMSO, 100.5 MHz) spectrum of *4-(3H-[1*,*2*,*3]triazolo[4*,*5-b]pyridin-3-yl)benzoic acid*. (TIFF)

S6 Fig ^1^H NMR (d_4_-MeOH, 400 MHz) spectrum of *N-(3-chloropyridin-2-yl)-N-[(3R)-piperidin-3-yl]-4-(3H-[1*,*2*,*3]triazolo[4*,*5-b]pyridin-3-yl)benzamide* PF-06446846. (TIFF)

S7 Fig ^13^C NMR (d_4_-MeOH, 100 MHz) Spectrum of *N-(3-chloropyridin-2-yl)-N-[(3R)-piperidin-3-yl]-4-(3H-[1*,*2*,*3]triazolo[4*,*5-b]pyridin-3-yl)benzamide* PF-06446846. (TIFF)

S8 Fig Single crystal X-ray analysis for *N-(3-chloropyridin-2-yl)-N-[(3R)-piperidin-3-yl]-4-(3H-[1*,*2*,*3]triazolo[4*,*5-b]pyridin-3-yl)benzamide* PF-06446846 (CCDC 1449348) ORTEP with ellipsoids drawn at 50% confidence level. (TIFF)

S9 Fig PF-06446846 effects on global protein synthesis and secretion. (A-D), Stable isotope labelling by amino acids in cell culture (SILAC) analysis of Huh7 cellular secretome after (A,B) 4-hour and (C,D) 16-hour treatment with 0.25 μM (A,C) or 1.25 μM (B,D) PF-06446846. (E-H), SILAC analysis of the cellular fraction from the experiments shown in panels a-d respectively. Ribosome profiling hits that are detected in the SILAC data are highlighted in red and labelled.
(TIFF)

S10 Fig PF-06446846-sensitivity of PCSK9 mutations. (A) General schematic of mRNA constructs used to program cell-free translation reactions. Sites of mutations are in red and spacer residues are in orange. (B) Constructs, sequences and PF-06446846 sensitivity of PCSK9 mutants. Sequences are colored as in a, with the vector encoded residues in grey for the luciferase construct. Note that PCSK9 residues 34 are 35 are the same as luciferase resides 2 and 3. All error bars represent one standard deviation of three replicates. (C-F), Inhibitory activity of PF-06446846 in (C) Rabbit Reticulocyte Lysate (RRL) (D) Wheat Germ (E) Yeast and (F) *E*. *coli* cell-free translation systems. Relative luciferase activities of cell-free translation reactions programmed with PCSK9(1–35)-luciferase in the presence of vehicle (Grey bars) or 50 μM PF’846 panels (C-E) or 100 μM PF-06446846 in (F). All error bars represent one standard deviation of three replicates. The individual quantitative observations that underlie Fig. S10B-F are in supplementary data S14 Table.
(TIFF)

S11 Fig Liver transaminase and albumin levels. (A) Alanine transaminase (ALT), (B) Aspartate aminotransferase (AST), and (C) Albumin levels in rats measured 24-hours following 14 daily oral doses of PF-06446846. Bars represent group mean + standard deviation. Symbols represent individual animal values. The middle horizontal bar represents the group mean +/- standard deviation. Difference between group means relative to vehicle was performed by a 1-way ANOVA followed by a Dunnett’s multiple comparisons test; * p< = 0.05. The individual quantitative observations that underlie Fig. S11 are in supplementary data S14 Table.
(TIFF)

S12 Fig Distribution of reads relative to start and stop codons. (A) 10 minute datasets (top panel, replicate 1, middle panel; replicate 2, bottom panel replicate 3, left 1.5 μM PF-06446846 treatment, right vehicle treatment). (B) 1 hour treatment, panels arranged as in (A). (C) Riboseq datasets from second experiment. (top panel; replicate 1, bottom panel; replicate 2, Left panels; 1.5 μM PF-06446846 treatment, Right panels; vehicle treatment. (D) mRNA-Seq datasets from second treatments. Panels are arranged as in (C).
(TIFF)

S13 Fig Reproducibility of read count data; Scatterplots comparing raw read counts between replicates for (A) 10-minute treatment times (B) 1-hour treatment times and (C) the second ribosome profiling experiment. (TIFF)

S14 Fig PF-06446846-sensitive nascent chains. (A-O), (With Fig 5F–5I) Readplots for PF-06446846-sensitive proteins identified using the “D_max_” approach. (P-S), Additional genes displaying a change in the center of density. (P), VPS25, (Q), TM2D3, and (R), MAPRE1. (S) The Cox10 stall occurs near the stop codon. (T) PF-06446846-sensitive sequences identified in the D_max_ analysis, the center of density analysis, or both. RPM stands for “Reads per million.”.
(TIFF)

S15 Fig PF-06446846-induced expression changes are translational. Reverse-transcriptase qPCR measurement of PF-06446846 target transcript levels in Huh7 cells after (A) 4-hour and (B) 24-hour treatment with 1.5 μM PF-06446846. (C) mRNA-seq vs ribo-seq read counts. PF-06446846-sensitive transcripts are highlighted in red and do not show any bias for TE levels. The green cluster indicates histone-coding transcripts which lack poly-A tails and are thus underrepresented in the mRNA-seq libraries. RPM stands for “Reads per million.” The individual quantitative observations that underlie Fig. S15A-B are in supplementary data S14 Table.
(TIFF)

S1 Table Properties of PF-06446846. (DOCX)

S2 Table Crystal data and structure refinement for PF-06446846 (CCDC 1449348). (DOCX)

S3 Table Atomic coordinates (x 104) and equivalent isotropic displacement parameters (Å2x 103) for PF-06446846. U(eq) is defined as one third of the trace of the orthogonalized Uij tensor.
(DOCX)

S4 Table Bond lengths [Å] and angles [°] for PF-06446846. (DOCX)

S5 Table Bond angles [°] for PF-06446846. (DOCX)

S6 Table Anisotropic displacement parameters (Å^2^x 10^3^) for PF-06446846. The anisotropic displacement factor exponent takes the form:-2π^2^ [h^2^ a*^2^U^11^ +…+ 2 h k a* b* U^12^].
(DOCX)

S7 Table Hydrogen coordinates (x 10^4^) and isotropic displacement parameters (Å^2^x 10 ^3^) for PF-06446846. (DOCX)

S8 Table Secretome protein levels measured by SILAC for Huh7 cells treated with PF-06446846 for 4 hours. (DOCX)

S9 Table Secretome protein levels measured by SILAC for Huh7 cells treated with PF-06446846 for 16 hours. Heavy/Light (H/L) Ratio, 1.25 μM PF-06446846 vs vehicle; Medium/Light (M/L) Ratio, 0.25 μM PF-06446846 vs. vehicle.
(DOCX)

S10 Table Cellular proteome levels measured by SILAC for Huh7 cells treated with PF-06446846 for 4 or 16 hours. (XLSX)

S11 Table Total plasma concentration of PF-06446846 in rats measured at 1, 3, 6 and 24 hours following oral administration on study days 1 and 12. (DOCX)

S12 Table PF-06446846-induced stalls after 60-minute treatment. “Z-score” columns refer to the Z-score for the Dmax value, and the changes in read desnity 3’ to the Dmax position with 1.5 and 0.3 μM PF-06446846 treatment, respectively.
(DOCX)

S13 Table Protein translation levels measured using ribosome profiling. Analysis was done using reads aligning after the Dmax position for genes with a Dmax Z-score > 2.
(XLSX)

S14 Table Individual observations underlying Figs 1B-D, 2, 4E, 5J-M, S9A-D, S10B-F, S11, and S15A-B are in supplementary data table S14. (XLSX)

S1 Text Python scripts for data processing. S1 Python Script. Python script used to generate reference multifasta for the initial Kallisto pseudo-alignments. This script combines 5’UTR, CDS, and 3’UTR sequences downloaded separately from ENSEMBL biomart into a single multifasta. It also generates a reference table with the lengths of each element for every annotated transcript. S2 Python Script. Python script that uses the output from Kallisto to calculate the relative abundances of all the transcript isoforms for each gene. S3 Python Script. Python script that makes several histograms summarizing the transcript abundance data from Kallisto and Python Scripts S2 and S1. S4 Python Script. Python script that uses the output from Python Script S2 to generate a list of all transcript isoforms that are unique (the only detectable isoform rom a given gene), that are the most abundant transcript isoform from a given gene, and that are more than a user-specified fraction of isoforms from a given gene. S5 Python Script. Python script that uses a list output by Python Script S4 and a multifasta output by Python Script S1 and generates a new multifasta that has only the sequences in the list. This multifasta is then used for Bowtie input. S6 Python Script. Python script used to remove alignments from a Bowtie output which align to more positions than an input threshold. S7 Python Script. Python script which calculates the number of reads aligning to each position on each transcript based on inferred P-site position. S8 Python Script. Python script which calculates the average distribution of reads relative to the start and stop codons. S9 Python Script. Python script which converts table of reads aligning to each nucleotide positions table based on codon positions. S10 Python Script. Python script which converts a table with the number of reads mapping to each codon to the number of reads per million total reads mapping to each position. S11 Python Script. Python script which takes as input tables with the number of reads mapping to each codon and calculates cumulative maps, Dmax values and Dmax positons. S12 Python Script. Python script that makes input readtables for DeSeq using only reads mapping downstream from the Dmax positon if Dmax is greater than a given threshold. S13 Python Script. Python script that generates input DeSeq readtables from tables of reads aligning to each nucleotide position. This version inputs reads aligning to the entire transcript. S14 Python Script. Python script that generates input DeSeq readtables from tables of reads aligning to each nucleotide position. This version inputs reads aligning to the annotated CDS only. S15 Python Script. Python script which applies a Z-score transformation to raw readcounts from two biological replicates. S16 Python Script. Python script which applies a Z-score transformation to raw readcounts from three biological replicates. S17 Python Script. Python script which applies a Z-score transformation to Dmax values. S18 Python Script. Python script which applies a Z-score transformation to translational efficiency values. S19 Python Script. Python script which calculates the “center of density” or mean position of reads along each CDS, along with changes in center of density induced by treatments and does Z-score transformations of the changes. S20 Python Script. Python script which takes as input a mutifasta file of transcript sequences and a table with 5’UTR, CDS and 3’UTR lengths and identifies all potential uORFs with AUG’s in the 5’UTR and an in-frame stop codon. S21 Python Script. Python script which calculates the number of reads aligning to each specific uORF, the three nucleotide periodicity of each potential uORF and the change between treatment and vehicle datasets.
(TXT)

## Abbreviations

ALT: alanine transaminase
AST: aspartate transaminase
CDH1: cadherin-1
CDS: coding DNA sequence
CFR: cumulative fractional read
CVD: cardiovascular disease
EMCV IRES: encephalomyocarditis virus internal ribosome entry site
FBS: fetal bovine serum
FDR: false discovery rate
HDL: high-density lipoprotein
IFI30: interferon gamma–inducible protein 30
LDL: low-density lipoprotein
LDL-C: low-density lipoprotein cholesterol
LDLR: LDL-receptor
mAb: monoclonal antibody
MMRM: mixed model repeated measure
mTOR: mammalian target of Rapamycin
NMR: normalized mean read
ORF: open reading frame
PCSK9: proprotein convertase subtilisin/kexin type 9
RT-qPCR: quantitative reverse transcription PCR
SILAC: stable isotope labeling with amino acids in cell culture
TE: translational efficiency
uORF: upstream open reading frame
