# Correction: Selective stalling of human translation through small-molecule engagement of the ribosome nascent chain

**DOI:** 10.1371/journal.pbio.1002628

**Published:** 2018-04-17

**Authors:** Nathanael G. Lintner, Kim F. McClure, Donna Petersen, Allyn T. Londregan, David W. Piotrowski, Liuqing Wei, Jun Xiao, Michael Bolt, Paula M. Loria, Bruce Maguire, Kieran F. Geoghegan, Austin Huang, Tim Rolph, Spiros Liras, Jennifer A. Doudna, Robert G. Dullea, Jamie H. D. Cate

There is an error in reporting of the optical rotation for N-(3-chloropyridin-2-yl)-N-[(3R)-piperidin-3-yl]-4-(3H-[1,2,3]triazolo[4,5-b]pyridin-3-yl)benzamide (PF-06446846) in the “Synthesis of PF-06446846” subsection of the Materials and methods section. The given value of +55.9° is incorrect; the relevant passage should instead read: “[α]^D^_25_ = -55.3° (c = 1.965, MeOH).”
