# Supplementary figures and images for: Selective stalling of human translation through small-molecule engagement of the ribosome nascent chain
Source: PLoS Biol. 2017 Mar 21;15(3):e2001882. doi: 10.1371/journal.pbio.2001882 (PMC5360235; doi:10.1371/journal.pbio.2001882)

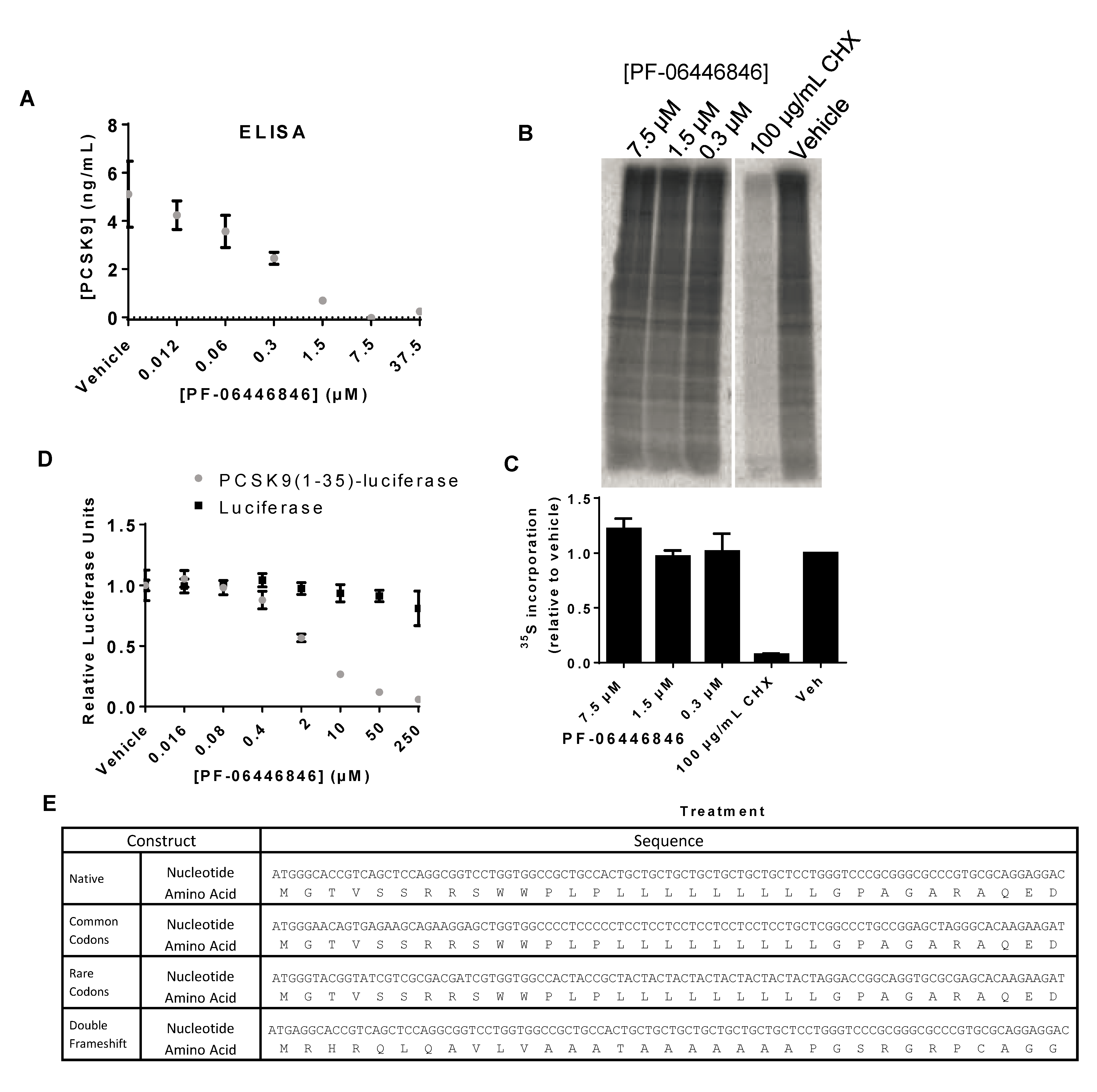

Supplement: S1 Fig — (A) ELISA showing PCSK9 levels in conditioned media from Huh7 cells incubated overnight in the indicated concentrations of PF-06446846. Note that the concentrations of PF-06446846 increase in five-fold increments. (B) Representative SDS-PAGE of total lysates of Huh7 cells labelled with 35S Met/Cys in the presence of varying concentrations of PF-06446846. Cycloheximide (CHX) was used as a control. (C) Quantitation 35S incorporation by densitometry, error bars represent one standard deviation of three replicates. (D) Translation inhibition curves for PCSK9(1–35)-luciferase (grey circles) and luciferase alone (black squares) in HeLa-derived cell-free translation reactions. (E) Nucleotide and amino acid sequences of constructs shown in Fig 1C. (TIFF) [file pbio.2001882.s001.tiff]

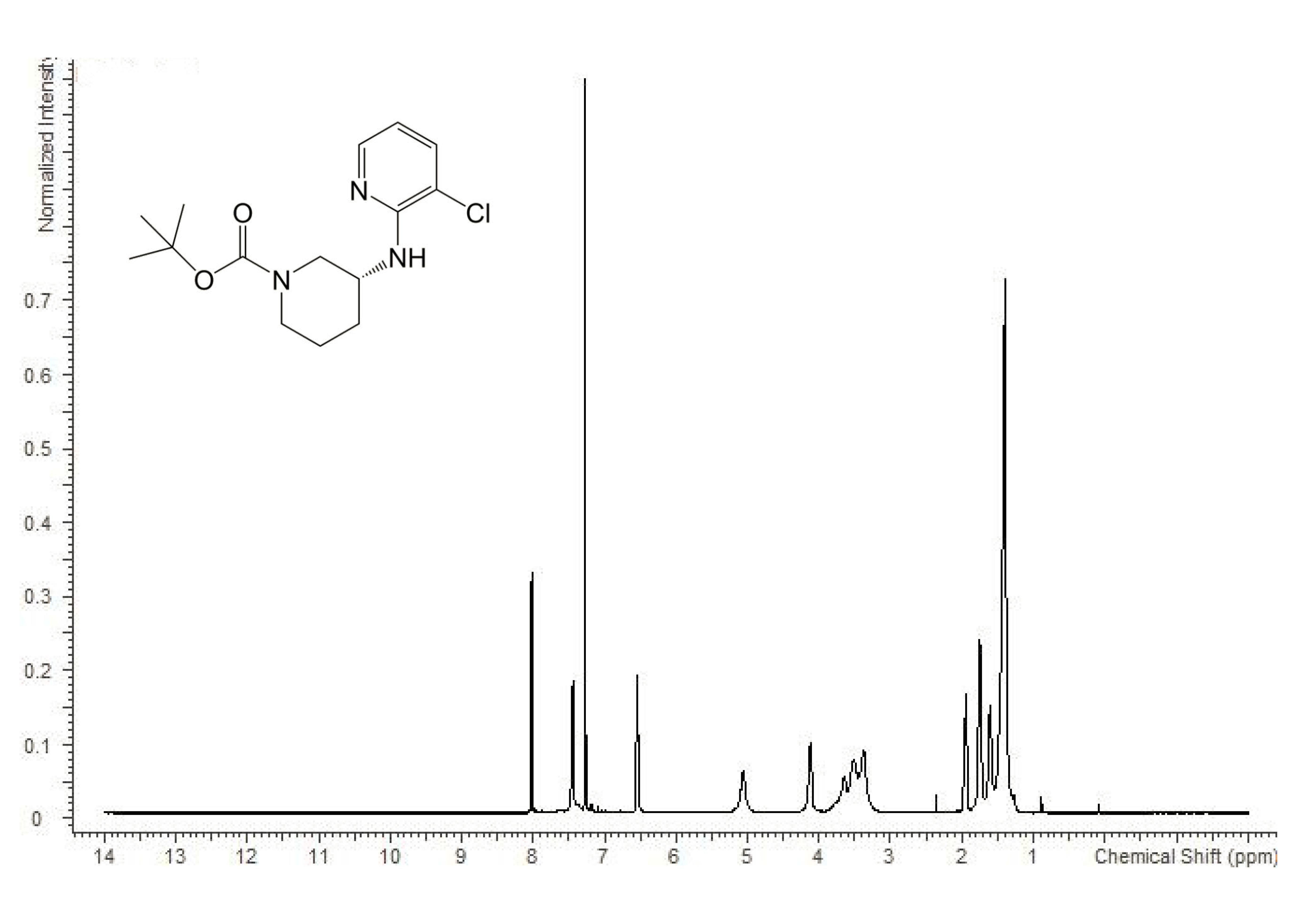

Supplement: S2 Fig — (TIFF) [file pbio.2001882.s002.tiff]

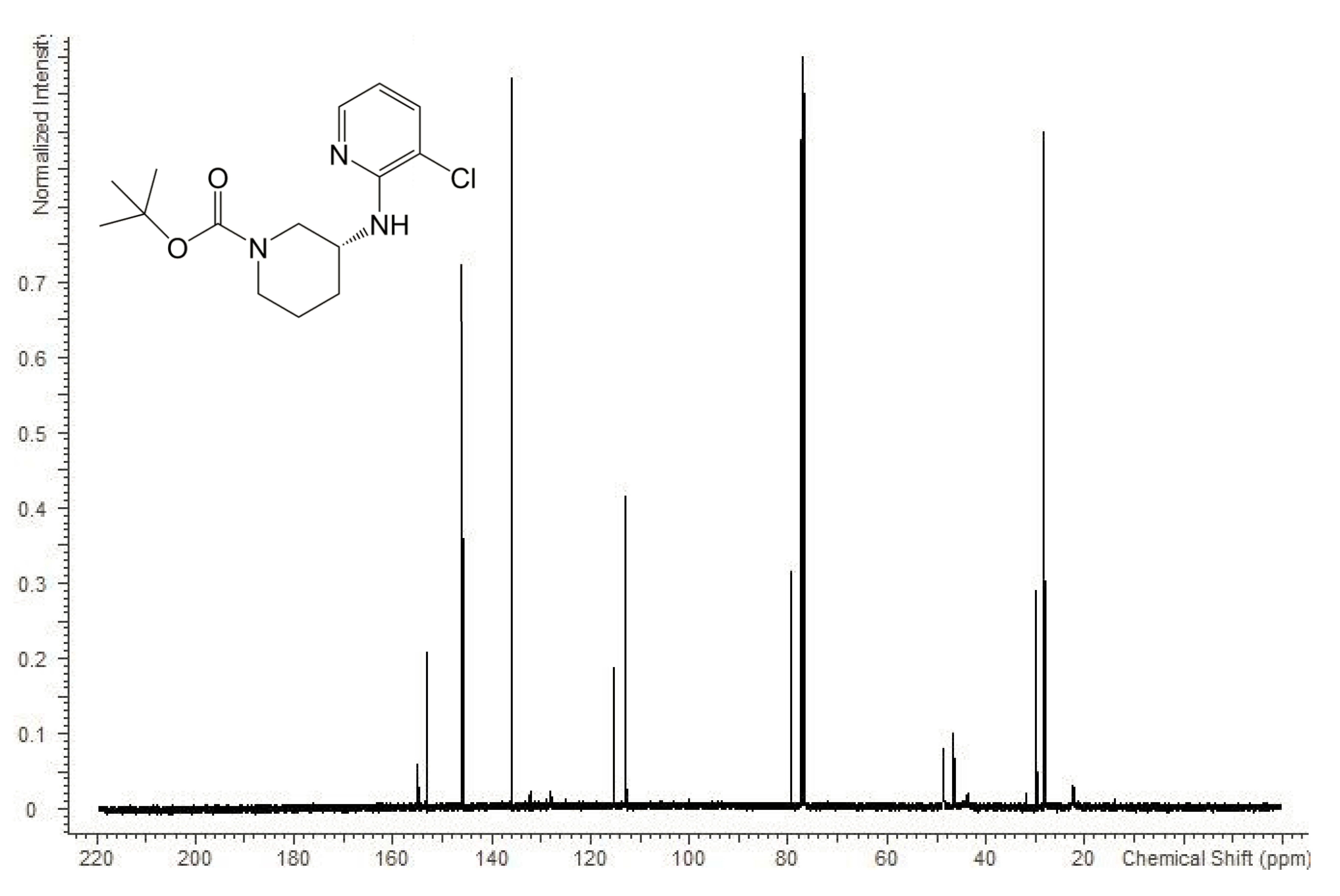

Supplement: S3 Fig — (TIFF) [file pbio.2001882.s003.tiff]

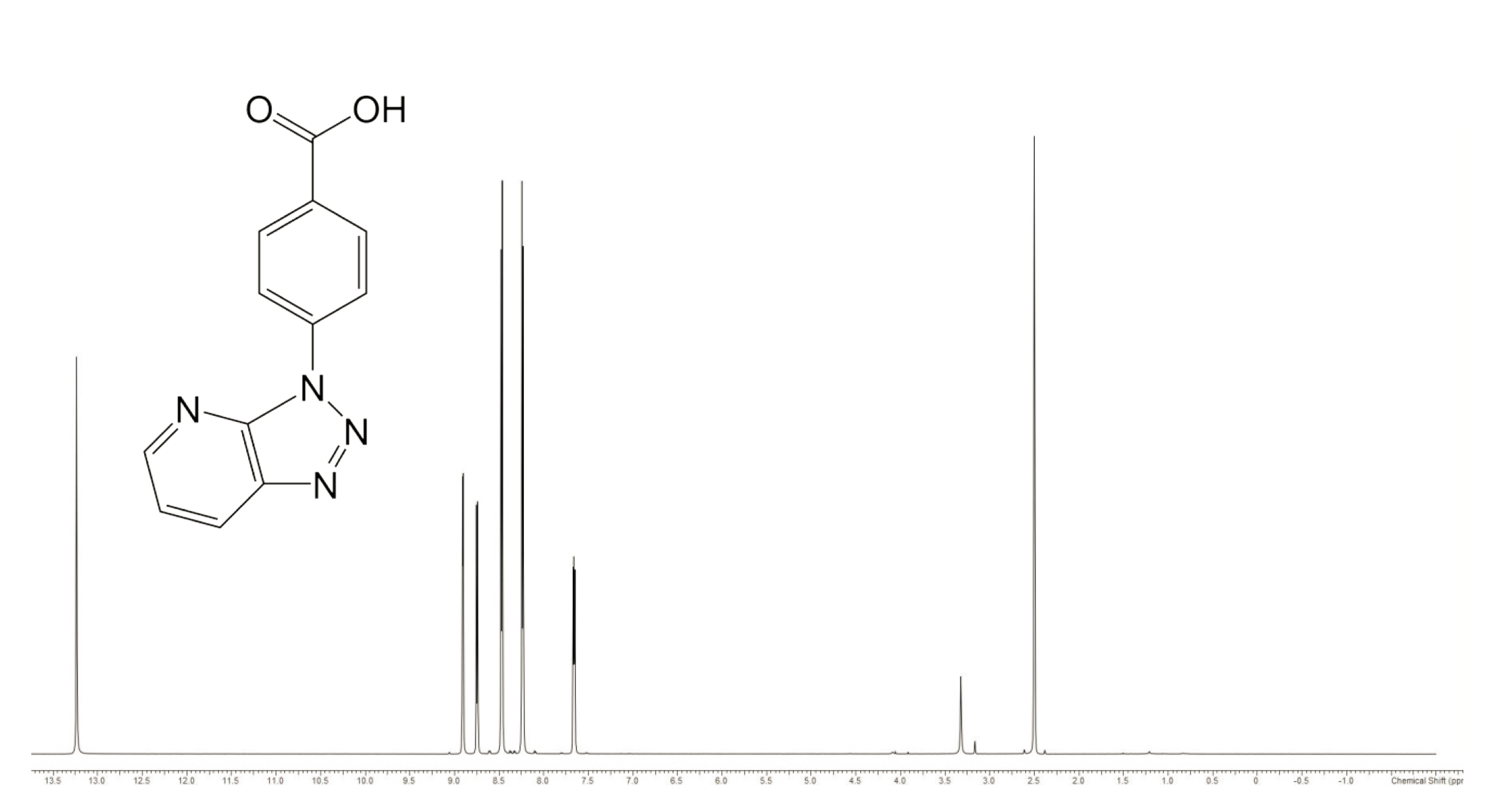

Supplement: S4 Fig — (TIFF) [file pbio.2001882.s004.tiff]

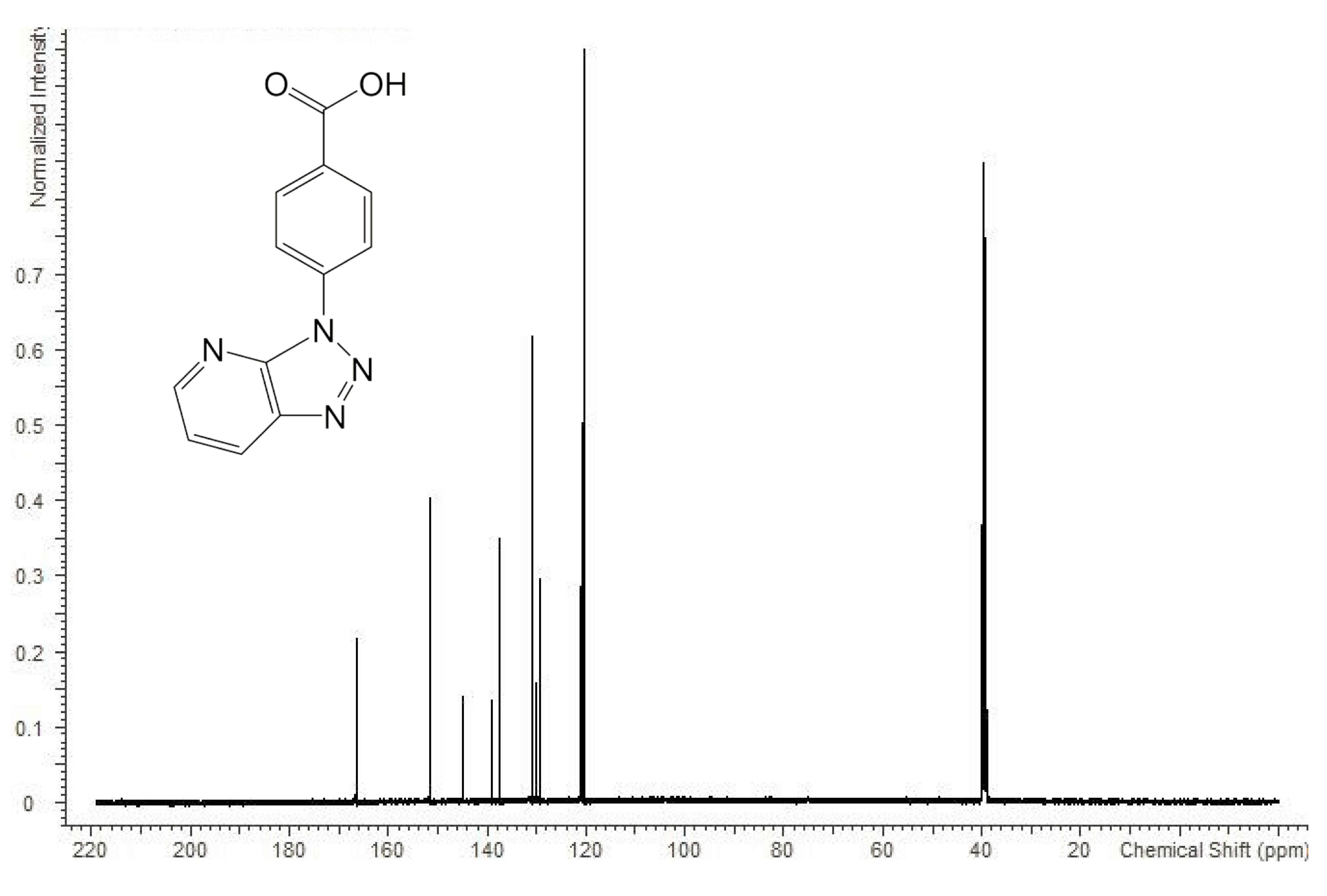

Supplement: S5 Fig — (TIFF) [file pbio.2001882.s005.tiff]

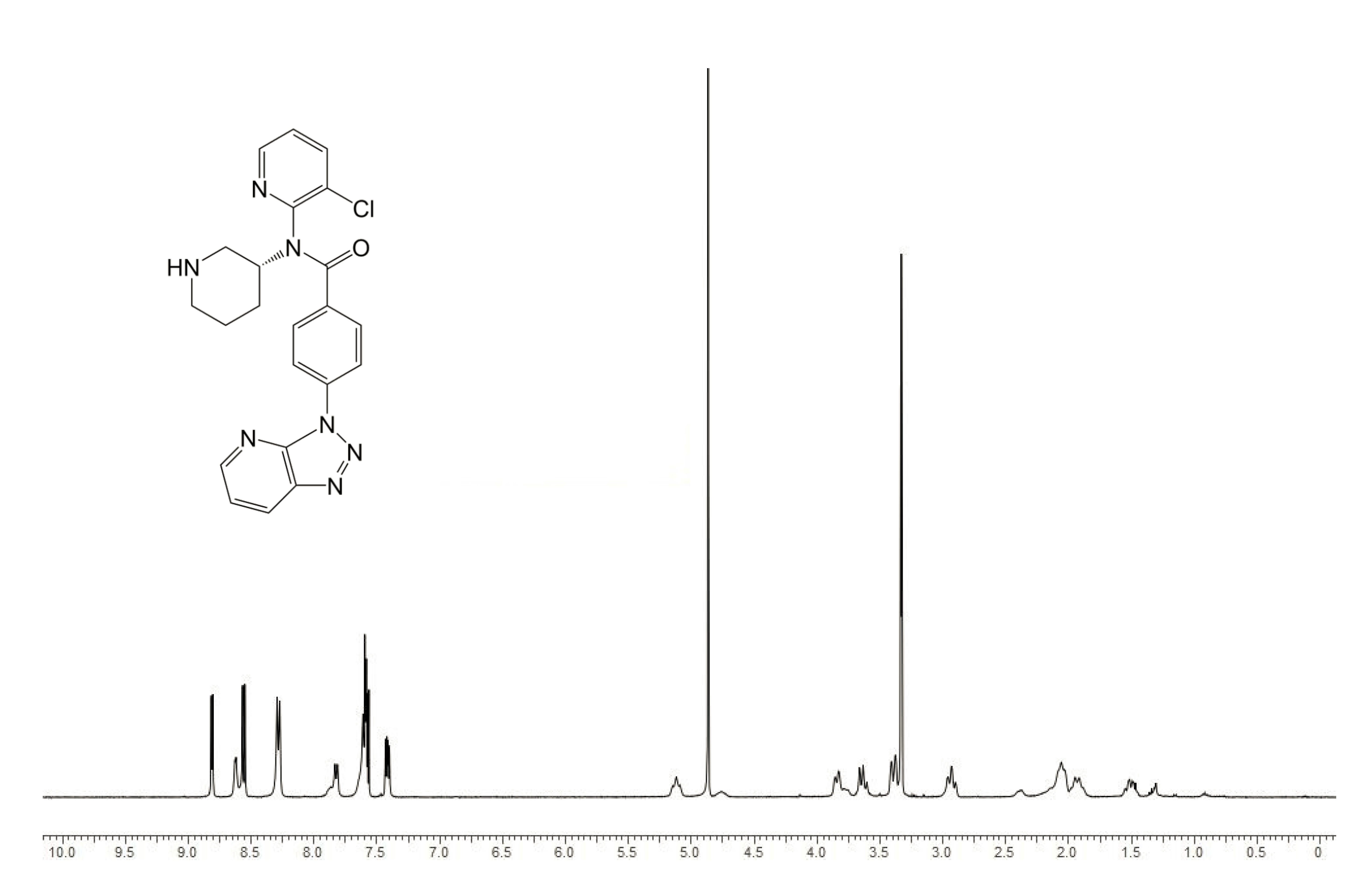

Supplement: S6 Fig — (TIFF) [file pbio.2001882.s006.tiff]

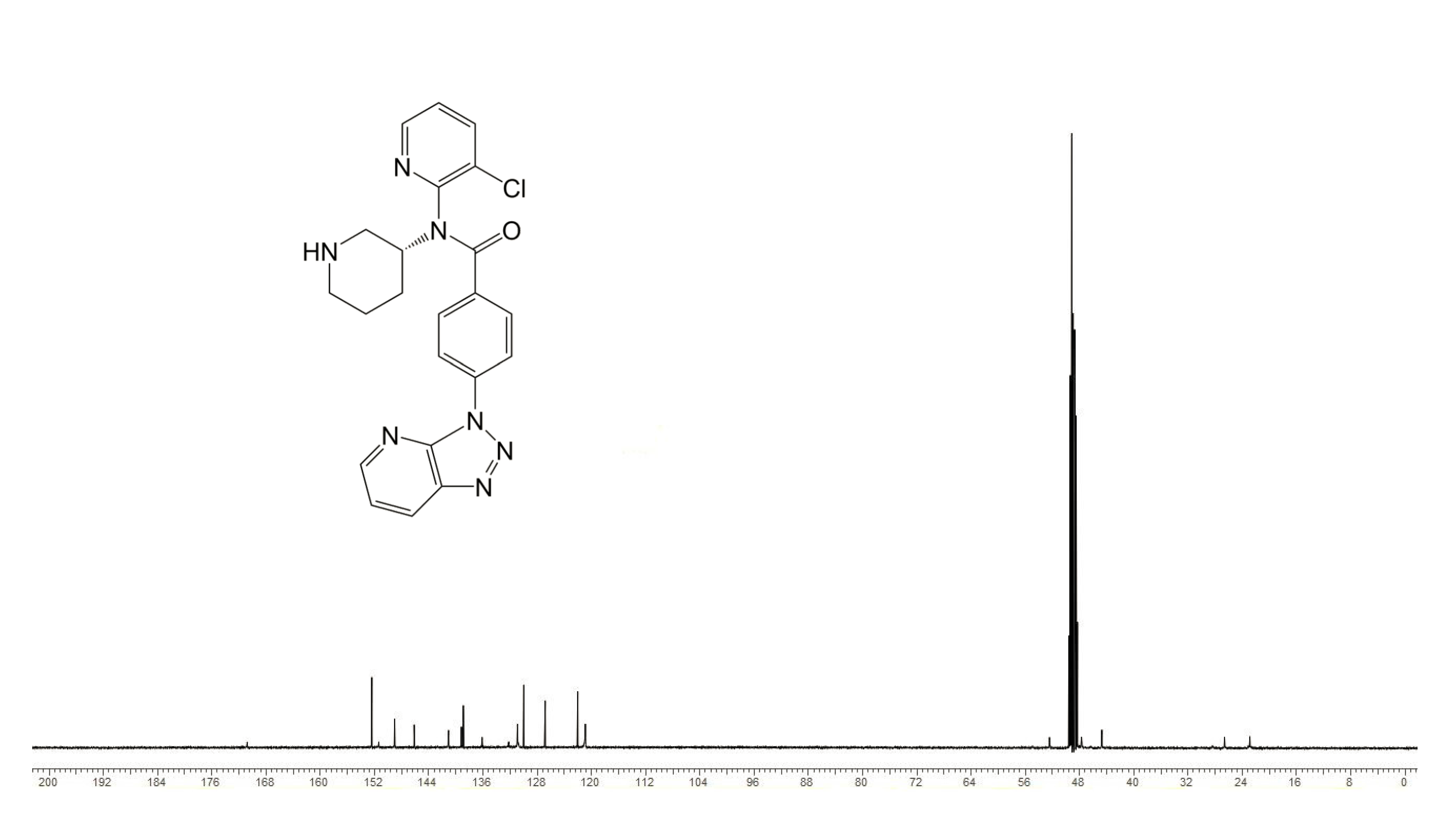

Supplement: S7 Fig — (TIFF) [file pbio.2001882.s007.tiff]

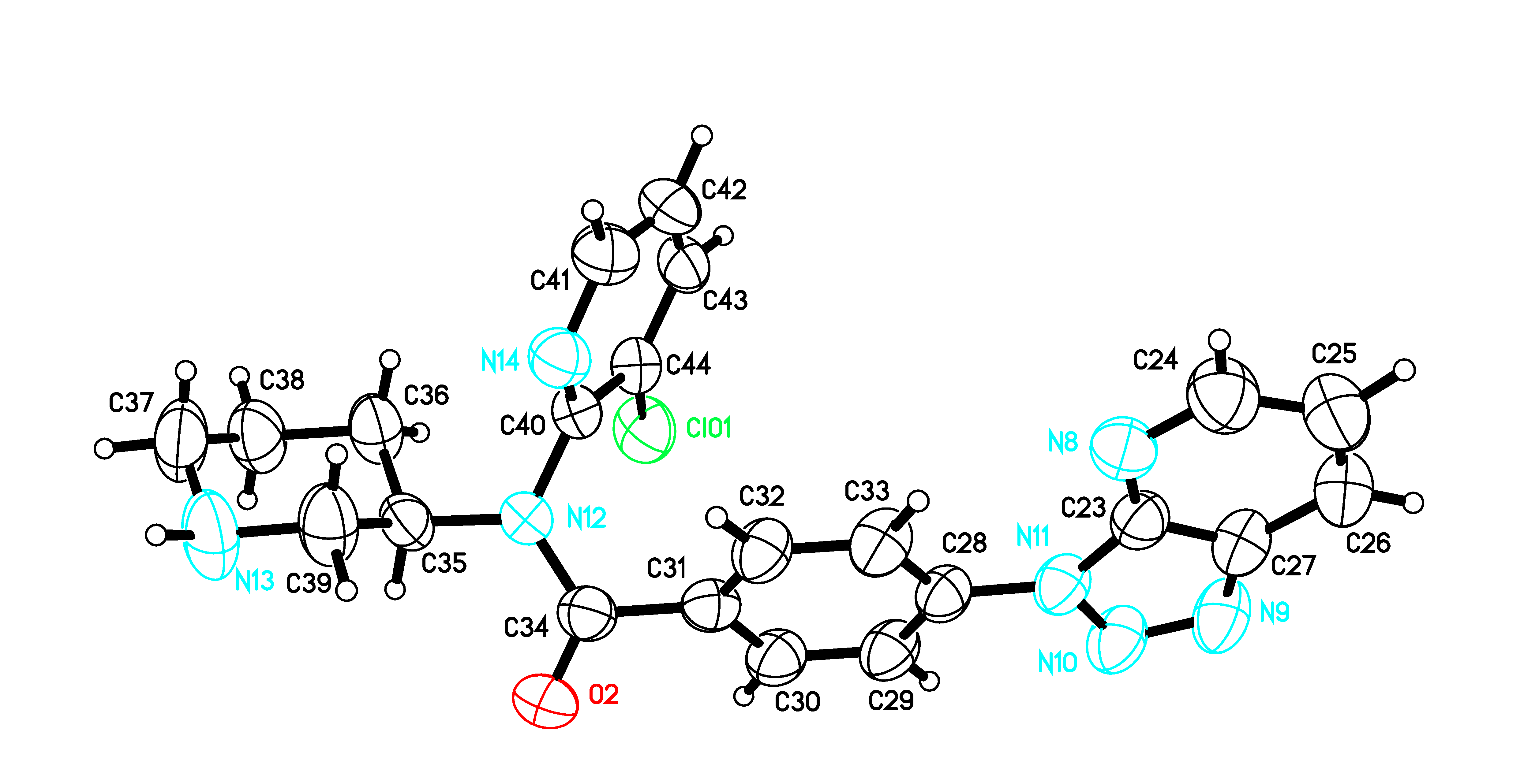

Supplement: S8 Fig — (TIFF) [file pbio.2001882.s008.tiff]

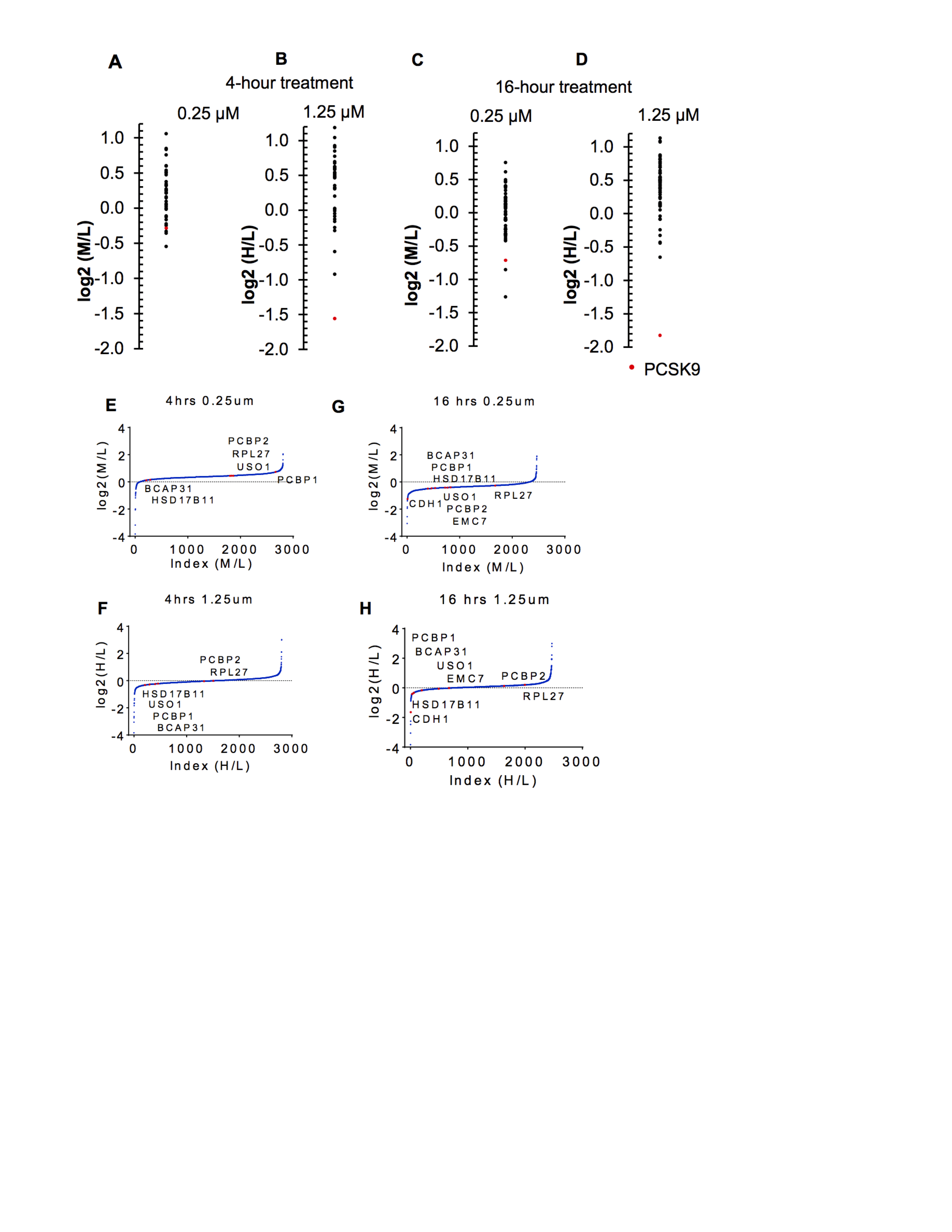

Supplement: S9 Fig — (A-D), Stable isotope labelling by amino acids in cell culture (SILAC) analysis of Huh7 cellular secretome after (A,B) 4-hour and (C,D) 16-hour treatment with 0.25 μM (A,C) or 1.25 μM (B,D) PF-06446846. (E-H), SILAC analysis of the cellular fraction from the experiments shown in panels a-d respectively. Ribosome profiling hits that are detected in the SILAC data are highlighted in red and labelled. (TIFF) [file pbio.2001882.s009.tiff]

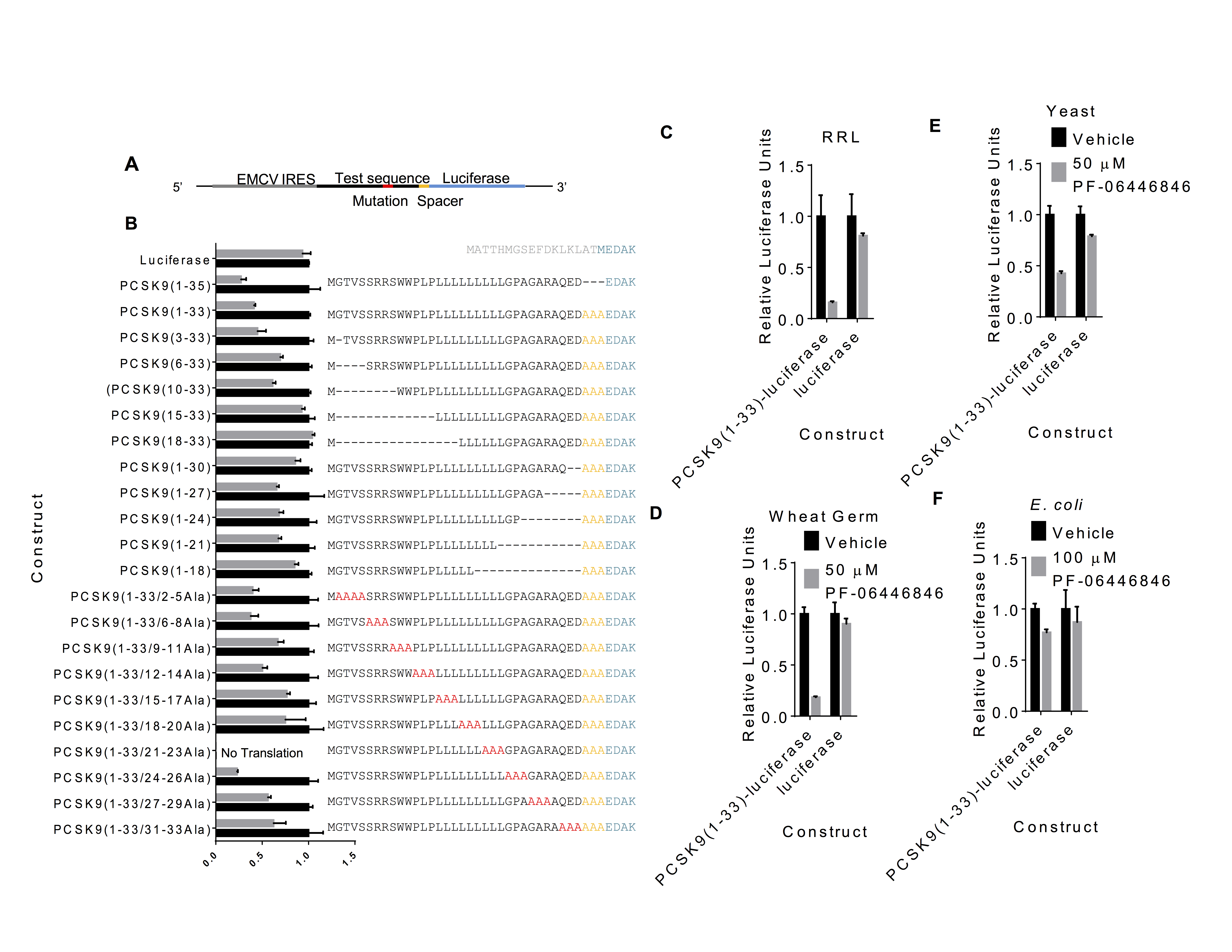

Supplement: S10 Fig — (A) General schematic of mRNA constructs used to program cell-free translation reactions. Sites of mutations are in red and spacer residues are in orange. (B) Constructs, sequences and PF-06446846 sensitivity of PCSK9 mutants. Sequences are colored as in a, with the vector encoded residues in grey for the luciferase construct. Note that PCSK9 residues 34 are 35 are the same as luciferase resides 2 and 3. All error bars represent one standard deviation of three replicates. (C-F), Inhibitory activity of PF-06446846 in (C) Rabbit Reticulocyte Lysate (RRL) (D) Wheat Germ (E) Yeast and (F) E. coli cell-free translation systems. Relative luciferase activities of cell-free translation reactions programmed with PCSK9(1–35)-luciferase in the presence of vehicle (Grey bars) or 50 μM PF’846 panels (C-E) or 100 μM PF-06446846 in (F). All error bars represent one standard deviation of three replicates. The individual quantitative observations that underlie Fig. S10B-F are in supplementary data S14 Table. (TIFF) [file pbio.2001882.s010.tiff]

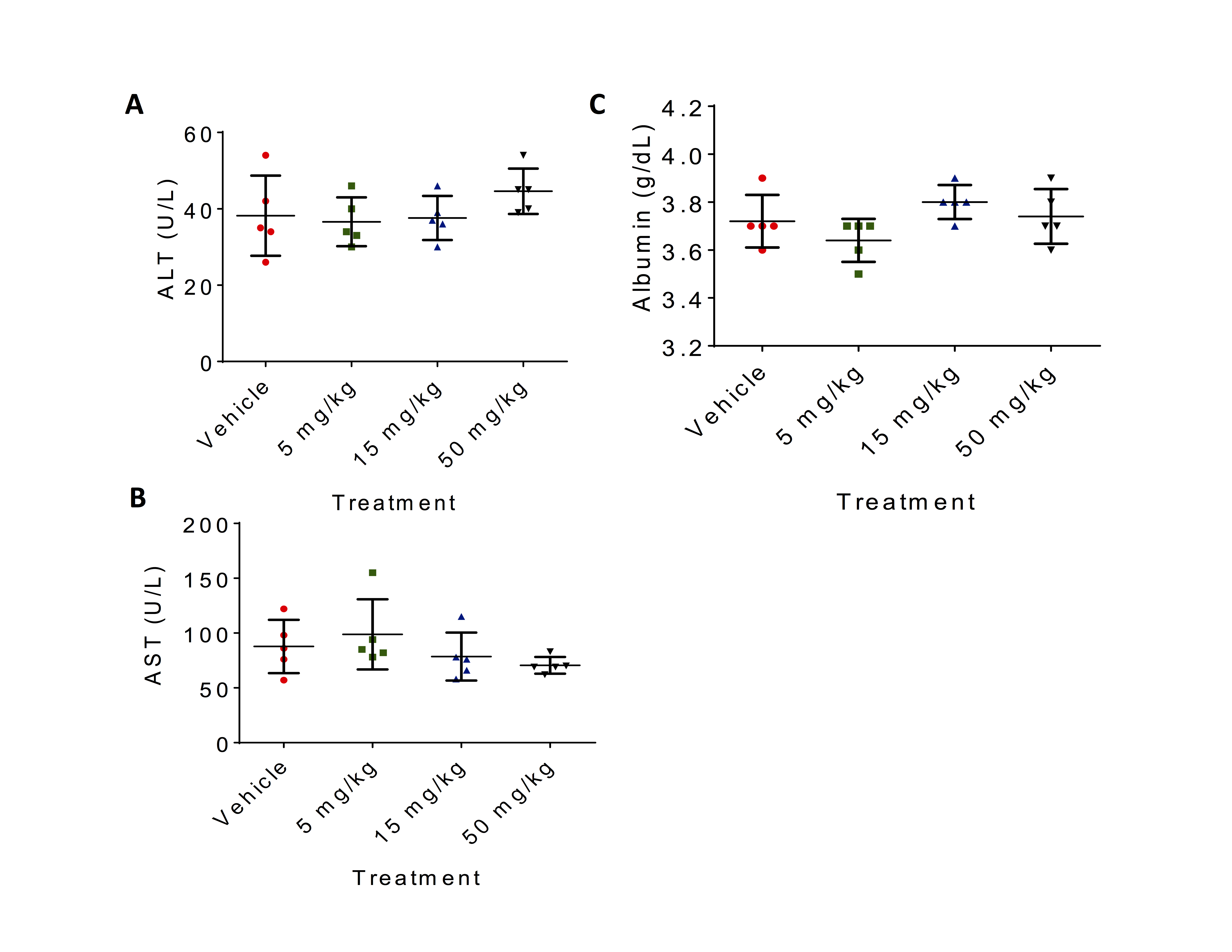

Supplement: S11 Fig — (A) Alanine transaminase (ALT), (B) Aspartate aminotransferase (AST), and (C) Albumin levels in rats measured 24-hours following 14 daily oral doses of PF-06446846. Bars represent group mean + standard deviation. Symbols represent individual animal values. The middle horizontal bar represents the group mean +/- standard deviation. Difference between group means relative to vehicle was performed by a 1-way ANOVA followed by a Dunnett’s multiple comparisons test; * p< = 0.05. The individual quantitative observations that underlie Fig. S11 are in supplementary data S14 Table. (TIFF) [file pbio.2001882.s011.tiff]

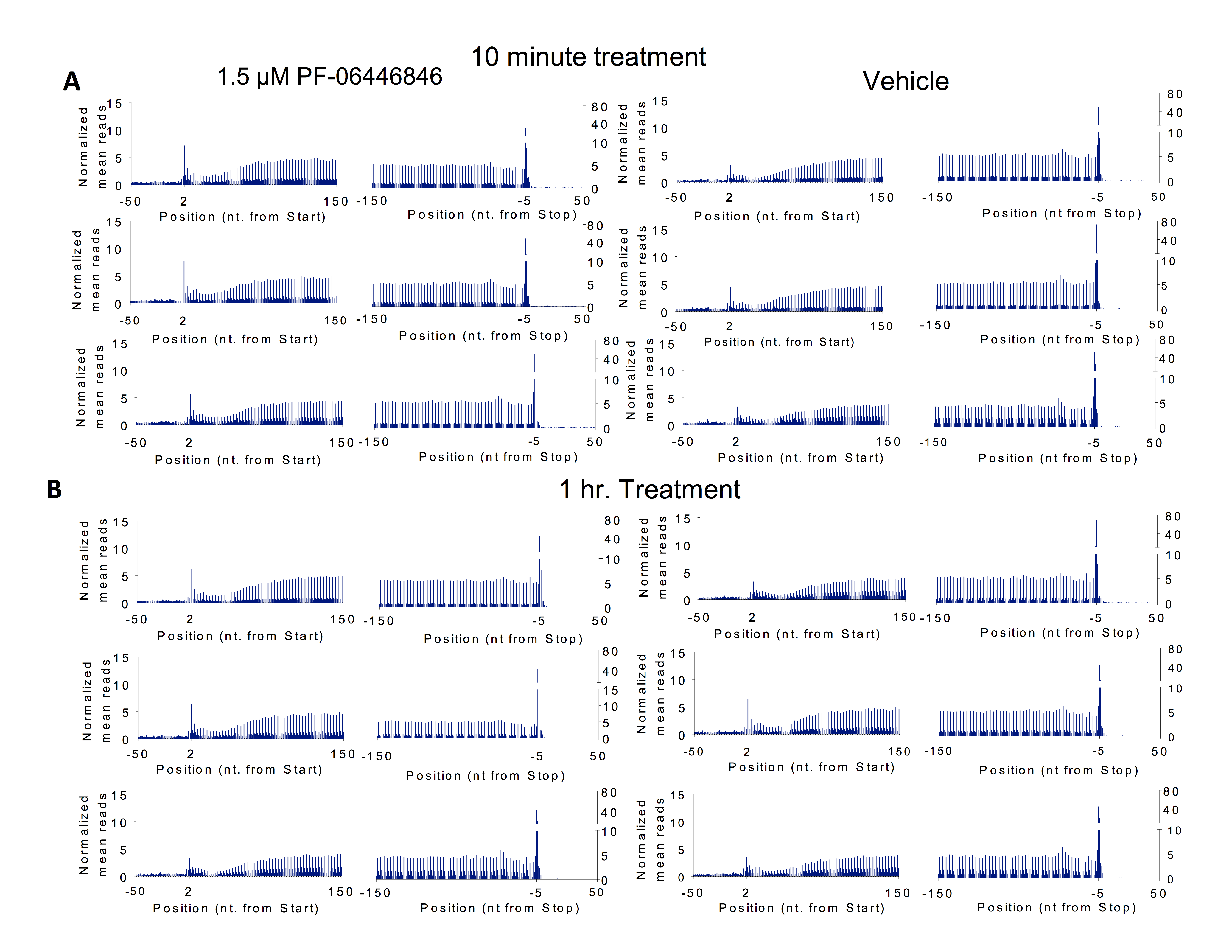

Supplement: S12 Fig — (A) 10 minute datasets (top panel, replicate 1, middle panel; replicate 2, bottom panel replicate 3, left 1.5 μM PF-06446846 treatment, right vehicle treatment). (B) 1 hour treatment, panels arranged as in (A). (C) Riboseq datasets from second experiment. (top panel; replicate 1, bottom panel; replicate 2, Left panels; 1.5 μM PF-06446846 treatment, Right panels; vehicle treatment. (D) mRNA-Seq datasets from second treatments. Panels are arranged as in (C). (TIFF) [file pbio.2001882.s012.tiff]

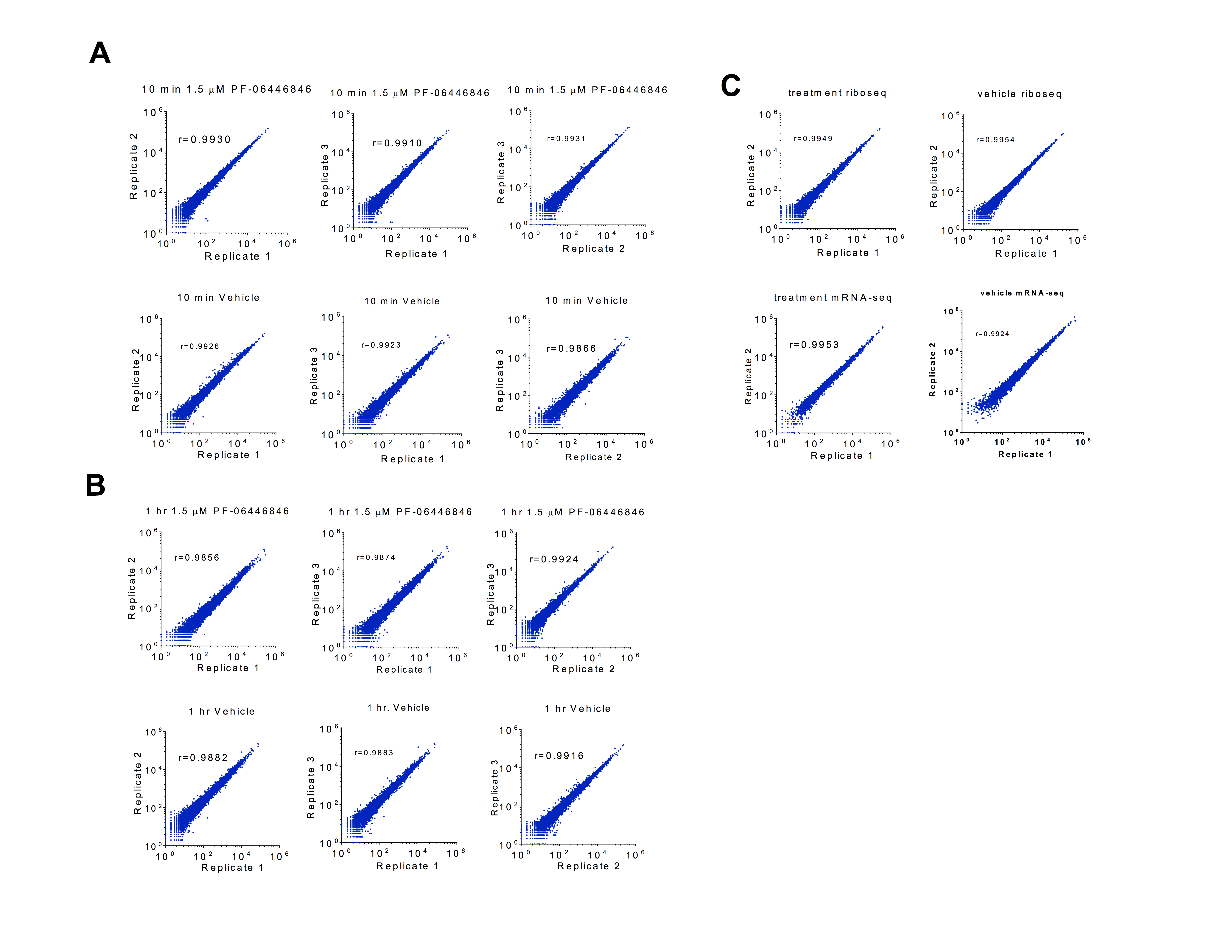

Supplement: S13 Fig — (TIFF) [file pbio.2001882.s013.tiff]

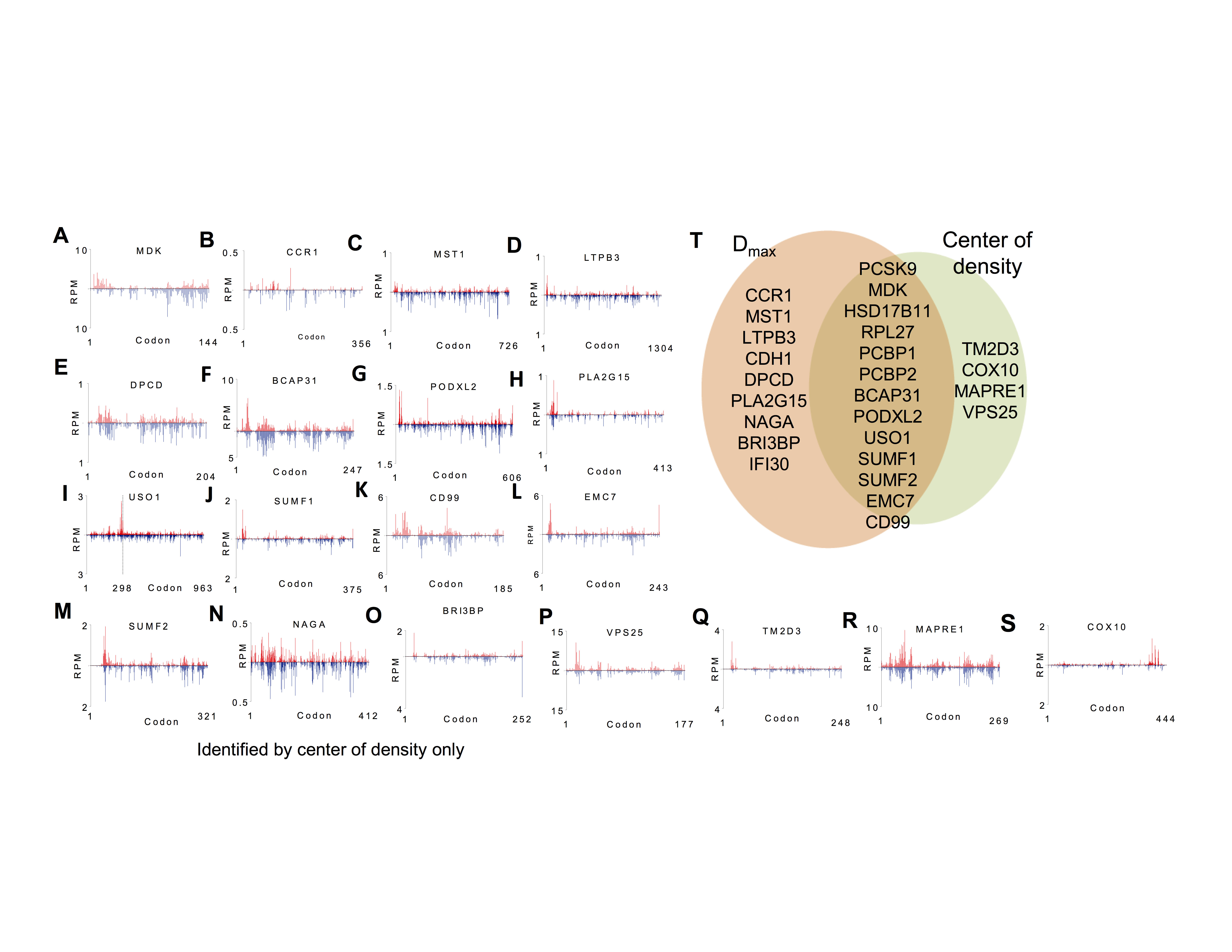

Supplement: S14 Fig — (A-O), (With Fig 5F–5I) Readplots for PF-06446846-sensitive proteins identified using the “Dmax” approach. (P-S), Additional genes displaying a change in the center of density. (P), VPS25, (Q), TM2D3, and (R), MAPRE1. (S) The Cox10 stall occurs near the stop codon. (T) PF-06446846-sensitive sequences identified in the Dmax analysis, the center of density analysis, or both. RPM stands for “Reads per million.”. (TIFF) [file pbio.2001882.s014.tiff]

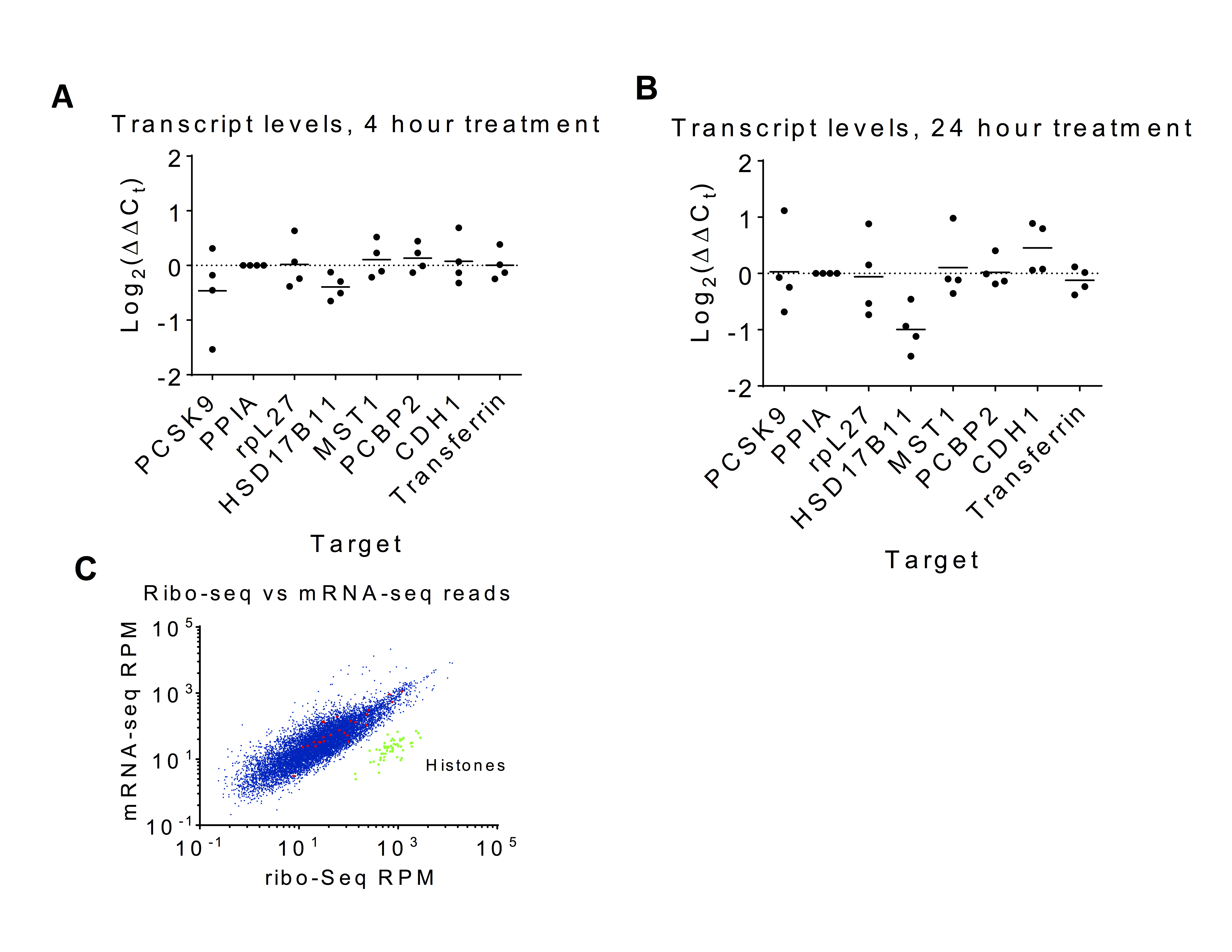

Supplement: S15 Fig — Reverse-transcriptase qPCR measurement of PF-06446846 target transcript levels in Huh7 cells after (A) 4-hour and (B) 24-hour treatment with 1.5 μM PF-06446846. (C) mRNA-seq vs ribo-seq read counts. PF-06446846-sensitive transcripts are highlighted in red and do not show any bias for TE levels. The green cluster indicates histone-coding transcripts which lack poly-A tails and are thus underrepresented in the mRNA-seq libraries. RPM stands for “Reads per million.” The individual quantitative observations that underlie Fig. S15A-B are in supplementary data S14 Table. (TIFF) [file pbio.2001882.s015.tiff]
