# Supplementary material for: Selective stalling of human translation through small-molecule engagement of the ribosome nascent chain
Source: PLoS Biol. 2017 Mar 21;15(3):e2001882. doi: 10.1371/journal.pbio.2001882 (PMC5360235; doi:10.1371/journal.pbio.2001882)
Supplement: S1 Table — (DOCX) [file pbio.2001882.s016.docx]

S1 Table.

Properties of PF-06446846.

|  | **PF-06446846** |
| --- | --- |
| MW | 433.9 |
| ClogP | 2.1 |
| LogD*^a^* | 0.8 |
| Cell Toxicity (µM)*^b^* | >20 |
| RRCK (Papp, 10^-6^ cm/sec)*^c^* | 15.0 |
| Rat liver microsome Cl_int_*^d^* | 18*^e^* |
| Fraction unbound (rat plasma) | 20% |
| Cerep panel of 72 assays screened at 10 µM | 3 targets with  K_i_/IC_50_ < 10 µM |

*^a^*LogD determined by shake flask method. *^b^*CellTiter-Glo ATP viability assay. *^c^*RRCK = Modified Madin-Darby permeability assay. *^d^*intrinsic clearance µL/min/mg. *^e^*Compared to >510 for the initial hit compound^12^.
