# Supplementary material for: Selective stalling of human translation through small-molecule engagement of the ribosome nascent chain
Source: PLoS Biol. 2017 Mar 21;15(3):e2001882. doi: 10.1371/journal.pbio.2001882 (PMC5360235; doi:10.1371/journal.pbio.2001882)
Supplement: S2 Table — (DOCX) [file pbio.2001882.s017.docx]

S2 Table.

Crystal data and structure refinement for **PF-06446846** (CCDC 1449348)

| Crystallization solvent | ethyl acetate/heptane | |
| --- | --- | --- |
| Empirical formula | C_22_H_20_ClN_7_O | |
| Formula weight | 433.90 | |
| Temperature | 273(2) K | |
| Wavelength | 1.54178 Å | |
| Crystal system | Triclinic | |
| Space group | P1 | |
| Unit cell dimensions | a = 8.9633(5) Å | α= 76.136(3)°. |
|  | b = 9.4834(5) Å | β= 79.083(4)°. |
|  | c = 13.9908(8) Å | γ = 66.929(3)°. |
| Volume | 1056.17(10) Å3 | |
| Z | 2 | |
| Density (calculated) | 1.364 Mg/m3 | |
| Absorption coefficient | 1.846 mm-1 | |
| F(000) | 452 | |
| Crystal size | 0.05 x 0.14 x 0.31 mm3 | |
| Theta range for data collection | 3.27 to 67.37°. | |
| Index ranges | -10<=h<=10, -11<=k<=11, -16<=l<=16 | |
| Reflections collected | 7107 | |
| Independent reflections | 4475 [R(int) = 0.0194] | |
| Completeness to theta = 67.37° | 88.0 % | |
| Absorption correction | Empirical | |
| Refinement method | Full-matrix least-squares on F2 | |
| Data / restraints / parameters | 4475 / 5 / 567 | |
| Goodness-of-fit on F2 | 1.028 | |
| Final R indices [I>2sigma(I)] | R1 = 0.0361, wR2 = 0.1009 | |
| R indices (all data) | R1 = 0.0379, wR2 = 0.1034 | |
| Absolute structure parameter | 0.004(13) | |
| Largest diff. peak and hole | 0.200 and -0.170 e.Å-3 | |
