# Supplementary material for: Selective stalling of human translation through small-molecule engagement of the ribosome nascent chain
Source: PLoS Biol. 2017 Mar 21;15(3):e2001882. doi: 10.1371/journal.pbio.2001882 (PMC5360235; doi:10.1371/journal.pbio.2001882)
Supplement: S3 Table — U(eq) is defined as one third of the trace of the orthogonalized Uij tensor. (DOCX) [file pbio.2001882.s018.docx]

**S3 Table.**

Atomic coordinates ( x 104) and equivalent isotropic displacement parameters (Å2x 103) for **PF-06446846**. U(eq) is defined as one third of the trace of the orthogonalized Uij tensor.

| Atom | x | y | z | U(eq) |
| --- | --- | --- | --- | --- |
| C(1) | 5470(4) | -2414(4) | 11338(3) | 54(1) |
| C(2) | 3490(6) | -2260(5) | 12572(4) | 73(1) |
| C(3) | 3833(6) | -3856(5) | 12719(4) | 82(1) |
| C(4) | 5033(6) | -4773(5) | 12146(4) | 81(1) |
| C(5) | 5897(5) | -4015(4) | 11416(3) | 66(1) |
| C(6) | 6665(4) | -562(4) | 10087(3) | 49(1) |
| C(7) | 7809(5) | -511(4) | 9285(3) | 59(1) |
| C(8) | 7896(4) | 897(4) | 8800(3) | 57(1) |
| C(9) | 6851(4) | 2286(3) | 9108(3) | 48(1) |
| C(10) | 5765(5) | 2197(4) | 9939(3) | 54(1) |
| C(11) | 5676(5) | 780(4) | 10445(3) | 55(1) |
| C(12) | 7038(4) | 3770(4) | 8522(3) | 49(1) |
| C(13) | 5777(4) | 6556(3) | 7812(3) | 50(1) |
| C(14) | 4715(5) | 7910(4) | 8327(3) | 62(1) |
| C(15) | 4618(6) | 9669(4) | 6684(3) | 80(1) |
| C(16) | 4956(5) | 9401(4) | 7734(3) | 71(1) |
| C(17) | 5430(6) | 6882(4) | 6757(3) | 72(1) |
| C(18) | 4082(4) | 4906(3) | 8516(3) | 45(1) |
| C(19) | 1603(5) | 5425(4) | 9459(3) | 59(1) |
| C(20) | 1119(4) | 4618(4) | 8974(3) | 63(1) |
| C(21) | 2133(5) | 3965(4) | 8197(3) | 58(1) |
| C(22) | 3653(4) | 4122(3) | 7953(3) | 49(1) |
| C(23) | 9403(4) | 9018(4) | 1669(3) | 51(1) |
| C(24) | 11440(6) | 8821(5) | 465(4) | 74(1) |
| C(25) | 11099(6) | 10454(5) | 296(4) | 80(1) |
| C(26) | 9816(6) | 11380(5) | 841(3) | 73(1) |
| C(27) | 8940(5) | 10615(4) | 1564(3) | 59(1) |
| C(28) | 8193(4) | 7190(3) | 2898(3) | 48(1) |
| C(29) | 7096(4) | 7136(4) | 3743(3) | 56(1) |
| C(30) | 6995(4) | 5705(4) | 4234(3) | 56(1) |
| C(31) | 8030(4) | 4331(4) | 3914(3) | 48(1) |
| C(32) | 9074(5) | 4413(4) | 3051(3) | 54(1) |
| C(33) | 9183(5) | 5826(4) | 2551(3) | 57(1) |
| C(34) | 7891(4) | 2836(4) | 4520(3) | 51(1) |
| C(35) | 9214(4) | 160(3) | 5400(3) | 51(1) |
| C(36) | 10395(5) | -487(4) | 6179(3) | 63(1) |
| C(37) | 10494(7) | -3133(4) | 6183(4) | 88(2) |
| C(38) | 10262(6) | -1991(4) | 6807(3) | 75(1) |
| C(39) | 9503(6) | -1065(4) | 4794(3) | 72(1) |
| C(40) | 10859(4) | 1778(3) | 4512(2) | 45(1) |
| C(41) | 13315(5) | 1150(4) | 3571(3) | 63(1) |
| C(42) | 13840(4) | 2003(4) | 4024(3) | 62(1) |
| C(43) | 12841(4) | 2740(4) | 4747(3) | 57(1) |
| C(44) | 11312(4) | 2618(3) | 5023(3) | 51(1) |
| Cl(01) | 10045(1) | 3428(1) | 5998(1) | 68(1) |
| Cl(02) | 4929(1) | 3377(1) | 6967(1) | 63(1) |
| N(1) | 4314(4) | -1491(3) | 11882(2) | 61(1) |
| N(2) | 7187(5) | -4541(4) | 10710(3) | 78(1) |
| N(3) | 7554(4) | -3354(3) | 10219(3) | 72(1) |
| N(4) | 6535(4) | -2033(3) | 10577(2) | 55(1) |
| N(5) | 5649(3) | 5045(3) | 8344(2) | 48(1) |
| N(6) | 5530(6) | 8367(4) | 6175(3) | 87(1) |
| N(7) | 3064(3) | 5568(3) | 9253(2) | 54(1) |
| N(8) | 10628(4) | 8075(3) | 1133(2) | 59(1) |
| N(9) | 7626(4) | 11154(3) | 2216(3) | 70(1) |
| N(10) | 7250(4) | 9985(3) | 2730(3) | 64(1) |
| N(11) | 8315(4) | 8641(3) | 2411(2) | 51(1) |
| N(12) | 9301(3) | 1625(3) | 4752(2) | 46(1) |
| N(13) | 9310(5) | -2449(4) | 5467(3) | 86(1) |
| N(14) | 11846(3) | 1052(3) | 3806(2) | 55(1) |
| O(1) | 8391(3) | 3824(3) | 8226(2) | 68(1) |
| O(2) | 6553(3) | 2747(3) | 4811(2) | 68(1) |
