# Supplementary material for: Selective stalling of human translation through small-molecule engagement of the ribosome nascent chain
Source: PLoS Biol. 2017 Mar 21;15(3):e2001882. doi: 10.1371/journal.pbio.2001882 (PMC5360235; doi:10.1371/journal.pbio.2001882)
Supplement: S4 Table — (DOCX) [file pbio.2001882.s019.docx]

S4 Table.

Bond lengths [Å] and angles [°] for **PF-06446846**.

| Bond | Length (Å) |
| --- | --- |
| C(1)-N(1) | 1.314(5) |
| C(1)-N(4) | 1.370(5) |
| C(1)-C(5) | 1.394(5) |
| C(2)-N(1) | 1.351(5) |
| C(2)-C(3) | 1.392(6) |
| C(3)-C(4) | 1.352(7) |
| C(4)-C(5) | 1.394(6) |
| C(5)-N(2) | 1.383(6) |
| C(6)-C(7) | 1.374(5) |
| C(6)-C(11) | 1.382(5) |
| C(6)-N(4) | 1.438(4) |
| C(7)-C(8) | 1.370(5) |
| C(8)-C(9) | 1.397(5) |
| C(9)-C(10) | 1.376(5) |
| C(9)-C(12) | 1.503(4) |
| C(10)-C(11) | 1.386(5) |
| C(12)-O(1) | 1.222(4) |
| C(12)-N(5) | 1.367(4) |
| C(13)-N(5) | 1.485(4) |
| C(13)-C(17) | 1.500(5) |
| C(13)-C(14) | 1.516(5) |
| C(14)-C(16) | 1.531(4) |
| C(15)-N(6) | 1.453(6) |
| C(15)-C(16) | 1.497(6) |
| C(17)-N(6) | 1.476(4) |
| C(18)-N(7) | 1.340(4) |
| C(18)-C(22) | 1.394(4) |
| C(18)-N(5) | 1.431(4) |
| C(19)-N(7) | 1.339(4) |
| C(19)-C(20) | 1.362(5) |
| C(20)-C(21) | 1.378(6) |
| C(21)-C(22) | 1.398(5) |
| C(22)-Cl(02) | 1.716(4) |
| C(23)-N(8) | 1.345(5) |
| C(23)-N(11) | 1.367(4) |
| C(23)-C(27) | 1.382(5) |
| C(24)-N(8) | 1.316(5) |
| C(24)-C(25) | 1.424(6) |
| C(25)-C(26) | 1.368(7) |
| C(26)-C(27) | 1.400(6) |
| C(27)-N(9) | 1.351(5) |
| C(28)-C(33) | 1.390(5) |
| C(28)-C(29) | 1.393(5) |
| C(28)-N(11) | 1.418(4) |
| C(29)-C(30) | 1.395(5) |
| C(30)-C(31) | 1.391(5) |
| C(31)-C(32) | 1.388(5) |
| C(31)-C(34) | 1.506(4) |
| C(32)-C(33) | 1.386(5) |
| C(34)-O(2) | 1.220(4) |
| C(34)-N(12) | 1.364(4) |
| C(35)-N(12) | 1.487(4) |
| C(35)-C(39) | 1.514(5) |
| C(35)-C(36) | 1.514(5) |
| C(36)-C(38) | 1.521(4) |
| C(37)-N(13) | 1.450(6) |
| C(37)-C(38) | 1.478(6) |
| C(39)-N(13) | 1.471(5) |
| C(40)-N(14) | 1.323(4) |
| C(40)-C(44) | 1.395(4) |
| C(40)-N(12) | 1.430(4) |
| C(41)-N(14) | 1.330(5) |
| C(41)-C(42) | 1.390(6) |
| C(42)-C(43) | 1.348(6) |
| C(43)-C(44) | 1.394(5) |
| C(44)-Cl(01) | 1.724(4) |
| N(2)-N(3) | 1.297(5) |
| N(3)-N(4) | 1.372(4) |
| N(9)-N(10) | 1.296(4) |
| N(10)-N(11) | 1.375(4) |
