# Supplementary material for: Selective stalling of human translation through small-molecule engagement of the ribosome nascent chain
Source: PLoS Biol. 2017 Mar 21;15(3):e2001882. doi: 10.1371/journal.pbio.2001882 (PMC5360235; doi:10.1371/journal.pbio.2001882)
Supplement: S5 Table — (DOCX) [file pbio.2001882.s020.docx]

S5 Table.

Bond angles [°] for **PF-06446846**.

| Bond | Angle |
| --- | --- |
| N(1)-C(1)-N(4) | 128.2(3) |
| N(1)-C(1)-C(5) | 127.5(4) |
| N(4)-C(1)-C(5) | 104.3(3) |
| N(1)-C(2)-C(3) | 124.5(4) |
| C(4)-C(3)-C(2) | 121.5(4) |
| C(3)-C(4)-C(5) | 115.6(4) |
| N(2)-C(5)-C(4) | 132.5(4) |
| N(2)-C(5)-C(1) | 109.0(4) |
| C(4)-C(5)-C(1) | 118.5(4) |
| C(7)-C(6)-C(11) | 120.6(3) |
| C(7)-C(6)-N(4) | 119.6(3) |
| C(11)-C(6)-N(4) | 119.8(3) |
| C(8)-C(7)-C(6) | 119.6(3) |
| C(7)-C(8)-C(9) | 121.3(4) |
| C(10)-C(9)-C(8) | 118.0(3) |
| C(10)-C(9)-C(12) | 124.8(3) |
| C(8)-C(9)-C(12) | 117.2(3) |
| C(9)-C(10)-C(11) | 121.3(3) |
| C(6)-C(11)-C(10) | 119.1(4) |
| O(1)-C(12)-N(5) | 121.7(3) |
| O(1)-C(12)-C(9) | 120.6(3) |
| N(5)-C(12)-C(9) | 117.7(3) |
| N(5)-C(13)-C(17) | 110.9(2) |
| N(5)-C(13)-C(14) | 113.2(3) |
| C(17)-C(13)-C(14) | 111.1(3) |
| C(13)-C(14)-C(16) | 109.1(3) |
| N(6)-C(15)-C(16) | 115.0(4) |
| C(15)-C(16)-C(14) | 110.6(3) |
| N(6)-C(17)-C(13) | 114.3(3) |
| N(7)-C(18)-C(22) | 122.3(3) |
| N(7)-C(18)-N(5) | 115.9(3) |
| C(22)-C(18)-N(5) | 121.8(3) |
| N(7)-C(19)-C(20) | 123.5(4) |
| C(19)-C(20)-C(21) | 119.5(3) |
| C(20)-C(21)-C(22) | 118.3(3) |
| C(18)-C(22)-C(21) | 118.5(3) |
| C(18)-C(22)-Cl(02) | 121.6(2) |
| C(21)-C(22)-Cl(02) | 119.8(3) |
| N(8)-C(23)-N(11) | 128.6(3) |
| N(8)-C(23)-C(27) | 126.8(3) |
| N(11)-C(23)-C(27) | 104.6(3) |
| N(8)-C(24)-C(25) | 125.4(5) |
| C(26)-C(25)-C(24) | 119.8(4) |
| C(25)-C(26)-C(27) | 116.0(4) |
| N(9)-C(27)-C(23) | 109.4(3) |
| N(9)-C(27)-C(26) | 131.7(4) |
| C(23)-C(27)-C(26) | 118.9(4) |
| C(33)-C(28)-C(29) | 120.1(3) |
| C(33)-C(28)-N(11) | 120.6(3) |
| C(29)-C(28)-N(11) | 119.3(3) |
| C(28)-C(29)-C(30) | 119.4(3) |
| C(31)-C(30)-C(29) | 120.7(4) |
| C(32)-C(31)-C(30) | 119.0(3) |
| C(32)-C(31)-C(34) | 124.0(3) |
| C(30)-C(31)-C(34) | 116.9(3) |
| C(33)-C(32)-C(31) | 120.9(3) |
| C(32)-C(33)-C(28) | 119.8(4) |
| O(2)-C(34)-N(12) | 122.1(3) |
| O(2)-C(34)-C(31) | 120.3(3) |
| N(12)-C(34)-C(31) | 117.6(3) |
| N(12)-C(35)-C(39) | 111.0(3) |
| N(12)-C(35)-C(36) | 112.7(2) |
| C(39)-C(35)-C(36) | 109.8(3) |
| C(35)-C(36)-C(38) | 110.1(3) |
| N(13)-C(37)-C(38) | 109.5(4) |
| C(37)-C(38)-C(36) | 111.2(4) |
| N(13)-C(39)-C(35) | 108.5(3) |
| N(14)-C(40)-C(44) | 122.5(3) |
| N(14)-C(40)-N(12) | 116.5(3) |
| C(44)-C(40)-N(12) | 121.0(3) |
| N(14)-C(41)-C(42) | 122.7(4) |
| C(43)-C(42)-C(41) | 119.2(3) |
| C(42)-C(43)-C(44) | 119.0(3) |
| C(43)-C(44)-C(40) | 118.3(3) |
| C(43)-C(44)-Cl(01) | 120.8(3) |
| C(40)-C(44)-Cl(01) | 120.9(2) |
| C(1)-N(1)-C(2) | 112.5(3) |
| N(3)-N(2)-C(5) | 107.7(3) |
| N(2)-N(3)-N(4) | 109.9(3) |
| C(1)-N(4)-N(3) | 109.1(3) |
| C(1)-N(4)-C(6) | 132.1(3) |
| N(3)-N(4)-C(6) | 118.7(3) |
| C(12)-N(5)-C(18) | 121.2(2) |
| C(12)-N(5)-C(13) | 119.5(3) |
| C(18)-N(5)-C(13) | 118.5(2) |
| C(15)-N(6)-C(17) | 112.4(4) |
| C(19)-N(7)-C(18) | 117.9(3) |
| C(24)-N(8)-C(23) | 113.0(3) |
| N(10)-N(9)-C(27) | 108.6(3) |
| N(9)-N(10)-N(11) | 109.0(3) |
| C(23)-N(11)-N(10) | 108.4(3) |
| C(23)-N(11)-C(28) | 132.0(3) |
| N(10)-N(11)-C(28) | 119.4(3) |
| C(34)-N(12)-C(40) | 122.3(2) |
| C(34)-N(12)-C(35) | 119.3(3) |
| C(40)-N(12)-C(35) | 117.8(2) |
| C(37)-N(13)-C(39) | 112.2(3) |
| C(40)-N(14)-C(41) | 118.3(3) |
