# Supplementary material for: Selective stalling of human translation through small-molecule engagement of the ribosome nascent chain
Source: PLoS Biol. 2017 Mar 21;15(3):e2001882. doi: 10.1371/journal.pbio.2001882 (PMC5360235; doi:10.1371/journal.pbio.2001882)
Supplement: S6 Table — The anisotropic displacement factor exponent takes the form:-2π2 [h2 a*2U11 +…+ 2 h k a* b* U12]. (DOCX) [file pbio.2001882.s021.docx]

S6 Table.

Anisotropic displacement parameters (Å2x 103) for **PF-06446846**. The anisotropic

displacement factor exponent takes the form: -2π2[ h2 a*2U11 + ... + 2 h k a* b* U12 ]

| Atom | Anisotropic displacement parameters | | | | | |
| --- | --- | --- | --- | --- | --- | --- |
|  | U11 | U22 | U33 | U23 | U13 | U12 |
| C(1) | 53(2) | 50(2) | 55(2) | -3(1) | -15(2) | -15(2) |
| C(2) | 64(2) | 79(2) | 65(3) | -6(2) | 6(2) | -26(2) |
| C(3) | 79(3) | 81(3) | 77(3) | 10(2) | -3(3) | -36(2) |
| C(4) | 92(3) | 59(2) | 89(3) | 5(2) | -13(3) | -32(2) |
| C(5) | 67(2) | 49(2) | 76(3) | -4(2) | -15(2) | -17(2) |
| C(6) | 45(2) | 46(2) | 48(2) | -9(1) | -10(2) | -8(1) |
| C(7) | 53(2) | 49(2) | 62(2) | -16(2) | -5(2) | -4(1) |
| C(8) | 45(2) | 57(2) | 57(2) | -12(2) | 3(2) | -10(1) |
| C(9) | 43(2) | 49(2) | 51(2) | -8(1) | -8(2) | -13(1) |
| C(10) | 58(2) | 46(2) | 48(2) | -13(1) | -1(2) | -10(1) |
| C(11) | 54(2) | 53(2) | 46(2) | -7(1) | 2(2) | -11(1) |
| C(12) | 41(2) | 54(2) | 54(2) | -17(1) | -4(2) | -17(1) |
| C(13) | 50(2) | 46(1) | 61(2) | -4(1) | -5(2) | -27(1) |
| C(14) | 76(2) | 52(2) | 65(2) | -13(1) | -2(2) | -30(2) |
| C(15) | 104(3) | 62(2) | 84(3) | 10(2) | -30(3) | -47(2) |
| C(16) | 92(3) | 48(2) | 80(3) | -7(2) | -15(2) | -36(2) |
| C(17) | 109(3) | 68(2) | 55(2) | -6(2) | -2(2) | -55(2) |
| C(18) | 38(2) | 41(1) | 55(2) | -4(1) | -6(2) | -16(1) |
| C(19) | 48(2) | 55(2) | 67(2) | -6(2) | 5(2) | -20(2) |
| C(20) | 47(2) | 55(2) | 86(3) | 2(2) | -11(2) | -26(2) |
| C(21) | 64(2) | 48(2) | 68(2) | 4(2) | -18(2) | -30(2) |
| C(22) | 53(2) | 38(1) | 57(2) | 1(1) | -14(2) | -21(1) |
| C(23) | 49(2) | 51(2) | 48(2) | -4(1) | -7(2) | -14(1) |
| C(24) | 74(3) | 74(2) | 69(3) | -12(2) | 3(2) | -28(2) |
| C(25) | 82(3) | 81(3) | 80(3) | 1(2) | -4(3) | -43(2) |
| C(26) | 81(3) | 58(2) | 76(3) | 5(2) | -13(3) | -29(2) |
| C(27) | 65(2) | 50(2) | 54(2) | -3(1) | -10(2) | -16(2) |
| C(28) | 49(2) | 47(2) | 44(2) | -3(1) | -8(2) | -13(1) |
| C(29) | 53(2) | 50(2) | 53(2) | -10(1) | -1(2) | -7(1) |
| C(30) | 47(2) | 57(2) | 53(2) | -6(2) | 0(2) | -13(2) |
| C(31) | 44(2) | 53(2) | 48(2) | -9(1) | -8(2) | -16(1) |
| C(32) | 57(2) | 47(2) | 50(2) | -10(1) | -2(2) | -11(1) |
| C(33) | 61(2) | 51(2) | 45(2) | -9(1) | 0(2) | -11(2) |
| C(34) | 47(2) | 55(2) | 53(2) | -8(1) | -7(2) | -20(1) |
| C(35) | 49(2) | 51(2) | 58(2) | -9(1) | 1(2) | -26(1) |
| C(36) | 85(2) | 57(2) | 59(2) | -1(1) | -19(2) | -39(2) |
| C(37) | 116(3) | 49(2) | 107(4) | 9(2) | -46(3) | -36(2) |
| C(38) | 94(3) | 68(2) | 70(2) | 10(2) | -22(2) | -44(2) |
| C(39) | 105(3) | 59(2) | 65(2) | -6(2) | -22(2) | -41(2) |
| C(40) | 44(2) | 41(1) | 48(2) | -2(1) | -3(2) | -19(1) |
| C(41) | 51(2) | 63(2) | 69(3) | -15(2) | 5(2) | -20(2) |
| C(42) | 46(2) | 66(2) | 70(3) | 0(2) | 2(2) | -27(2) |
| C(43) | 56(2) | 51(2) | 71(3) | 2(2) | -18(2) | -30(2) |
| C(44) | 52(2) | 41(1) | 59(2) | -6(1) | -7(2) | -19(1) |
| Cl(01) | 76(1) | 61(1) | 74(1) | -26(1) | -3(1) | -27(1) |
| Cl(02) | 76(1) | 58(1) | 63(1) | -20(1) | 0(1) | -31(1) |
| N(1) | 64(2) | 60(2) | 55(2) | -7(1) | -5(2) | -19(2) |
| N(2) | 84(2) | 44(2) | 91(3) | -11(2) | -5(2) | -13(2) |
| N(3) | 70(2) | 49(2) | 80(2) | -17(2) | -1(2) | -5(1) |
| N(4) | 55(2) | 43(1) | 58(2) | -8(1) | -9(2) | -8(1) |
| N(5) | 41(1) | 46(1) | 59(2) | -9(1) | 0(1) | -22(1) |
| N(6) | 138(4) | 84(2) | 59(2) | 4(2) | -8(2) | -75(2) |
| N(7) | 48(2) | 47(1) | 67(2) | -12(1) | -2(2) | -17(1) |
| N(8) | 51(2) | 60(2) | 60(2) | -11(1) | 3(2) | -18(1) |
| N(9) | 82(2) | 42(1) | 71(2) | -7(1) | -5(2) | -11(1) |
| N(10) | 63(2) | 47(1) | 63(2) | -11(1) | 3(2) | -5(1) |
| N(11) | 52(2) | 44(1) | 48(2) | -7(1) | -3(2) | -10(1) |
| N(12) | 42(1) | 45(1) | 53(2) | -5(1) | -5(1) | -19(1) |
| N(13) | 130(3) | 61(2) | 89(2) | -5(2) | -32(2) | -54(2) |
| N(14) | 50(2) | 55(1) | 60(2) | -13(1) | -1(2) | -21(1) |
| O(1) | 43(1) | 71(1) | 91(2) | -15(1) | -1(1) | -24(1) |
| O(2) | 44(1) | 71(1) | 86(2) | -4(1) | -4(1) | -26(1) |
