# Supplementary material for: Selective stalling of human translation through small-molecule engagement of the ribosome nascent chain
Source: PLoS Biol. 2017 Mar 21;15(3):e2001882. doi: 10.1371/journal.pbio.2001882 (PMC5360235; doi:10.1371/journal.pbio.2001882)
Supplement: S7 Table — (DOCX) [file pbio.2001882.s022.docx]

**S7 Table.** Hydrogen coordinates ( x 104) and isotropic displacement parameters (Å2x 10 3) for **PF-06446846**.

| Atom | x | y | z | U(eq) |
| --- | --- | --- | --- | --- |
| H(2) | 2634 | -1683 | 12978 | 87 |
| H(3) | 3224 | -4302 | 13221 | 98 |
| H(4) | 5266 | -5838 | 12233 | 97 |
| H(7) | 8519 | -1426 | 9074 | 70 |
| H(8) | 8667 | 928 | 8255 | 68 |
| H(10) | 5078 | 3107 | 10166 | 64 |
| H(11) | 4960 | 733 | 11017 | 66 |
| H(13) | 6911 | 6456 | 7798 | 61 |
| H(14A) | 5011 | 7709 | 8992 | 75 |
| H(14B) | 3579 | 8032 | 8375 | 75 |
| H(15A) | 3463 | 9912 | 6678 | 96 |
| H(15B) | 4864 | 10573 | 6317 | 96 |
| H(16A) | 4227 | 10283 | 8036 | 85 |
| H(16B) | 6067 | 9314 | 7745 | 85 |
| H(17A) | 6199 | 6037 | 6437 | 86 |
| H(17B) | 4345 | 6892 | 6752 | 86 |
| H(19) | 881 | 5903 | 9960 | 70 |
| H(20) | 113 | 4509 | 9166 | 75 |
| H(21) | 1814 | 3433 | 7845 | 70 |
| H(24) | 12309 | 8233 | 72 | 88 |
| H(25) | 11746 | 10889 | -183 | 96 |
| H(26) | 9542 | 12454 | 737 | 88 |
| H(29) | 6436 | 8046 | 3978 | 68 |
| H(30) | 6229 | 5670 | 4781 | 67 |
| H(32) | 9709 | 3508 | 2803 | 65 |
| H(33) | 9916 | 5861 | 1987 | 68 |
| H(35) | 8108 | 399 | 5744 | 62 |
| H(36A) | 10152 | 272 | 6599 | 76 |
| H(36B) | 11499 | -687 | 5861 | 76 |
| H(37A) | 11589 | -3427 | 5841 | 106 |
| H(37B) | 10357 | -4064 | 6595 | 106 |
| H(38A) | 11081 | -2436 | 7269 | 90 |
| H(38B) | 9198 | -1765 | 7186 | 90 |
| H(39A) | 8726 | -665 | 4307 | 86 |
| H(39B) | 10594 | -1338 | 4446 | 86 |
| H(41) | 14021 | 626 | 3084 | 75 |
| H(42) | 14867 | 2064 | 3830 | 74 |
| H(43) | 13167 | 3321 | 5056 | 68 |
| H(99A) | 9530(50) | -3220(40) | 5070(30) | 88(12) |
| H(99B) | 6700(30) | 8240(60) | 6010(40) | 130(20) |
