# Supplementary material for: Selective stalling of human translation through small-molecule engagement of the ribosome nascent chain
Source: PLoS Biol. 2017 Mar 21;15(3):e2001882. doi: 10.1371/journal.pbio.2001882 (PMC5360235; doi:10.1371/journal.pbio.2001882)
Supplement: S8 Table — (DOCX) [file pbio.2001882.s023.docx]

**S8 Table. Stable isotope labelling with amino acids in cell culture (SILAC) results showing the effects of 4-hour Treatment with PF-06446846 on Human Secreted Proteins in Secretome of Huh7 Cells**

Heavy/Light (H/L) Ratio, 1.25 μM PF-06446846 vs vehicle; Medium/Light (M/L) Ratio, 0.25 μM PF-06446846 vs vehicle.

| **Accession** | **Description** | **Sequence**  **Coverage (%)** | **Unique**  **Peptides** | **Peptides** | **PSMs** | **log_2_(H/L)**  **NORM** | **Number of**  **SILAC Values** | **log_2_(M/L)**  **NORM** |
| --- | --- | --- | --- | --- | --- | --- | --- | --- |
| Q8NBP7 | **Proprotein convertase subtilisin/kexin type 9** | 58 | 5 | 14 | 16 | -1.56 | 10 | -0.28 |
| P05154 | Plasma serine protease inhibitor | 68 | 5 | 5 | 107 | -0.92 | 16 | -0.22 |
| P07339 | Cathepsin D | 26 | 4 | 12 | 123 | -0.59 | 17 | -0.11 |
| P02774 | Vitamin D-binding protein | 24 | 79 | 97 | 1241 | -0.29 | 10 | -0.02 |
| P07237 | Protein disulfide-isomerase | 23 | 4 | 4 | 37 | -0.25 | 1 | -0.55 |
| P07237 | Protein disulfide-isomerase | 79 | 4 | 16 | 37 | -0.25 | 45 | -0.55 |
| Q13162 | Peroxiredoxin-4 | 13 | 4 | 6 | 142 | -0.16 | 7 | -0.25 |
| P02679 | Isoform Gamma-A of Fibrinogen gamma chain | 72 | 23 | 25 | 67 | -0.13 | 10 | 0.05 |
| P55145 | Mesencephalic astrocyte-derived neurotrophic factor | 26 | 7 | 11 | 19 | -0.09 | 11 | 0.16 |
| P04004 | Vitronectin | 78 | 33 | 40 | 89 | -0.05 | 493 | 0.22 |
| P07355 | Annexin A2 | 21 | 4 | 6 | 146 | -0.01 | 23 | 0.02 |
| P04114 | Apolipoprotein B-100 | 60 | 17 | 17 | 579 | 0.02 | 374 | 0.00 |
| P02649 | Apolipoprotein E | 13 | 4 | 6 | 444 | 0.03 | 219 | 0.22 |
| P02652 | Apolipoprotein A-II | 17 | 5 | 18 | 38 | 0.20 | 6 | -0.16 |
| P62937 | Peptidyl-prolyl cis-trans isomerase A | 12 | 6 | 6 | 1057 | 0.31 | 7 | -0.33 |
| O95750 | Fibroblast growth factor 19 | 79 | 4 | 16 | 12 | 0.32 | 1 | -0.36 |
| P01024 | Complement C3 | 44 | 7 | 7 | 2055 | 0.32 | 13 | 0.32 |
| P02787 | Serotransferrin | 47 | 16 | 18 | 1284 | 0.35 | 172 | 0.09 |
| P08603 | Complement factor H | 19 | 4 | 6 | 150 | 0.47 | 168 | 0.60 |
| P02647 | Apolipoprotein A-I | 63 | 43 | 47 | 588 | 0.48 | 27 | 0.06 |
| P36955 | Pigment epithelium-derived factor | 14 | 5 | 17 | 326 | 0.48 | 21 | 0.38 |
| P11047 | Laminin subunit gamma-1 | 37 | 5 | 5 | 87 | 0.49 | 135 | 0.47 |
| O75882 | Isoform 3 of Attractin | 70 | 10 | 13 | 93 | 0.51 | 10 | 0.83 |
| P05997 | Collagen alpha-2(V) chain | 19 | 4 | 6 | 173 | 0.52 | 10 | -0.13 |
| P00734 | Prothrombin | 29 | 26 | 26 | 644 | 0.54 | 8 | 0.54 |
| P02760 | Protein AMBP | 26 | 4 | 4 | 419 | 0.55 | 23 | 0.15 |
| P14543 | Isoform 2 of Nidogen-1 | 47 | 52 | 71 | 230 | 0.59 | 926 | 0.32 |
| O95994 | Anterior gradient protein 2 homolog | 34 | 14 | 16 | 49 | 0.60 | 22 | 0.50 |
| P01019 | Angiotensinogen | 23 | 4 | 4 | 41 | 0.61 | 330 | 1.06 |
| P02768 | Serum albumin | 50 | 34 | 89 | 913 | 0.62 | 82 | 0.26 |
| P10909 | Isoform 4 of Clusterin | 23 | 14 | 23 | 343 | 0.63 | 89 | 0.27 |
| P23142 | Isoform C of Fibulin-1 | 14 | 4 | 14 | 315 | 0.68 | 9 | 0.76 |
| P02751 | Isoform 3 of Fibronectin | 45 | 20 | 20 | 1999 | 0.70 | 7 | 0.26 |
| Q92820 | Gamma-glutamyl hydrolase | 22 | 8 | 14 | 25 | 0.78 | 14 | 0.61 |
| P02771 | Alpha-fetoprotein | 70 | 18 | 23 | 2281 | 0.85 | 146 | 0.22 |
| P01009 | Alpha-1-antitrypsin | 35 | 8 | 18 | 254 | 0.91 | 34 | 0.52 |
| P01034 | Cystatin-C | 26 | 5 | 11 | 168 | 0.93 | 10 | 0.34 |
| P61626 | Lysozyme C | 15 | 5 | 5 | 213 | 1.05 | 40 | 0.85 |
| P06744 | Glucose-6-phosphate isomerase | 50 | 20 | 25 | 19 | 1.19 | 161 | 0.25 |
