# Supplementary material for: Selective stalling of human translation through small-molecule engagement of the ribosome nascent chain
Source: PLoS Biol. 2017 Mar 21;15(3):e2001882. doi: 10.1371/journal.pbio.2001882 (PMC5360235; doi:10.1371/journal.pbio.2001882)
Supplement: S9 Table — Heavy/Light (H/L) Ratio, 1.25 μM PF-06446846 vs vehicle; Medium/Light (M/L) Ratio, 0.25 μM PF-06446846 vs. vehicle. (DOCX) [file pbio.2001882.s024.docx]

**S9 Table | SILAC results showing the effect of 16-hour Treatment with PF-06446846 on Human Secreted Proteins in Secretome of Huh7 Cells.** Heavy/Light (H/L) Ratio, 1.25 μM PF-06446846 vs vehicle; Medium/Light (M/L) Ratio, 0.25 μM PF-06446846 vs. vehicle

| **Accession** | **Description** | **Sequence**  **Coverage (%)** | **Unique**  **Peptides** | **Peptides** | **PSMs** | **log_2_(H/L)**  **NORM** | **Number of**  **SILAC Values** | **log_2_(M/L)**  **NORM** |
| --- | --- | --- | --- | --- | --- | --- | --- | --- |
| Q8NBP7 | **Proprotein convertase subtilisin/kexin type 9** | 20 | 11 | 11 | 75 | -1.82 | 7 | -0.71 |
| P02652 | Apolipoprotein A-II | 69 | 7 | 7 | 58 | -0.65 | 1 | -1.26 |
| P02749 | Beta-2-glycoprotein 1 | 34 | 5 | 8 | 170 | -0.44 | 10 | -0.28 |
| P00450 | Ceruloplasmin | 17 | 11 | 16 | 158 | -0.43 | 13 | 0.40 |
| P07339 | Cathepsin D | 43 | 9 | 12 | 217 | -0.33 | 7 | -0.41 |
| Q13162 | Peroxiredoxin-4 | 65 | 9 | 14 | 255 | -0.24 | 14 | 0.20 |
| P55058 | Phospholipid transfer protein | 23 | 6 | 9 | 65 | -0.08 | 1 | -0.86 |
| P02774 | Vitamin D-binding protein | 46 | 11 | 18 | 2102 | -0.08 | 13 | 0.13 |
| P07237 | Protein disulfide-isomerase | 28 | 11 | 11 | 60 | -0.04 | 10 | -0.42 |
| P06744 | Glucose-6-phosphate isomerase | 20 | 4 | 7 | 28 | 0.06 | 3 | 0.75 |
| P01024 | Complement C3 | 64 | 81 | 102 | 5240 | 0.11 | 493 | -0.19 |
| P07355 | Annexin A2 | 67 | 23 | 23 | 310 | 0.11 | 45 | 0.17 |
| P04004 | Vitronectin | 28 | 8 | 13 | 406 | 0.13 | 27 | -0.25 |
| P05154 | Plasma serine protease inhibitor | 24 | 9 | 10 | 113 | 0.14 | 16 | -0.38 |
| P62937 | Peptidyl-prolyl cis-trans isomerase A | 79 | 4 | 16 | 1129 | 0.14 | 22 | 0.01 |
| P55145 | Mesencephalic astrocyte-derived neurotrophic factor | 48 | 8 | 8 | 45 | 0.15 | 9 | 0.09 |
| P01019 | Angiotensinogen | 37 | 11 | 11 | 186 | 0.16 | 23 | -0.31 |
| P01031 | Complement C5 | 19 | 21 | 29 | 249 | 0.23 | 36 | 0.17 |
| P02787 | Serotransferrin | 71 | 48 | 54 | 3501 | 0.24 | 374 | -0.32 |
| Q08830 | Fibrinogen-like protein 1 | 40 | 5 | 10 | 90 | 0.25 | 10 | -0.34 |
| P02649 | Apolipoprotein E | 78 | 22 | 29 | 996 | 0.29 | 82 | -0.11 |
| Q12805-2 | Isoform 2 of EGF-containing fibulin-like extracellular matrix protein 1 | 38 | 4 | 13 | 322 | 0.32 | 10 | 0.10 |
| P08603 | Complement factor H | 50 | 41 | 45 | 678 | 0.32 | 94 | 0.22 |
| O75882-2 | Isoform 2 of Attractin | 25 | 10 | 26 | 232 | 0.35 | 14 | 0.08 |
| P02768 | Serum albumin | 70 | 35 | 40 | 2141 | 0.37 | 172 | -0.25 |
| P00742 | Coagulation factor X | 27 | 6 | 11 | 86 | 0.38 | 8 | 0.06 |
| P02679-2 | Isoform Gamma-A of Fibrinogen gamma chain | 51 | 13 | 15 | 235 | 0.39 | 39 | 0.19 |
| P00734 | Prothrombin | 48 | 15 | 23 | 1179 | 0.41 | 71 | 0.14 |
| P05997 | Collagen alpha-2(V) chain | 24 | 8 | 24 | 318 | 0.43 | 17 | -0.32 |
| P02647 | Apolipoprotein A-I | 85 | 29 | 31 | 1391 | 0.43 | 146 | -0.11 |
| P04114 | Apolipoprotein B-100 | 68 | 16 | 306 | 6932 | 0.45 | 45 | 0.13 |
| P07942 | Laminin subunit beta-1 | 31 | 22 | 46 | 222 | 0.47 | 12 | 0.18 |
| P02671 | Fibrinogen alpha chain | 20 | 11 | 14 | 89 | 0.47 | 17 | 0.40 |
| P14543-2 | Isoform 2 of Nidogen-1 | 41 | 31 | 42 | 810 | 0.49 | 89 | -0.10 |
| P02675 | Fibrinogen beta chain | 39 | 11 | 14 | 91 | 0.49 | 13 | 0.22 |
| P36955 | Pigment epithelium-derived factor | 46 | 9 | 16 | 601 | 0.50 | 23 | -0.25 |
| P01009 | Alpha-1-antitrypsin | 65 | 25 | 27 | 1113 | 0.50 | 168 | 0.07 |
| P02760 | Protein AMBP | 55 | 18 | 19 | 2359 | 0.51 | 219 | -0.11 |
| P02763 | Alpha-1-acid glycoprotein 1 | 24 | 4 | 4 | 50 | 0.52 | 13 | -0.08 |
| P10909-4 | Isoform 4 of Clusterin | 53 | 24 | 25 | 1114 | 0.53 | 135 | -0.01 |
| P61769 | Beta-2-microglobulin | 67 | 5 | 7 | 57 | 0.54 | 6 | -0.35 |
| P19823 | Inter-alpha-trypsin inhibitor heavy chain H2 | 65 | 38 | 55 | 2770 | 0.54 | 161 | -0.11 |
| P98160 | Basement membrane-specific heparan sulfate proteoglycan core protein | 19 | 31 | 58 | 340 | 0.55 | 21 | -0.03 |
| Q92820 | Gamma-glutamyl hydrolase | 45 | 10 | 12 | 134 | 0.60 | 11 | -0.23 |
| P10599 | Thioredoxin | 66 | 4 | 9 | 151 | 0.61 | 5 | 0.38 |
| O15230 | Laminin subunit alpha-5 | 17 | 43 | 47 | 248 | 0.63 | 30 | 0.23 |
| P01034 | Cystatin-C | 68 | 12 | 12 | 306 | 0.65 | 34 | 0.02 |
| Q92520 | Protein FAM3C | 54 | 4 | 10 | 91 | 0.69 | 6 | 0.34 |
| P02751 | Fibronectin | 61 | 51 | 120 | 5574 | 0.70 | 330 | 0.12 |
| P02771 | Alpha-fetoprotein | 81 | 44 | 50 | 8021 | 0.72 | 926 | -0.08 |
| P11047 | Laminin subunit gamma-1 | 30 | 14 | 35 | 260 | 0.75 | 15 | 0.13 |
| P02655 | Apolipoprotein C-II | 59 | 4 | 6 | 121 | 0.76 | 10 | -0.32 |
| O00391-2 | Isoform 2 of Sulfhydryl oxidase 1 | 21 | 6 | 10 | 32 | 0.78 | 3 | 0.29 |
| P00995 | Pancreatic secretory trypsin inhibitor | 54 | 4 | 4 | 88 | 0.81 | 10 | -0.03 |
| O00468-6 | Isoform 6 of Agrin | 32 | 35 | 46 | 364 | 0.81 | 40 | 0.11 |
| P30990 | Neurotensin/neuromedin N | 66 | 12 | 12 | 82 | 0.82 | 15 | 0.47 |
| P07225 | Vitamin K-dependent protein S | 27 | 13 | 16 | 134 | 0.85 | 15 | 0.50 |
| O95994 | Anterior gradient protein 2 homolog | 42 | 8 | 8 | 63 | 0.87 | 7 | 0.41 |
| O95445-2 | Isoform 2 of Apolipoprotein M | 47 | 5 | 7 | 138 | 0.88 | 7 | -0.10 |
| P02766 | Transthyretin | 76 | 5 | 8 | 130 | 1.07 | 3 | 0.46 |
| P03950 | Angiogenin | 37 | 5 | 5 | 16 | 1.08 | 4 | 0.62 |
| P39060-2 | Isoform 3 of Collagen alpha-1(XVIII) chain | 16 | 10 | 15 | 231 | 1.13 | 14 | 0.24 |
