# Supplementary material for: Selective stalling of human translation through small-molecule engagement of the ribosome nascent chain
Source: PLoS Biol. 2017 Mar 21;15(3):e2001882. doi: 10.1371/journal.pbio.2001882 (PMC5360235; doi:10.1371/journal.pbio.2001882)
Supplement: S11 Table — (DOCX) [file pbio.2001882.s026.docx]

**S11 Table.**

Total plasma concentration of PF-06446846 in rats measured at 1, 3, 6 and 24 hours following oral administration on study days 1 and 12.

| Treatment  (mg/kg/day) | Study Day | Cmax  (ng/mL)*^a^* | | | Tmax  (Hours)*^b^* | | | AUC  (ng*Hours/mL)*^c^* | | |
| --- | --- | --- | --- | --- | --- | --- | --- | --- | --- | --- |
|  |  | Mean*^d^* | S.D. | n | Mean | S.D. | n | Mean | S.D. | n |
| 5 | 1 | 50.0 | 27.6 | 5 | 3.00 | 0.00 | 5 | 493 | 336 | 5 |
|  | 12 | 94.4 | 49.0 | 5 | 3.00 | 0.00 | 5 | 825 | 498 | 5 |
| 15 | 1 | 382 | 101 | 5 | 4.20 | 1.64 | 5 | 4630 | 1770 | 5 |
|  | 12 | 385 | 62.8 | 5 | 4.80 | 1.64 | 5 | 4980 | 934 | 5 |
| 50 | 1 | 1570 | 269 | 5 | 4.80 | 1.64 | 5 | 20200 | 4160 | 5 |
|  | 12 | 1560 | 481 | 5 | 4.80 | 1.64 | 5 | 20200 | 5630 | 5 |

*^a^*Cmax= maximum plasma concentration. *^b^*Tmax= time of maximum plasma concentration. *^c^*AUC= plasma exposure area under the curve 0-24 hours. *^d^*Mean and standard deviation determined using 5 animals.
