# Supplementary material for: Selective stalling of human translation through small-molecule engagement of the ribosome nascent chain
Source: PLoS Biol. 2017 Mar 21;15(3):e2001882. doi: 10.1371/journal.pbio.2001882 (PMC5360235; doi:10.1371/journal.pbio.2001882)
Supplement: S12 Table — “Z-score” columns refer to the Z-score for the Dmax value, and the changes in read desnity 3’ to the Dmax position with 1.5 and 0.3 μM PF-06446846 treatment, respectively. (DOCX) [file pbio.2001882.s027.docx]

**S12 Table.**

**PF-06446846-Induced stalls after 60-minute treatment.** “Z-score” columns refer to the Z-score for the Dmax value, and the changes in read desnity 3’ to the Dmax position with 1.5 and 0.3 µM PF-06446846 treatment respectively.

| Gene | Transcript | Main Stall codon | D_max_ Codon | Z-score (D_max_ value) | Log_2_-fold Change; 1.5 µM^a^ | Z-score (change; 1.5 µM) | Log_2_-fold change; 0.3 µM^a^ | Z-score (change; 0.3 µM) |
| --- | --- | --- | --- | --- | --- | --- | --- | --- |
| MDK | ENST00000395566 | 20 | 32 | 6.7 | -2.3 | -6.1 | -0.8 | -3.7 |
| CCR1 | ENST00000296140 | undef^c^ | 105 | 3.5 | -2.2 | N/A^d^ | -0.6 | N/A^d^ |
| PCSK9 | ENST00000302118 | 34 | 61 | 5.5 | -2.2 | -6.8 | -0.8 | -3.2 |
| HSD17B111 | ENST00000358290 | 16 | 18 | 9.0 | -1.8 | -6.2 | -0.6 | -2.5 |
| MST1 | ENST00000449682 | 39 | 49 | 2.3 | -1.8 | -4.6 | -0.9 | -3.8 |
| LTBP3 | ENST00000301873 | undef^c^ | 121 | 2.3 | -1.8 | -5.1 | -0.7 | -2.8 |
| RPL27 | ENST00000253788 | 26 | 28 | 9.2 | -1.6 | -7.2 | -0.5 | -2.1 |
| CDH1 | ENST00000261769 | 729 | 742 | 2.5 | -1.4 | -3.0 | +0.1 | +0.4 |
| PCBP1^b^ | ENST00000303577 | 32 | 34 | 5.2 | -1.4 | -6.3 | -0.4 | -2.3 |
| DPCD | ENST00000370151 | undef^c^ | 41 | 2.7 | -1.3 | -3.4 | -0.2 | -1.3 |
| BCAP31 | ENST00000345046 | 24 | 38 | 4.3 | -1.2 | -5.6 | -0.5 | -3.0 |
| PODXL2 | ENST00000342480 | 31 | 172 | 4.3 | -1.2 | -4.2 | -0.3 | -1.3 |
| PLA2G15 | ENST00000219345 | 38 | 42 | 3.9 | -1.2 | -2.6 | -0.6 | -1.3 |
| USO1 | ENST00000514213 | 298 | 344 | 4.6 | -1.1 | -4.4 | -0.1 | 0.0 |
| SUMF1 | ENST00000272902 | 31 | 50 | 3.2 | -1.1 | -2.7 | -0.3 | -0.5 |
| EMC7 | ENST00000256545 | 17 | 31 | 4.7 | -1.1 | -4.7 | -0.3 | -1.5 |
| CD99 | ENST00000624481 | 30 | 44 | 5.8 | -1.1 | -4.5 | -0.4 | -2.1 |
| SUMF2 | ENST00000434526 | 41 | 81 | 4.4 | -1.0 | -3.4 | -0.1 | -0.3 |
| PCBP2^b^ | ENST00000359282 | 13 | 34 | 5.4 | -0.9 | -5.3 | -0.2 | -1.3 |
| NAGA | ENST00000402937 | undef^c^ | 107 | 2.4 | -0.9 | -2.0 | -0.2 | -1.0 |
| BRI3BP | ENST00000341446 | 19 | 47 | 2.3 | -0.9 | -2.9 | -0.3 | -1.6 |
| IFI30 | ENST00000407280 | 92 | 95 | 4.1 | -0.6 | -3.5 | -0.1 | -1.1 |

*^a^*Log_2_-fold change values calculated using reads mapping 3’ to the D_max_ position, relative to the vehicle control.

*^b^*PCBP1 and PCBP2 have highly similar sequences, and most reads are ambiguously mapped between them.

^c^undef: a clear major stalling site is not observed but there is a D_max_ Z-score > 2 and a statistically significant change in downstream read counts (FDR < 10%).

^d^Raw read count for CCR1 is less then the cutoff used for inclusion in Z-score analyses.
